# An Essential Role for the Tetraspanin LHFPL4 in the Cell-Type-Specific Targeting and Clustering of Synaptic GABA_A_ Receptors

**DOI:** 10.1016/j.celrep.2017.09.025

**Published:** 2017-10-03

**Authors:** Elizabeth C. Davenport, Valentina Pendolino, Georgina Kontou, Thomas P. McGee, David F. Sheehan, Guillermo López-Doménech, Mark Farrant, Josef T. Kittler

**Affiliations:** 1 Department of Neuroscience, Physiology and Pharmacology, University College London, Gower Street, London WC1E 6BT, UK

**Keywords:** GABA_A_ receptor, LHFPL4 knockout, GARLH, accessory protein, LHFPL3, plasticity, interneuron, neuroligin, mIPSC, gephyrin

## Abstract

Inhibitory synaptic transmission requires the targeting and stabilization of GABA_A_ receptors (GABA_A_Rs) at synapses. The mechanisms responsible remain poorly understood, and roles for transmembrane accessory proteins have not been established. Using molecular, imaging, and electrophysiological approaches, we identify the tetraspanin LHFPL4 as a critical regulator of postsynaptic GABA_A_R clustering in hippocampal pyramidal neurons. LHFPL4 interacts tightly with GABA_A_R subunits and is selectively enriched at inhibitory synapses. In LHFPL4 knockout mice, there is a dramatic cell-type-specific reduction in GABA_A_R and gephyrin clusters and an accumulation of large intracellular gephyrin aggregates in vivo. While GABA_A_Rs are still trafficked to the neuronal surface in pyramidal neurons, they are no longer localized at synapses, resulting in a profound loss of fast inhibitory postsynaptic currents. Hippocampal interneuron currents remain unaffected. Our results establish LHFPL4 as a synapse-specific tetraspanin essential for inhibitory synapse function and provide fresh insights into the molecular make-up of inhibitory synapses.

## Highlights

• LHFPL4 is a tetraspanin enriched at inhibitory synapses that complexes with GABA_A_Rs
• LHFPL4 is important for GABA_A_R clustering both in vitro and in vivo
• LHFPL4 is required for the surface clustering but not the trafficking of GABA_A_Rs
• GABAergic synaptic inputs on CA1 pyramidal neurons, but not interneurons, require LHFPL4

## Graphical Abstract

**Figure:**
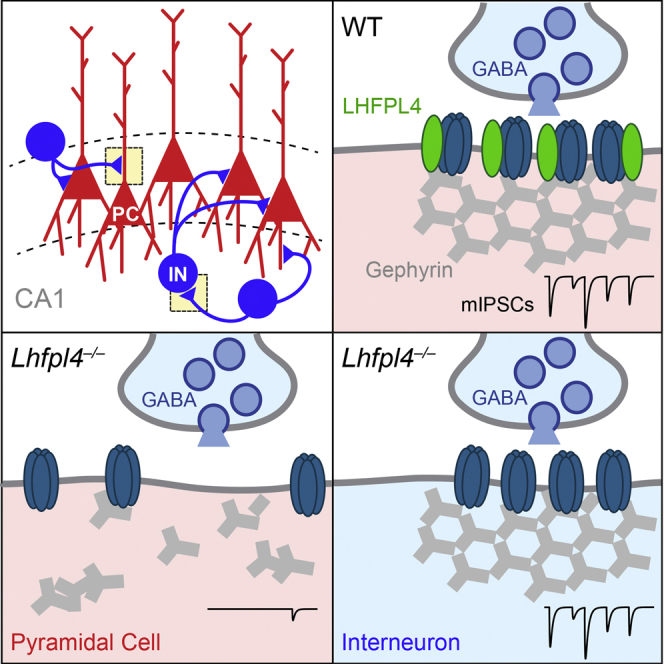

## Teaser

Davenport et al. identify LHFPL4 as a transmembrane protein that interacts with GABA_A_Rs and is essential for their synaptic clustering. Deletion of LHFPL4 results in dramatic cell-type-specific deficits in inhibitory synaptic transmission.

## Introduction

Synaptic inhibition mediated by GABA_A_ receptors (GABA_A_Rs) regulates the balance of excitation and inhibition in the brain and, thus, plays a critical role in information processing. The stabilization of synaptic GABA_A_Rs opposite GABA-releasing presynaptic terminals is crucial for efficient synaptic inhibition, appropriate regulation of circuit excitability, and animal behavior (Charych et al., 2009, Crestani et al., 1999, Luscher et al., 2011a, Papadopoulos et al., 2007). Changing the number of postsynaptic GABA_A_Rs can rapidly control the strength of inhibitory synapses. This is achieved by the trafficking of receptors to, and their removal from, the plasma membrane and by their surface lateral diffusion into and out of synaptically stabilized clusters (Bannai et al., 2009, Luscher et al., 2011b, Muir et al., 2010, Twelvetrees et al., 2010). At the synapse, GABA_A_R clustering and anchoring are mediated by a complex inhibitory postsynaptic density, the major constituent of which is the hexameric scaffold gephyrin (Tyagarajan and Fritschy, 2014). However, in the absence of gephyrin, subsets of inhibitory synapses remain (Essrich et al., 1998, Kneussel et al., 1999, O’Sullivan et al., 2009), and genetic deletion of gephyrin in the CNS has an unexpectedly subtle effect on inhibitory synaptic transmission (Lévi et al., 2004). These observations suggest the existence of as-yet-unidentified molecules important for stabilizing GABA_A_Rs at synapses.

Several membrane-spanning adhesion molecules, including neuroligin2, slitrk3, and calsyntenin3 (Pettem et al., 2013, Poulopoulos et al., 2009, Takahashi et al., 2012), contribute to the formation and stabilization of GABAergic synapses. However, virtually nothing is known regarding roles for proteins that might function as transmembrane GABA_A_R accessory proteins. In the case of ionotropic glutamate receptors, various membrane-spanning receptor-associated proteins have emerged as key regulators of receptor trafficking, synaptic targeting, and receptor function. These include transmembrane α-amino-3-hydroxy-5-methyl-4-isoxazolepropionic acid receptor (AMPAR) regulatory proteins (TARPs; γ-2, -3, -4, -5, -7, and -8), cornichons (CNIH1 and CNIH2), neuropilin and tolloid-like proteins (NETO1 and NETO2), and GSG1L (reviewed in Copits and Swanson, 2012, Haering et al., 2014, Jackson and Nicoll, 2011). Notably, TARPs and GSG1L are members of the tetraspanin superfamily of transmembrane proteins and have been shown to associate with and regulate AMPARs. Whether undiscovered tetraspanin-like molecules act to similarly coordinate GABA_A_R trafficking, synaptic stability, and function remains unknown.

Here, using biochemical, imaging, mouse transgenic, and electrophysiological approaches, we demonstrate a critical role for the previously uncharacterized tetraspanin LHFPL4 (Lipoma HMGIC Fusion Partner-Like 4) in driving the surface clustering of GABA_A_Rs at inhibitory synapses. LHFPL4 is exquisitely targeted to inhibitory synapses and forms high-affinity interactions with GABA_A_R subunits. In the absence of LHFPL4, GABA_A_Rs can still reach the cell surface but are no longer synaptically anchored, leading to a loss of inhibitory postsynaptic currents. We find that LHFPL4 acts in a cell-type-specific manner within the hippocampus, with excitatory pyramidal cells but not inhibitory interneurons affected by its deletion. Our identification of a new machinery for synaptic targeting of GABA_A_Rs opens up new avenues for understanding the construction and regulation of inhibitory synapses in the brain.

## Results

### LHFPL4 Is Targeted to Inhibitory Synapses and Interacts with GABA_A_Rs

**Figure 1:**
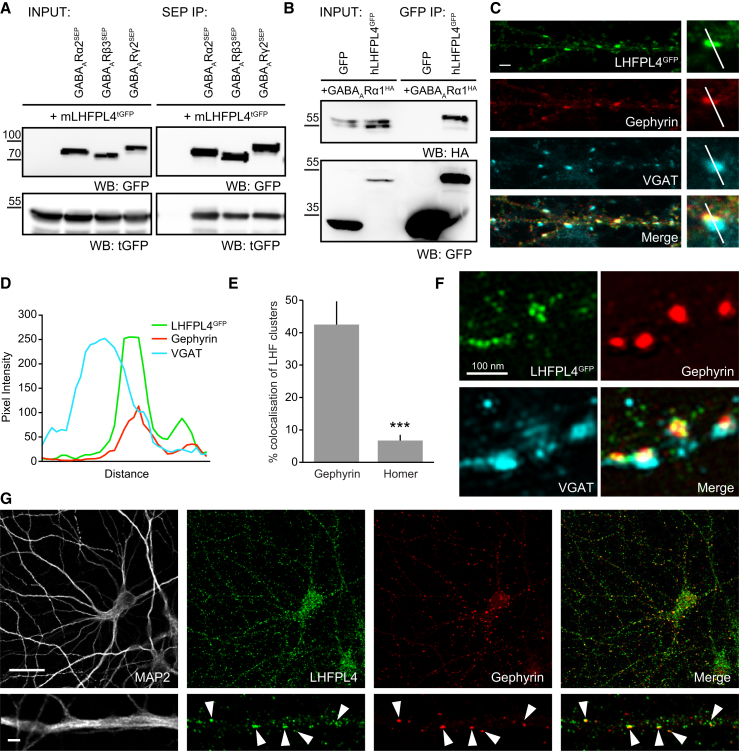
LHFPL4 Specifically Localizes to Inhibitory Synapses (A) Co-immunoprecipitation (coIP) of mouse LHFPL4-turboGFP (tGFP) with super-ecliptic-pHlourin (SEP)-tagged α2, β3, and γ2 GABA_A_R subunits from transfected COS7 cells (WB, western blot; IP, immunoprecipitation). (B) CoIP of human LHFPL4-GFP (hLHFPL4) with hemagglutinin (HA)-tagged GABA_A_R-α1 from transfected COS7 cells (WB, western blot; IP, immunoprecipitation). (C) Confocal images of dissociated rat hippocampal neurons transfected with LHFPL4^GFP^ and labeled with antibodies against gephyrin and VGAT reveal a close association between LHFPL4^GFP^ and gephyrin opposed to VGAT. Scale, 2 μm. (D) Graph shows a fluorescence intensity line scan through a synaptic cluster (white lines in C) for each channel as a function of distance. (E) Quantification of LHFPL4^GFP^ clusters colocalized with gephyrin compared to homer puncta, generated from transfected hippocampal neurons and labeled with antibodies against gephyrin and homer (gephyrin: 42.5% ± 4.4%; homer: 6.7% ± 1.4%; n = 12 cells from 2 independent preparations; p < 0.001, Welch t test). Error bars indicate mean ± SEM. ^∗∗∗^p < 0.001. (F) Single-section SIM zoom images of hippocampal neurons transfected with LHFPL4^GFP^ and labeled with antibodies against gephyrin and VGAT. (G) Confocal images of hippocampal neurons labeled with antibodies to endogenous LHFPL4, MAP2, and gephyrin reveal LHFPL4 overlaps with inhibitory synapses (white arrowheads). Scale, 20 μm (top) and 2 μm (bottom). See also Figure S1.

Although recent mass spectrometry studies have identified a number of candidate GABA_A_R-interacting proteins (Heller et al., 2012, Nakamura et al., 2016), the biochemical validation and functional role of the majority of these putative partners remain undetermined. We noted with interest the identification of LHFPL4, a predicted member of the tetraspanin superfamily of transmembrane proteins of unknown function. We initially tested the ability of LHFPL4 to interact with GABA_A_R subunits. Mouse turbo-GFP-tagged LHFPL4 (mLHFPL4^tGFP^) could be readily co-immunoprecipitated with GABA_A_R subunits (α2, β3, and γ2) from lysates of co-transfected COS-7 cells, suggesting that other neuronally expressed synaptic proteins, such as gephyrin, are not essential for the interaction. In the reverse experiment, GFP-tagged human LHFPL4 (hLHFPL4^GFP^) could also readily co-immunoprecipitate the GABA_A_R-α1 subunit from COS-7 cell lysates (Figures 1A and 1B).

To determine whether LHFPL4 was present at synaptic sites, we examined the subcellular localization of LHFPL4^GFP^ in cultured rat hippocampal neurons. Using laser scanning confocal microscopy (LSCM), LHFPL4^GFP^ was observed to form discrete membrane clusters on the soma and throughout the dendrites. These robustly colocalized with the inhibitory postsynaptic marker gephyrin opposite vesicular GABA transporter (VGAT)-labeled inhibitory presynaptic terminals. Line scans through LHFPL4^GFP^ clusters revealed peak fluorescence essentially overlapping with gephyrin and adjacent to the peak of VGAT fluorescence (Figures 1C, 1D, and S1A). Co-labeling of LHFPL4^GFP^ with gephyrin and the excitatory synaptic postsynaptic density (PSD) protein homer revealed that LHFPL4^GFP^ clusters were 6-fold more enriched at inhibitory compared to excitatory synapses (Figures 1E and S1B). We next explored the postsynaptic distribution of LHFPL4 at inhibitory synaptic sites, using structured illumination microscopy (SIM) to overcome the resolution limit of conventional fluorescence microscopy. SIM imaging of LHFPL4^GFP^ co-labeled with antibodies to gephyrin and VGAT revealed LHFPL4^GFP^ to form groups of nano-clusters overlaying gephyrin puncta (Figures 1F and S1C), further supporting an inhibitory postsynaptic localization for LHFPL4. Importantly, immunolabeling with an LHFPL4-specific antibody demonstrated that endogenous LHFPL4 is selectively enriched at gephyrin-labeled inhibitory synapses (Figure 1G). Together, these data indicate that LHFPL4 intimately associates with GABA_A_Rs and is specifically enriched at inhibitory postsynaptic domains.

### LHFPL4 Is Essential for the Clustering of GABA_A_Rs but Not Their Surface Delivery

**Figure 2:**
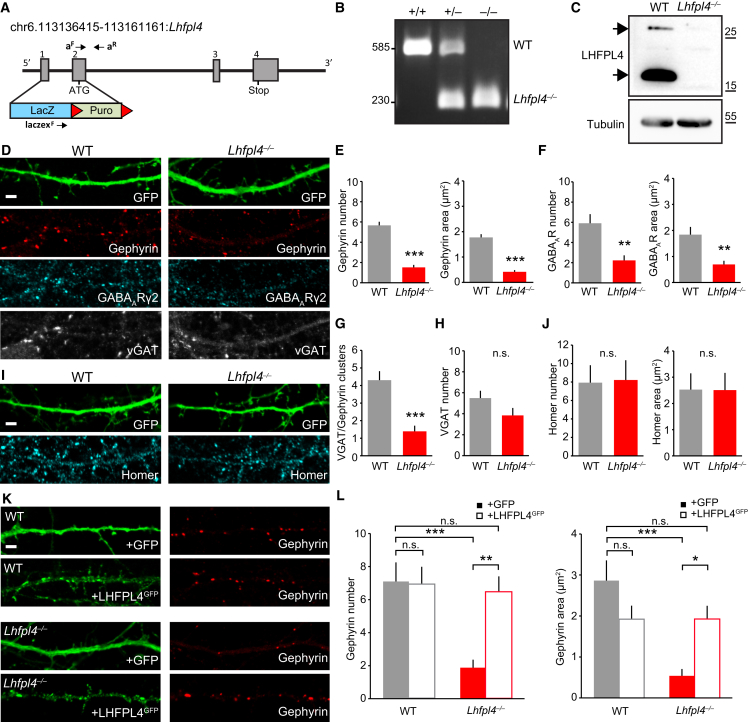
Loss of Inhibitory Synapse Stability and GABA_A_R Clustering in *Lhfpl4*^−/−^ Neurons (A) Schematic of the LHFPL4 knockout genetic strategy showing the genotyping primer sites. (B) Genotyping results from WT (+/+), heterozygous (+/−) and homozygous (−/−) animals. The product of primers a^F^ and a^R^ generates a 585-bp band from the WT allele, and the product of laczex^F^ and a^R^ generates a 230-bp band from the *Lhfpl4*^−/−^ allele. (C) Western blotting of LHFPL4 and tubulin from WT and *Lhfpl4*^−/−^ mouse brain lysates. Arrowheads indicate the two LHFPL4-specific bands detected. (D and I) Confocal images of dissociated DIV14 (14 days in vitro) WT or *Lhfpl4*^−/−^ hippocampal neurons transfected with GFP and labeled with antibodies to (D) gephyrin, GABA_A_R-γ2, and VGAT or (I) homer. (E–H and J) Quantification of (E) gephryin, (F) GABA_A_R-γ2, (G) VGAT/gephyrin, (H) VGAT and (J) homer synaptic clusters. Cluster number and total immunolabeled area were significantly reduced in *Lhfpl4*^−/−^ neurons, compared to WT neurons, when labeled for gephyrin (cluster number: from 5.7 ± 0.4 to 1.5 ± 0.2; area: from 1.8 μm^2^ ± 0.1 μm^2^ to 0.4 μm^2^ ± 0.1 μm^2^; 484/166 WT/*Lhfpl4*^−/−^ clusters; both p < 0.0001) and GABA_A_R-γ2 (cluster number: from 5.9 ± 0.9 to 2.2 ± 0.5; area: from 1.8 μm^2^ ± 0.3 μm^2^ to 0.7 μm^2^ ± 0.1 μm^2^; 636/327 WT/*Lhfpl4*^−/−^ clusters; p = 0.0011 and 0.0013, respectively). VGAT/gephyrin-positive clusters were significantly reduced in *Lhfpl4*^−/−^ neurons (from 4.3 ± 0.5 to 1.4 ± 0.3; 294/95 WT/*Lhfpl4*^−/−^ clusters; p < 0.0001). VGAT cluster number did not significantly change (from 5.5 ± 0.7 to 3.8 ± 0.7; 531/403 WT/*Lhfpl4*^−/−^ clusters; p = 0.103). In each case, n = 23 WT and 23 *Lhfpl4*^−/−^ cells from 3 independent preparations. For WT and *Lhfpl4*^−/−^ clusters, there was no significant change in homer cluster number (8.0 ± 1.9 and 8.2 ± 2.1, respectively; 333/345 WT/*Lhfpl4*^−/−^ clusters) or area (2.5 μm^2^ ± 0.6 μm^2^ and 2.5 μm^2^ ± 0.7 μm^2^, respectively; n = 14 WT and KO cells from 3 independent preparations; p = 0.92 and 0.98, respectively). All used Welch t tests. (K and L) Confocal images (K) and cluster quantification (L) of GFP or LHFPL4^GFP^ transfected WT or *Lhfpl4*^−/−^ neurons labeled with a gephyrin antibody. LHFPL4^GFP^ overexpression completely rescues the reduction of gephyrin cluster number (+GFP: WT, 7.1 ± 1.2; *Lhfpl4*^−/−^, 1.9 ± 0.5; 291/80 WT/*Lhfpl4*^−/−^ clusters; +LHFPL4: WT, 6.9 ± 1.1; *Lhfpl4*^−/−^, 6.5 ± 0.9; 291/266 WT/*Lhfpl4*^−/−^ clusters) and area (+GFP: WT, 2.9 μm^2^ ± 0.5 μm^2^; *Lhfpl4*^−/−^, 0.5 μm^2^ ± 0.2 μm^2^; +LHFPL4: WT, 1.9 μm^2^ ± 0.3 μm^2^; *Lhfpl4*^−/−^, 1.9 μm^2^ ± 0.3 μm^2^) seen in *Lhfpl4*^−/−^ neurons. n = 14 cells per condition from 3 independent preparations: p < 0.0001 for cluster number and cluster area. Kruskal-Wallis one-way ANOVA. Asterisks indicate results of Dunn’s multiple comparison tests. ^∗^p < 0.05; ^∗∗^p < 0.01; ^∗∗∗^p < 0.001; n.s., not significant. In (E)–(H), (J), and (L), error bars indicate mean ± SEM. Scale, 2 μm.

To investigate the consequences of LHFPL4 loss on the targeting of GABA_A_Rs to inhibitory synapses, we characterized neurons from a constitutive LHFPL4 knockout (KO) mouse (*Lhfpl4*^−/−^) (Figures 2A–2C). These animals were viable until adulthood, were fertile, and showed no obvious behavioral differences from wild-type (WT) animals. A band at the expected molecular weight for LHFPL4 (27 kDa) was detected from WT, but not *Lhfpl4*^−/−^, brain lysate by western blotting, and a further strong LHFPL4-specific band was detected at ∼17 kDa, which may represent a second isoform or a cleaved product (Figure 2C). Hippocampal neurons from WT and *Lhfpl4*^−/−^ mice were transfected with GFP to reveal cell morphology and were fixed and labeled with an antibody specific to a surface epitope on the GABA_A_R-γ2 subunit before being permeabilized and labeled with antibodies against gephyrin and VGAT (Smith et al., 2014). Quantification of LSCM images revealed a dramatic loss of both gephyrin and GABA_A_R-γ2 clustering and a marked decrease in VGAT-positive clusters co-labeled for gephyrin in *Lhfpl4*^−/−^ neurons compared to WT (Figures 2D–2G). By contrast, clustering of VGAT alone and homer were unchanged in *Lhfpl4*^−/−^ neurons, indicating that inhibitory presynaptic terminals and excitatory synapses were unaffected (Figures 2H–2J). Importantly, the loss of gephyrin clustering in *Lhfpl4*^−/−^ neurons could be robustly rescued upon overexpression of LHFPL4^GFP^, while LHFPL4^GFP^ had no effect on synaptic number or area when overexpressed on the WT background (Figures 2K and 2L).

**Figure 3:**
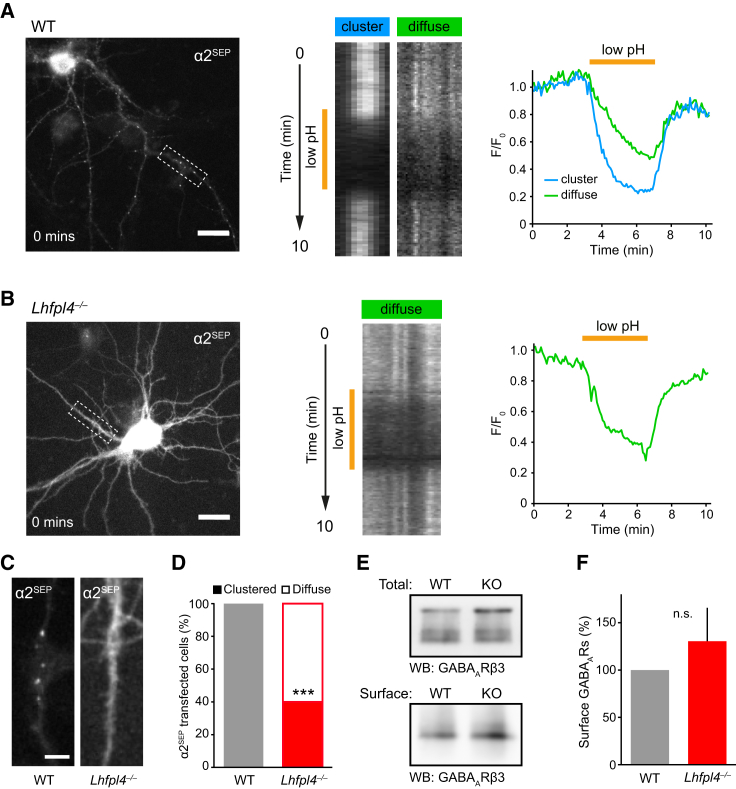
LHFPL4 Is Essential for GABA_A_R Clustering but Not Their Surface Delivery (A and B) Bright-field images of (A) WT and (B) *Lhfpl4*^−/−^ hippocampal neurons transfected with super-ecliptic pHlourin-tagged GABA_A_R-α2 (α2^SEP^). Kymograph and graph representations of 10-min movies showing pH-dependent α2^SEP^ clusters or diffuse fluorescence signal from dendrite boxed in image. Fluorescence from dendrites is eclipsed on a transient switch to low-pH imaging solution and returns on a switch back to pH 7.4, indicating that the α2^SEP^ fluorescence signal is from surface receptors. Scale, 20 μm. (C) Dendritic zooms of bright-field images of WT and *Lhfpl4*^−/−^ hippocampal dendrites transfected with GABA_A_R-α2^SEP^. GABA_A_R-α2^SEP^-containing receptors do not cluster on the surface of *Lhfpl4*^−/−^ neurons. Scale, 5 μm. (D) Quantification of GABA_A_R-α2^SEP^ surface fluorescence as clustered or diffuse (n = 50–53 cells from 3 independent preparations; p < 0.0001, Fisher’s exact test). (E) Surface biotinylations of WT and *Lhfpl4*^−/−^ neurons analyzed by western blotting with anti-GABA_A_R-β3. (F) Densiometric quantification showing no significant change in surface GABA_A_R-β3 normalized to total levels (100% and 130.4% ± 35.4%; n = 11 experiments; p = 0.41, Welch t test). Error bars indicate mean ± SEM. ^∗^p < 0.05; ^∗∗^p < 0.01; ^∗∗∗^p < 0.001. See also Figure S2.

To determine whether the loss of synaptic GABA_A_R clusters in *Lhfpl4*^−/−^ neurons was due to a disruption in GABA_A_R surface trafficking (Twelvetrees et al., 2010) or to altered synaptic targeting of surface-trafficked receptors (Muir et al., 2010), we next performed live imaging of a super-ecliptic pHluorin (SEP)-tagged GABA_A_R subunit (Muir and Kittler, 2014, Pathania et al., 2014). We used GABA_A_R-α2^SEP^, as this construct is easily expressed, assembles with endogenous GABA_A_R subunits, and demonstrates the expected pattern of surface fluorescence (Muir et al., 2010). Thus, when transfected into WT neurons, GABA_A_R-α2^SEP^ formed bright fluorescent clusters along the dendrites with lower levels of diffuse labeling, consistent with previous reports (Eckel et al., 2015, Muir et al., 2010, Tretter et al., 2008). By contrast, in the majority of transfected *Lhfpl4*^−/−^ cells, GABA_A_R-α2^SEP^ clustering was absent, with only diffuse fluorescence present throughout the soma and dendrites (Figures 3A–3C). Blind scoring of the clustered or diffuse nature of GABA_A_R-α2^SEP^ fluorescence revealed significant loss of clustering in neurons cultured from *Lhfpl4*^−/−^ mice (Figure 3D). Importantly, both in WT and *Lhfpl4*^−/−^ neurons, the GABA_A_R-α2^SEP^ fluorescence was rapidly and reversibly eclipsed by transient exposure to extracellular buffer of low pH (Figures 3A and 3B), confirming that the fluorescent signal originated from cell-surface receptors. Furthermore, biotinylation experiments showed that surface levels of endogenous GABA_A_Rs were unchanged in *Lhfpl4*^−/−^ neurons compared to WT (Figures 3E and 3F). These data suggest that loss of LHFPL4 does not interfere with the trafficking of GABA_A_Rs to the cell surface but, in a majority of cells, disrupts their tethering in synaptic clusters.

### LHFPL4 Is Not Synaptogenic

Many inhibitory postsynaptic transmembrane molecules, when overexpressed in non-neuronal cells maintained with dissociated neurons, can induce the formation of hemi-synapses by aggregating presynaptic proteins at the point of contact between the two cell types (Fuchs et al., 2013, Scheiffele et al., 2000, Takahashi et al., 2012). To test whether LHFPL4 shared these synaptogenic properties, we co-cultured COS-7 cells (overexpressing recombinant putative synaptogenic transmembrane proteins) with dissociated WT rat hippocampal neurons. After 24 hr, the cells were fixed and labeled with antibodies against VGAT and vesicular glutamate transporter (VGLUT) to identify inhibitory and excitatory hemi-synapses, respectively. Consistent with previous reports (Chih et al., 2006, Scheiffele et al., 2000), COS-7 cells overexpressing neuroligin2 induced the formation of both inhibitory and excitatory hemi-synapses (Figure S2). By contrast, in cells overexpressing GFP-tagged LHFPL4, the prevalence of inhibitory or excitatory hemi-synapses did not differ from that seen in control cells expressing GFP alone (Figure S2). Thus, LHFPL4 does not have synaptogenic properties.

### Loss of LHFPL4 Leads to Reduced GABA_A_R Clustering and Aggregates of Mis-localized Gephyrin in Intact Brain

**Figure 4:**
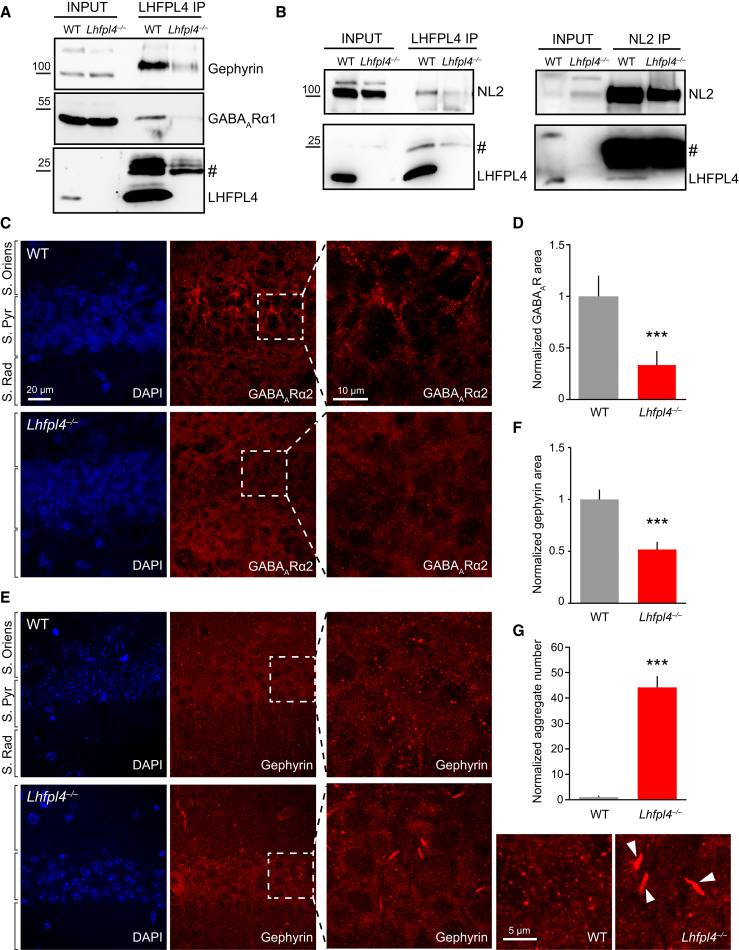
LHFPL4 Is Necessary for Gephyrin and GABA_A_R Clustering in Intact Brain (A) Co-immunoprecipitation (coIP) of gephyrin and GABA_A_R-α1 using an anti-LHFPL4 antibody from WT and *Lhfpl4*^−/−^ mouse brain lysates (IP, immunoprecipitation; #, immunoglobulin G [IgG] light chain). Note that the immunoprecipitated 27-kDa band is visible just above the IgG light chain in the WT IP lane. (B) CoIP of neuroligin2 (NL2) using an anti-LHFPL4 antibody (left) and coIP of LHFPL4 using an anti-NL2 antibody (right) from WT and *Lhfpl4*^−/−^ mouse brain lysates (IP, immunoprecipitation; #, IgG light chain). (C–F) Confocal images of adult WT and *Lhfpl4*^−/−^ hippocampal brain sections immunolabeled with antibodies to (C) GABA_A_R-α2 and (E) gephyrin, co-stained with DAPI. Normalized total cluster area quantification of (D) GABA_A_R-α2 and (F) gephyrin showing a loss of GABA_A_R-α2 (from 1.0 ± 0.2 to 0.3 ± 0.1; n = 17 WT and 19 KO hippocampi from 4 animals per genotype; p = 0.0083) and gephyrin (from 1.0 ± 0.1 to 0.5 ± 0.1; n = 16 WT and 12 KO hippocampi from 3 animals per genotype; p = 0.0004) clustering in *Lhfpl4*^−/−^ tissue. (G) Bar graph showing increased large gephyrin aggregates in *Lhfpl4*^−/−^ compared to WT brain slices (from 1.0 ± 0.6 to 44.2 ± 4.4; n = 15 WT and 14 KO regions from 3 animals per genotype; p < 0.0001). White arrowheads indicate gephyrin aggregates in the zoom image. In (D), (F), and (G), error bars indicate mean ± SEM. ^∗^p < 0.05; ^∗∗∗^p < 0.001 (Welch t tests).

We further explored the relationship between GABA_A_Rs and LHFPL4 in whole brain samples. Importantly, an LHFPL4 antibody readily co-immunoprecipitated the GABA_A_R-α1 subunit, gephyrin, and the inhibitory postsynaptic adhesion molecule neuroligin2 from WT but not *Lhfpl4*^−/−^ brain lysate. In addition, LHFPL4 could be co-immunoprecipitated with a neuroligin2 antibody in the reciprocal experiment (Figures 4A and 4B). Thus, LHFPL4 can form native complexes in vivo with key components of the inhibitory scaffold in addition to GABA_A_Rs.

To address how loss of LHFPL4 affected GABA_A_R clustering and inhibitory synapse integrity in the intact brain, we carried out immunohistochemistry on fixed brain sections from adult WT and *Lhfpl4*^−/−^ mice. Consistent with the loss of GABA_A_R clustering seen in cultured neurons, labeling with a GABA_A_R-α2 antibody revealed a dramatic decrease in total GABA_A_R cluster area in the hippocampal CA1 region of *Lhfpl4*^−/−^ mice (Figures 4C and 4D). Labeling with a gephyrin antibody revealed a significant loss of total gephyrin cluster area (Figures 4E and 4F), indicating a parallel disruption of the inhibitory postsynaptic domain. The inhibitory presynaptic domain, revealed by labeling with glutamate decarboxylase (GAD6) antibody, remained intact (Figures S3A and S3B). Remarkably, we also observed the presence of large aggregates of mis-localized gephyrin within the soma and dendrites of *Lhfpl4*^−/−^ neurons (Figures 4E and 4G), associated with a significant decrease in GAD6/gephyrin-positive clusters (Figure S3C). The dramatic re-distribution of gephyrin, along with the loss of GABA_A_R-α2 subunit clustering, is consistent with LHFPL4 playing a key role in maintaining GABA_A_Rs and their associated scaffold at inhibitory synaptic sites.

### Loss of LHFPL4 Disrupts Inhibitory Postsynaptic Currents in Cultured Neurons

**Figure 5:**
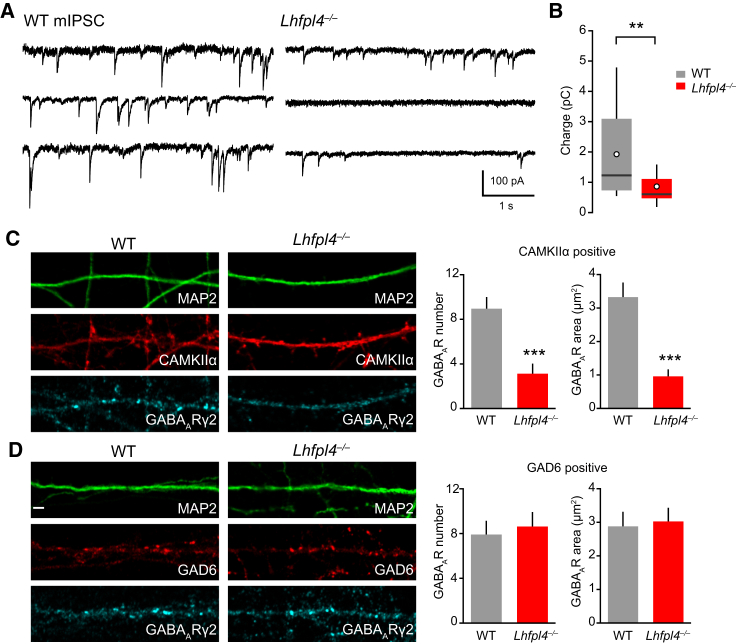
Selective Loss of mIPSCs in Cultured *Lhfpl4*^−/−^ Neurons (A) Representative recordings of mIPSCs (−70 mV) in cultured hippocampal neurons prepared from WT mice (left) and *Lhfpl4*^−/−^ mice (right). In each case, recordings from three different neurons are shown. Records digitally filtered at 2 kHz for illustration purposes. (B) Pooled data showing reduction in mean mIPSC charge transfer (from 1.93 pC ± 0.37 pC to 0.86 pC ± 0.12 pC; n = 18 WT and 24 *Lhfpl4*^−/−^ cells; p = 0.0058, Wilcoxon rank-sum test). Box-and-whisker plots indicate median (line), 25th–75th percentiles (box), the range of data within 1.5 × interquartile range (IQR) of box (whiskers), and mean (open circles). (C and D) Confocal images and cluster quantification of dissociated DIV14 WT or *Lhfpl4*^−/−^ hippocampal neurons transfected with GFP and labeled with antibodies to GABA_A_R-γ2 and either (C) CAMKIIα or (D) GAD6 to label excitatory neurons and inhibitory neurons, respectively. GABA_A_R cluster number and area were significantly reduced in *Lhfpl4*^−/−^ CAMKIIα-positive cells (cluster number: from 8.9 ± 1.0 to 3.1 ± 0.9; area: from 3.3 μm^2^ ± 0.4 μm^2^ to 1.0 μm^2^ ± 0.2 μm^2^; 362/131 WT/*Lhfpl4*^−/−^ clusters; p = 0.00032 and 0.00013, respectively), but not GAD6-positive cells, compared to WT (cluster number: from 7.9 ± 1.2 to 8.6 ± 1.3; area: from 2.9 μm^2^ ± 0.4 μm^2^ to 3.0 μm^2^ ± 0.4 μm^2^; 376/362 WT/*Lhfpl4*^−/−^ clusters; p = 0.69 and 0.81, respectively). Error bars indicate mean ± SEM. For all conditions, n = 14 cells from 3 independent preparations. All used the Welch t test. ^∗∗^p < 0.01, ^∗∗∗^p < 0.001. Scale, 2 μm.

To determine the functional effect of LHFPL4 deletion, we initially examined hippocampal neurons in dissociated cultures and measured charge transfer mediated by miniature inhibitory postsynaptic currents (mIPSCs). When compared to neurons from WT littermates, synaptic charge in *Lhfpl4*^−/−^ neurons was reduced by ∼60% (Figures 5A and 5B), indicating loss of receptor/synapse number or function. Of note, we observed considerable variability in the amplitude and frequency of mIPSCs in both WT and *Lhfpl4*^−/−^ cultures. This could reflect heterogeneity in the mixed hippocampal preparations and a varied contribution of LHFPL4. Indeed, immunolabeling for VGAT and GABA_A_R-γ2 revealed a small population of neurons cultured from *Lhfpl4*^−/−^ mice that appeared to maintain their inhibitory synapses, while GABA_A_R-α2^SEP^ fluorescence also remained clustered in a proportion of cells. These observations suggested that LHFPL4 effects may be cell type specific. To test this, we compared the effect of LHFPL4 deletion on GABA_A_R-γ2 clustering in excitatory and inhibitory neurons, identified using antibodies against CAMKIIα and GAD6, respectively. Whereas CAMKIIα-positive cells showed a loss of GABA_A_R-γ2 clustering, GAD6-positive cells did not (Figures 5C and 5D). Thus, LHFPL4 appears to be essential for GABA_A_R clustering only in excitatory hippocampal neurons.

### Cell-Type- and Synapse-Specific Effects of LHFPL4 Deletion

**Figure 6:**
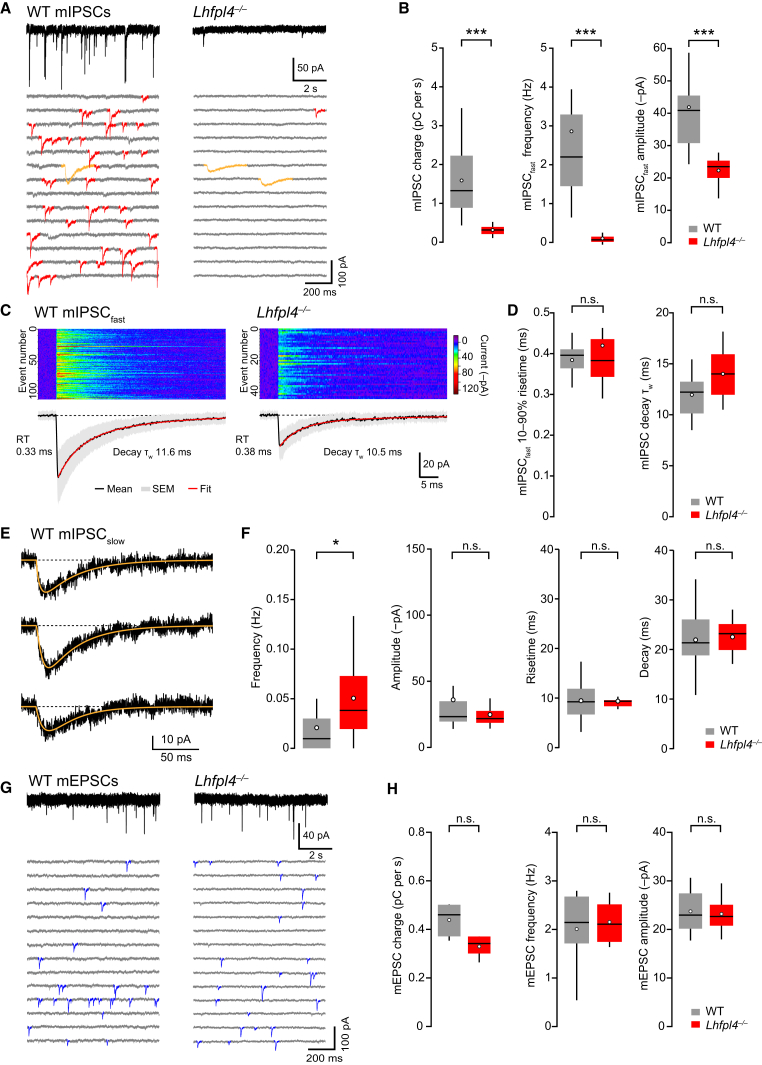
Loss of Fast mIPSCs in CA1 Pyramidal Neurons from *Lhfpl4*^−/−^ Mice (A) Representative recordings of mIPSCs (−70 mV) in CA1 pyramidal cells from a WT mouse (left) and an *Lhfpl4*^−/−^ mouse (right). Lower panels are representative sections of recordings (contiguous 1-s segments) showing a loss of fast mIPSCs (red) but a maintained presence of slow mIPSCs (orange). Records are digitally filtered at 2 kHz for illustration purposes. (B) Pooled data showing reduction in mean mIPSC charge transfer (from 1.59 pC ± 0.21 pC to 0.31 pC ± 0.11 pC; n = 18 WT and 18 *Lhfpl4*^−/−^ cells), frequency (from 2.86 Hz ± 0.54 Hz to 0.10 Hz ± 0.03 Hz; n = 18 WT and 18 *Lhfpl4*^−/−^ cells), and amplitude (from 141.9 pA ± 3.8 pA to 22.3 pA ± 1.1 pA; n = 18 WT and 14 *Lhfpl4*^−/−^ cells). All p < 0.0001, Wilcoxon rank-sum test. (C) Top: images illustrating the alignment and amplitudes of selected mIPSCs with uncontaminated rise and decay from representative WT and *Lhfpl4*^−/−^ recordings. Bottom: average mIPSC waveforms (black), SEM (gray), and fitted sum of exponentials (red). The 10%–90% rise times and weighted time constant of decay (τ_w_) are shown for each representative record. (D) Pooled data showing the lack of effect on mIPSC rise time (0.38 ms ± 0.01 ms and 0.42 ms ± 0.04 ms; n = 18 WT and 11 *Lhfpl4*^−/−^ cells; p = 0.86, Wilcoxon rank-sum test) and decay (12.0 ms ± 0.5 ms and 14.0 ms ± 1.4 ms; n = 18 WT and 11 *Lhfpl4*^−/−^ cells; p = 0.20, Welch t test). (E) Representative slow mIPSCs (−70 mV) from CA1 pyramidal neurons (three different WT mice). Orange lines indicate fits of an empirical equation (see the Supplemental Experimental Procedures) from which 10%–90% rise time and 63% decay time measures were taken. (F) Pooled data showing the modest increase in frequency of slow mIPSCs in *Lhfpl4*^−/−^ mice (from 0.021 Hz ± 0.007 Hz to 0.051 Hz ± 0.01 Hz; n = 18 WT and 18 *Lhfpl4*^−/−^ cells; p = 0.014, Wilcoxon rank-sum test) but a lack of change in amplitude (36.0 pA ± 8.4 pA and 24.8 pA ± 2.4 pA; n = 12 WT and 16 *Lhfpl4*^−/−^ cells; p = 0.37 Wilcoxon rank-sum test), rise time (9.5 ms ± 1.1 ms and 9.4 ms ± 0.4 ms; n = 12 WT and 16 *Lhfpl4*^−/−^ cells; p = 0.77, Welch t test), and decay (21.9 ms ± 1.7 ms and 22.5 ms ± 0.9 ms; n = 12 WT and 16 *Lhfpl4*^−/−^ cells; p = 0.93, Welch t test). Box-and-whisker plots indicate median (line), 25th–75th percentiles (box), the range of data within 1.5 × IQR of the box (whiskers), and mean (open circles). (G) Representative recordings of mEPSCs (−70 mV) in CA1 pyramidal cells from a WT mouse (left) and a *Lhfpl4*^−/−^ mouse (right). Lower panels are representative sections of recordings (as in A), with mEPSCs in blue. (H) Pooled data showing no change in mean mEPSC charge transfer (0.44 pC ± 0.07 pC and 0.33 pC ± 0.03 pC), frequency (2.01 Hz ± 0.35 Hz and 2.15 Hz ± 0.27 Hz), and amplitude (23.7 pA ± 2.1 pA and 23.2 pA ± 2.4 pA). In each case, n = 6 WT and 4 *Lhfpl4*^−/−^ cells (p = 0.21, 0.75, and 0.87, respectively; Welch t test). See also the Supplemental Experimental Procedures.

To further explore LHFPL4 function and its putative cell-type specificity, we made recordings in acute hippocampal slices from WT and *Lhfpl4*^−/−^ mice. LHFPL4 deletion resulted in a profound loss of mIPSCs in CA1 pyramidal neurons (Figure 6A), with the mIPSC-mediated charge transfer reduced by ∼80% (Figure 6B). Fast mIPSCs (median, 10%–90%; rise time, 0.4 ms; and τ_w, decay_, 12 ms), likely originating from perisomatically projecting basket cells (Miles et al., 1996), were markedly reduced in both frequency and amplitude (Figure 6B), although their rise and decay were not changed (Figures 6C and 6D). By contrast, slow mIPSCs (Pearce, 1993) (median, 10%–90%; rise time, 9 ms; and τ_w, decay_, 21 ms), of the type thought to originate from neurogliaform/Ivy cells (Armstrong et al., 2012, Szabadics et al., 2007), were modestly increased in frequency and unaltered in amplitude (Figures 6E and 6F). Importantly, LHFPL4 deletion had no effect on AMPAR-mediated miniature excitatory postsynaptic currents (mEPSCs) (Figures 6G and 6H), confirming a selective effect on inhibitory synapses.

**Figure 7:**
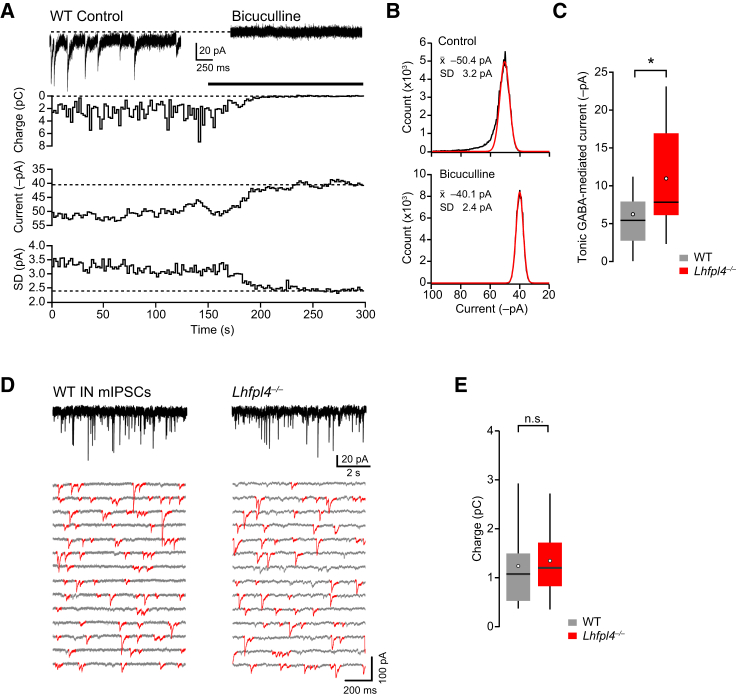
GABA-Mediated Tonic Current in Pyramidal Cells and mIPSCs in Interneurons Are Not Disrupted by LHFPL4 Deletion (A) Representative record (−70 mV) from a WT CA1 pyramidal neuron showing the block of mIPSCs and the shift in holding current produced by bath application of bicuculline. Lower panels show the time course of the synaptic charge transfer, mean holding current, and its SD (see Supplemental Experimental Procedures). (B) Representative all-point amplitude histograms from 1-s segments in the control period (upper) and in the presence of bicuculline (lower). Red lines indicate fits of a single-sided Gaussians to the most positive current values yielding the mean and the SD. The mean was taken as the baseline current for that segment and subtracted from the record. The integral of the subtracted current provided the charge carried by the synaptic events. (C) Pooled data showing increased tonic GABA-mediated current in CA1 pyramidal neurons (from 6.2 pA ± 1.3 pA to 11.0 pA ± 1.7 pA; n = 22 WT and 15 *Lhfpl4*^−/−^ cells; p = 0.037, Welch t test). (D) Representative recordings of mIPSCs (−70 mV) in non-pyramidal cells (INs, presumptive interneurons) from a WT mouse (left) and an *Lhfpl4*^−/−^ mouse (right). Lower panels are representative sections of recordings (contiguous 1-s segments) showing no change in fast mIPSCs (red). Records digitally filtered at 2 kHz for illustration purposes. (E) Pooled data showing the lack of effect of LHFPL4 deletion on mIPSC-mediated charge transfer in presumptive interneurons (1.24 pC ± 0.39 pC and 1.34 pC ± 0.34 pC; n = 6 WT and 6 *Lhfpl4*^−/−^ cells; p = 0.84, Welch t test). Box-and-whisker plots indicate median (line), 25th–75th percentiles (box), the range of data within 1.5 × IQR of the box (whiskers), and mean (open circles). ^∗^p < 0.05. See also the Supplemental Experimental Procedures.

We next examined the effect of LHFPL4 deletion on bicuculline-sensitive tonic currents reflecting the persistent activation of extrasynaptic GABA_A_Rs (Farrant and Nusser, 2005) (Figures 7A–7C). In CA1 pyramidal cells from *Lhfpl4*^−/−^ mice, the magnitude of the tonic current was slightly increased (Figure 7C), suggesting that LHFPL4 is required for the targeting of synaptic, but not extrasynaptic, GABA_A_Rs. Finally, to determine whether the functional effects of LHFPL4 were, indeed, cell type specific, we examined presumptive inhibitory interneurons. In marked contrast to the profound loss of mIPSC-mediated charge transfer seen in CA1 pyramidal cells, charge transfer in non-pyramidal cells in CA1 stratum oriens, radiatum, or lacunosum-moleculare from *Lhfpl4*^−/−^ mice was unaffected (Figures 7D and 7E).

## Discussion

As clustering of GABA_A_Rs at synapses is essential for correct inhibitory signaling in the brain (Luscher et al., 2011b, Smith and Kittler, 2010, Tyagarajan and Fritschy, 2014, Vithlani et al., 2011), it is vital to understand the machinery and regulatory pathways involved. Using molecular, imaging, electrophysiological, and mouse transgenic approaches, we show that the previously uncharacterized tetraspanin, LHFPL4, forms a complex with GABA_A_Rs, localizes to the inhibitory postsynaptic domain, and is critical for postsynaptic GABA_A_R clustering and fast GABAergic transmission in excitatory principal cells.

At inhibitory synapses, the canonical scaffold gephyrin, in complex with collybistin and the *trans*-synaptic adhesion molecule neuroligin2, plays a key role in GABA_A_R clustering and anchoring (reviewed in Tyagarajan and Fritschy, 2014, Varoqueaux et al., 2004). Nevertheless, in the absence of gephyrin, subsets of inhibitory synapses remain (Kneussel et al., 1999, O’Sullivan et al., 2009, Panzanelli et al., 2011). Moreover, the role for collybistin in inhibitory synapse formation appears to be region specific (Papadopoulos et al., 2007). Thus, there has been great interest in identifying other molecules that drive GABA_A_R clustering in a cell-type- or synapse-specific manner.

We found LHFPL4 to be enriched at inhibitory postsynaptic sites, and SIM imaging revealed clusters of LHFPL4 to overlay gephyrin clusters, placing it in the exact location required to scaffold GABA_A_Rs at the synapse. Importantly, we did not find LHFPL4 at excitatory synapses, suggesting that it is not a general synaptic organizer but rather an exclusive regulator of the inhibitory synaptic domain. This is supported by our finding that LHFPL4 and GABA_A_Rs interact with high affinity and that deletion of LHFPL4 leads to a dramatic loss of GABA_A_R and gephyrin clustering, while excitatory synapses are unaffected. It is currently thought that GABA_A_Rs are initially trafficked extrasynaptically and subsequently diffuse to, and are trapped at, synaptic sites (Bannai et al., 2009, Bogdanov et al., 2006, Luscher et al., 2011b). In *Lhfpl4*^−/−^ neurons, GABA_A_Rs were still found at the neuronal surface. This suggests that, in the absence of LHFPL4, the cell-surface GABA_A_R trafficking pathways are preserved but that, once at the cell surface, receptors can no longer be trapped at synapses.

It is well established that synaptic GABA_A_Rs are reciprocally required for the clustering of gephyrin (Tyagarajan and Fritschy, 2014). Thus, it is difficult to determine whether the loss of gephyrin clustering we observed upon LHFPL4 deletion is a cause or a consequence of the LHFPL4-dependent disruption of GABA_A_R clustering. In this regard, it has previously been described that, in hippocampal and thalamic relay neurons, subunit-specific knockout of GABA_A_Rs leads to the accumulation of large intracellular gephyrin aggregates (Studer et al., 2006). This phenomenon was attributed to the need for synaptic GABA_A_Rs to maintain gephyrin clustering (Essrich et al., 1998). Interestingly, we found that loss of LHFPL4 in vivo leads to a similar accumulation of intracellular gephyrin aggregates in the cell-body layer of the hippocampus.

Accompanying the loss of GABA_A_R clusters, in CA1 pyramidal neurons from *Lhfpl4*^−/−^ mice, we observed a dramatic decrease in both the amplitude and frequency of fast mIPSCs. The reduced amplitude of mIPSCs would, most straightforwardly, reflect loss of postsynaptic receptors. The primarily postsynaptic action of LHFPL4 is supported by the fact that, in our rescue experiments, sparse re-expression of LHFPL4 cDNA in *Lhfpl4*^−/−^ neurons readily rescued postsynaptic GABA_A_R clustering. The reduced frequency of mIPSCs could reflect both direct and indirect effects of such receptor loss; namely, a reduction of mIPSC amplitude below the threshold for detection and a possible loss of inhibitory synapses. Indeed, GABA_A_Rs have been shown to have synaptogenic properties (Fuchs et al., 2013). Surprisingly, however, we detected no significant loss of VGAT clusters in the absence of LHFPL4, suggesting that the marked reduction in mIPSC frequency is primarily due to the postsynaptic loss of receptors and not due to a loss of inhibitory input. Similarly, deletion of InSyn1, a newly identified gephyrin-interacting protein (the loss of which results in reduced postsynaptic GABA_A_R clustering), was also reported to lead to a marked reduction in mIPSC frequency (Uezu et al., 2016).

While fast mIPSCs were greatly reduced in *Lhfpl4*^−/−^ pyramidal cells, slow mIPSCs were not. The slow mIPSCs had kinetics similar to those of currents generated by inputs from neurogliaform/Ivy cells (Armstrong et al., 2012). Interestingly, tonic inhibition was also preserved in *Lhfpl4*^−/−^ neurons; indeed, it was slightly increased, possibly in response to the loss of fast phasic currents. In CA1 pyramidal neurons, both the slow IPSCs arising from neurogliaform cells and the tonic GABA-mediated currents are thought to be mediated by α5-subunit-containing GABA_A_Rs (Caraiscos et al., 2004, Karayannis et al., 2010). Thus, our results suggest that LHFPL4 may drive GABA_A_R clustering that is both synapse and subunit specific. The action of LHFPL4 could be seen as complimentary to that of radixin, which mediates extrasynaptic trapping of α5-subunit-containing GABA_A_Rs (Loebrich et al., 2006).

The postsynaptic single-pass membrane-spanning molecules neuroligin2, calsyntenin3, and slitrk3 promote the development and stabilization of mammalian GABAergic synapses through transynaptic interactions with presynaptic proteins—neurexins and PTPδ, respectively. Calsyntenin3 and slitrk3 loss of function leads primarily to a reduction in mIPSC frequency, while disruption of neuroligin2 causes both frequency and amplitude reductions in several cell types (Pettem et al., 2013, Poulopoulos et al., 2009, Takahashi et al., 2012). Although we identified LHFPL4 as a membrane-spanning inhibitory synaptic protein, we found no evidence that it could drive the formation of synapses onto transfected non-neuronal cells. Moreover, overexpression of LHFPL4 did not increase synapse formation in neurons. This suggests that LHFPL4 does not mediate GABA_A_R clustering through synapse specification and *trans*-synaptic interaction with presynaptic terminals but, rather, acts to stabilize postsynaptic GABA_A_R clusters, possibly through enhancing interactions between GABA_A_Rs and synaptogenic molecules such as neuroligin2.

Understanding the processes that mediate cell- and synapse-specific GABA_A_R clustering is key to a better understanding of the underlying logic of inhibitory control in the brain. The homophilic adhesion molecule IgSF9b was recently demonstrated to promote inhibitory synapse development in interneurons by coupling to neuroligin2 and S-SCAM (Woo et al., 2013). By contrast, we show here that the impact of LHFPL4 deletion on GABA_A_R clustering and synaptic inhibition in the hippocampus is specific to CamKIIα-positive excitatory principal cells. As we found LHFPL4 to interact tightly with neuroligin2, it is likely that neuroligins work in concert with an array of clustering molecules to drive cell-specific regulation of postsynaptic receptor clustering. In this regard, further studies of identified interneuron types will be necessary to determine whether resistance to LHFPL4 deletion is a property of all interneurons or specific classes.

As the strength of inhibitory synaptic transmission directly correlates with the number of surface GABA_A_Rs at synaptic sites, modulation of GABA_A_R synaptic accumulation is a key mechanism underlying inhibitory synaptic plasticity. It will be interesting to determine whether LHFPL4 plays a role in previously reported mechanisms of activity-dependent tuning of synaptic GABA_A_R number (Bannai et al., 2009, Muir et al., 2010, Petrini et al., 2014). Deficits in GABAergic neurotransmission can result in alterations in information processing at the network level and have been implicated in multiple neuropsychiatric disorders (Blundell et al., 2009, Charych et al., 2009, Crestani et al., 1999, Luscher et al., 2011a, Yizhar et al., 2011). Thus, identification of the molecular mechanisms by which LHFPL4 regulates inhibitory transmission may be critical to understanding both normal and disordered states. In line with other knockout mouse models that result in disrupted GABA_A_R and gephyrin clustering, such as the GABA_A_R-α1 or β2 subunit knockouts, we saw no obvious home cage behavioral deficits in *Lhfpl4*^−/−^ animals (Kralic et al., 2006, Sur et al., 2001, Vicini et al., 2001). Further detailed characterization will be necessary to identify any disease-associated behavioral deficits, such as the anxiety- and schizophrenia-related sensorimotor deficits observed in neuroligin2 and GABA_A_R-α3 knockout mice, respectively (Blundell et al., 2009, Yee et al., 2005).

Very recently, a paper describing LHFPL4 as a GABA_A_R regulatory Lhfpl (GARLH) protein was published (Yamasaki et al., 2017). Although not the first to establish LHFPL4 as a GABA_A_R-interacting protein (Heller et al., 2012, Nakamura et al., 2016), these authors identified a tripartite interaction between GABA_A_Rs, neuroligin2, and LHFPL4. Using the complimentary approaches of short hairpin RNA (shRNA)-mediated LHFPL4 knockdown in culture and virally mediated CRISPR knockout in Cre-dependent Cas9 knockin mice, they also reported a marked reduction in mIPSC frequency in hippocampal CA1 neurons. Of note, here we further demonstrate that LHFPL4 is essential for inhibitory synapse stability in CA1 pyramidal cells but not in hippocampal interneurons. Moreover, we additionally observed that fast, but not slow, mIPSCs are reduced in *Lhfpl4*^−/−^ mice, suggesting that LHFPL4 effects may be not only cell type specific but also synapse specific. It will be interesting to determine whether the modestly increased frequency of slow mIPSCs and increased tonic inhibition we identified in *Lhfpl4*^−/−^ mice are adaptive changes due to prolonged disruption of synaptic inhibition (Brickley et al., 2001).

Members of the tetraspanin superfamily of integral membrane proteins have emerged as key regulators of excitatory synaptic function. Notably, TARPs and GSG1L have been shown to associate with AMPARs to regulate their trafficking and functional properties (Jackson and Nicoll, 2011, McGee et al., 2015). Our findings provide new insights into the molecular make-up of the inhibitory PSD and reveal a key role for an inhibitory synapse-specific tetraspanin. LHFPL4 is one of a subfamily of five tetraspanins that includes the homologous LHFPL3 and LHFPL5 and the more distantly related LHFPL1 and LHFPL2. Interestingly, LHFPL5 has been implicated in the mechanotransduction pathway of the inner hair cell, and its dysfunction contributes to hearing loss in humans and mice (György et al., 2017, Kalay et al., 2006, Longo-Guess et al., 2005, Xiong et al., 2012). Whether the other family members also have roles in synaptic function remains to be determined.

## Experimental Procedures

Details regarding animals, antibodies, immunocytochemistry and immunohistochemistry, co-immunoprecipitations, biotinylations, cDNA cloning, and data analysis are included in the Supplemental Experimental Procedures.

### Cell Culture and Transfections

Hippocampal cultures were obtained from E16 mouse or E18 rat embryos of either sex, as previously described (López-Doménech et al., 2016, Vaccaro et al., 2017). All procedures for the care and treatment of animals were in accordance with the Animals (Scientific Procedures) Act 1986. Neurons were transfected using Lipofectamine 2000 (Invitrogen). COS-7 cells were maintained in DMEM supplemented with fetal calf serum and antibiotics and were transfected using the Amaxa Nucleofector device (Lonza) following the manufacturer’s protocol.

### Microscopy

Confocal images were acquired on a Zeiss LSM700 upright confocal microscope using a 63× oil objective (NA: 1.4) and digitally captured using LSM software. For cultured neurons, a whole-cell single-plane image was captured using a 0.5× zoom. From this, 3 sections of dendrite, ∼100 μm from the soma, were imaged with a 3× zoom (equating to a 30-μm length of dendrite). For brain sections from adult male and female fixed brains, a low-magnification region of the hippocampus was captured using a 63× objective and 0.5× zoom. From this, 2–3 zoom regions were imaged within each hippocampal strata with a 2× zoom for analysis. Acquisition settings and laser power were kept constant within experiments. For details of antibody labeling and image analysis, see Supplemental Experimental Procedures.

SIM was performed on a commercial Zeiss ELYRA PS.1 inverted microscope. Images were acquired with a 63× oil objective lens (NA: 1.4) using a pco.edge sCMOS camera and ZEN Black (v.11.0.2.190) software (2,430 × 2,430 pixels, 78.32-μm^2^ image size, 16 bit). Typically, images were acquired with 34-μm grating and three rotations by exciting fluorophores with 1%–3% laser intensity and 120-ms to 150-ms exposure time. Images were processed with ZEN Black using the SIM reconstruction module with default settings; drift corrections between the channels were performed with respect to 100-nm Tetraspec fluorescent microspheres (Molecular Probes).

### Electrophysiology

Standard whole-cell voltage-clamp techniques were used to record mIPSCs in cultured neurons. For details, see the Supplemental Experimental Procedures. For slice electrophysiology, hippocampal slices from male and female mice (P30–P45) were perfused at room temperature with external solution containing (in millimolar): 125 NaCl, 2.5 KCl, 1 MgCl_2_, 1.25 NaH_2_PO_4_, 2 CaCl_2_, 38 glucose, and 26 NaHCO_3_ saturated with 95% O_2_/5% CO_2_, (pH 7.4). Cells were visualized using oblique illumination. Currents were recorded using a MultiClamp 700B amplifier (Molecular Devices), filtered at 4 kHz, and digitized at 50 kHz using WinWCP and WinEDR (Strathclyde Electrophysiology Software) and an InstruTECH ITC-18 interface (HEKA Elektronik). Series resistance was typically compensated by 50%–80%, and data were discarded if the series resistance varied by >20%.

For mIPSCs and tonic current measurement, recording pipettes were filled with an internal solution containing (in millimolar): 128 CsCl, 10 EGTA-Cs, 10 HEPES, 2 MgATP, 1 CaCl_2_, 2 NaCl, 1 QX-314 (Tocris Bioscience), and 5 TEA-Cl (adjusted to pH 7.3 with CsOH). In some cases, this solution also contained 0.2% biocytin (Molecular Probes). D-AP5 (20 μM, Abcam) and NBQX (10 μM, Abcam) were added to the external solution to block NMDA receptors (NMDARs) and AMPARs. All mIPSCs were blocked by bicuculline (20 μM, Tocris) or gabazine (20 μM, Abcam). mEPSCs were recorded at 32.5°C using an internal solution containing (in millimolar) 135 Cs-gluconate, 10 HEPES, 10 Na-phosphocreatine, 4 MgATP, 0.4 NaGTP, 2 QX-314, and 10 TEA-Cl (adjusted to pH 7.3 with CsOH). The external solution contained an additional 5 mM KCl and bicuculline (20 μM) or gabazine (20 μM). All mEPSCs were blocked with NBQX (10 μM; Abcam or Tocris). Interneurons were identified under infrared video microscopy by their relatively small and rounded or ovoid cell bodies, compared to the large triangular somata of pyramidal cells, and by the absence of conspicuous apical dendrites. Their cell bodies were located in CA1 stratum oriens, radiatum, or lacunosum-moleculare. Although reconstruction was not performed, post hoc examination of each biocytin-filled putative interneuron confirmed its location outside the pyramidal cell layer and its non-pyramidal cell morphology.

### Statistics

All data were obtained from at least three different cell preparations or animals. Data are reported as mean ± SEM. Repeats for experiments are given in the figure legends as n numbers. No statistical test was used to pre-determine sample sizes; these were based on standards of the field. Statistical analyses were carried out using GraphPad Prism (GraphPad Software, La Jolla CA, USA), Microsoft Excel or R (v.3.2.3; the R Foundation for Statistical Computing; http://www.r-project.org/), and R Studio (v.0.99.893; RStudio). Data were tested for normality (D’Agostino-Pearson test or Shapiro-Wilk test) and compared using either parametric (unpaired Welch t test) or non-parametric tests (Wilcoxon rank-sum test or Kruskal-Wallis one-way ANOVA). Exact p values are presented to two significant figures, except when p < 0.0001. Differences were considered significant at p < 0.05. No blinding or randomization was used.

## Author Contributions

This study was conceived by J.T.K. Experiments were designed by J.T.K., E.C.D., and M.F. Experiments were performed by E.C.D., V.P., G.K., T.P.M., D.F.S., and G.L.-D. Data were analyzed by E.C.D., V.P., G.K., M.F., and J.T.K., and the manuscript was written by E.C.D., M.F., and J.T.K.

## Supplemental Information

Document S1. Supplemental Experimental Procedures and Figures S1–S3

Document S2. Article plus Supplemental Information
