# Supplementary material for: An Essential Role for the Tetraspanin LHFPL4 in the Cell-Type-Specific Targeting and Clustering of Synaptic GABAA Receptors
Source: Cell Rep. 2017 Oct 3;21(1):70–83. doi: 10.1016/j.celrep.2017.09.025 (PMC5640807; doi:10.1016/j.celrep.2017.09.025)
Supplement: Document S1. Supplemental Experimental Procedures and Figures S1–S3 [file mmc1.pdf]

Cell Reports, Volume *21*

Supplemental Information

An Essential Role for the Tetraspanin LHFPL4 in the Cell-Type-Specific Targeting and Clustering of Synaptic $GABA_A$ Receptors

Elizabeth C. Davenport, Valentina Pendolino, Georgina Kontou, Thomas P. McGee, David F. Sheehan, Guillermo López-Doménech, Mark Farrant, and Josef T. Kittler

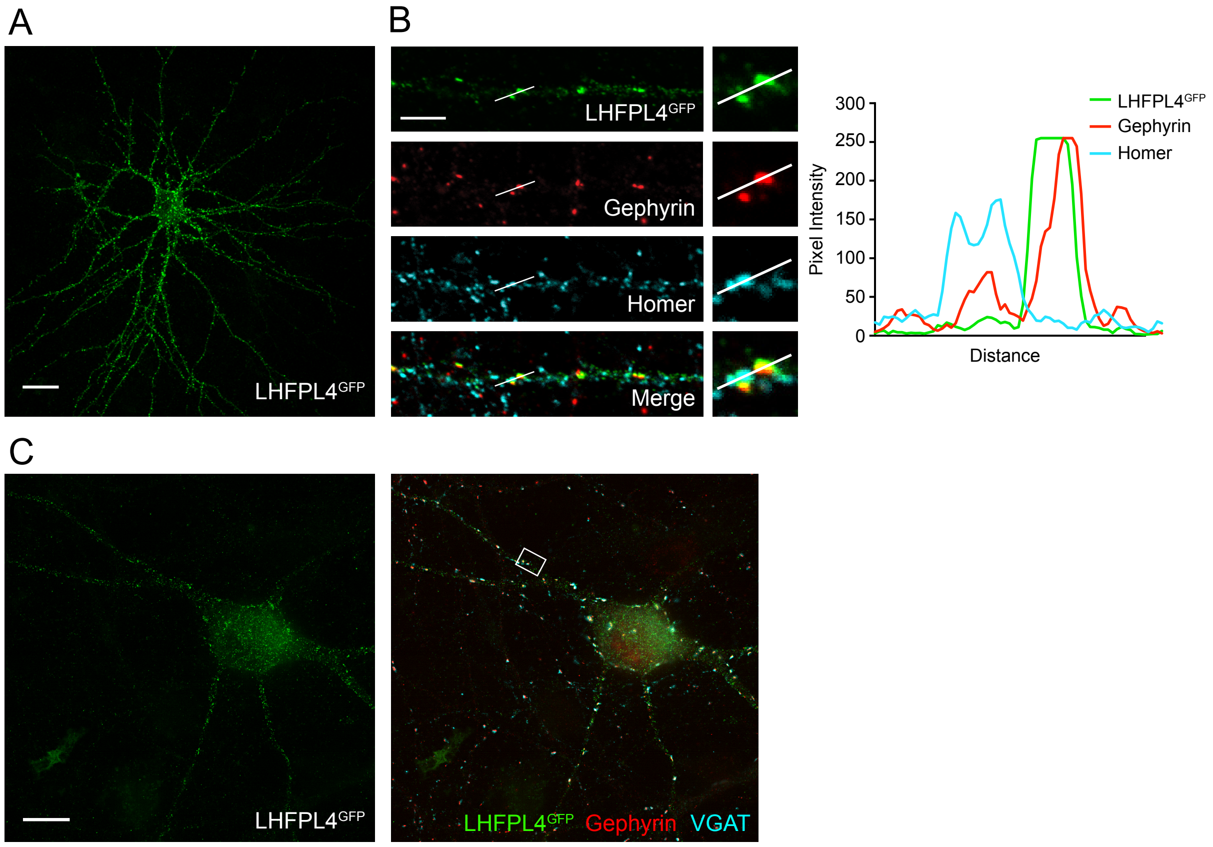

Figure S1, related to Figure 1.
LHFPL4 Specifically Localizes to Inhibitory Synapses.

(A) A maximum intensity projection confocal stack of a DIV16 rat hippocampal neuron transfected with $LHFPL4^{GFP}$. $LHFPL4^{GFP}$ forms distinct clusters along the dendrites and soma. Scale, 20 μm.
(B) Single section confocal zoom images of dissociated hippocampal dendrites transfected with $LHFPL4^{GFP}$ and labeled with antibodies against gephyrin and homer. Graph shows a fluorescence intensity line scan through both gephyrin and homer clusters for each channel as a function of distance. Scale, 5 μm.
(C) SIM images of dissociated hippocampal neurons transfected with $LHFPL4^{GFP}$ and labeled with antibodies against gephyrin and VGAT. Zoom images in Figure 1 are generated from this cell. Scale, 10 μm.

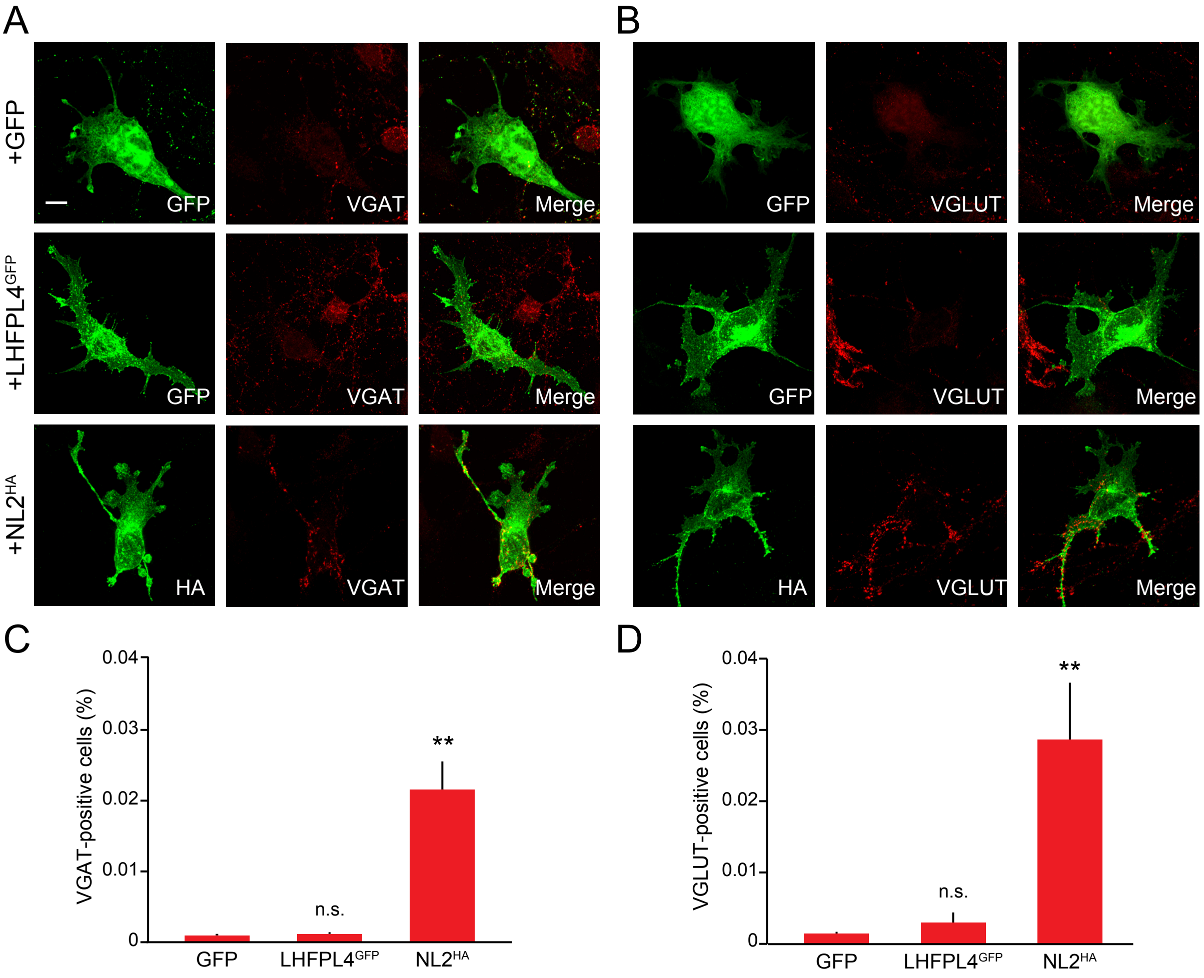

Figure S2, related to Figure 3.
LHFPL4 Does Not Drive the Formation of New Synapses.

(A, B) Representative images of COS-7 cells transfected with GFP, LHFPL4^{GFP} or neuroligin2^{HA} (NL2), co-cultured with dissociated rat hippocampal neurons and labelled with antibodies against (A) VGAT or (B) VGLUT to label inhibitory and excitatory hemi-synapses respectively.
(C, D) Quantification of the proportion of transfected COS-7 cells positive for (C) VGAT or (D) VGLUT. Bars indicate mean and error bars s.e.m. The known synaptogenic molecule NL2 induces the formation of both inhibitory and excitatory hemisynapses whereas LHFPL4 is not different from control. (VGAT hemi-synapses: GFP, 0.10 ± 0.02; LHFPL4^{GFP}: 0.12 ± 0.02; NL2^{HA}, 2.16 ± 0.4; VGLUT hemi-synapses: GFP, 0.14 ± 0.03; LHFPL4^{GFP}: 0.30 ± 0.14; NL2^{HA}, 2.86 ± 0.80). n = 6 coverslips per condition. Kruskal-Wallis one way ANOVA. Asterisks on figure indicate results of Dunn's multiple comparison tests. Scale, 2 μm. **$p < 0.01$.

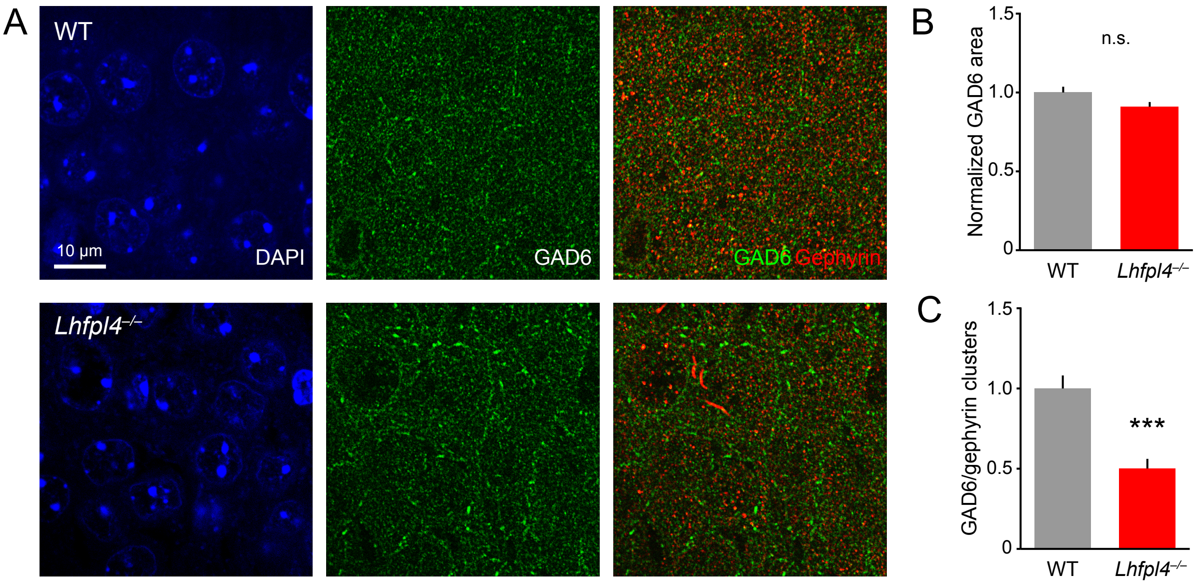

Figure S3, related to Figure 4.
LHFPL4 is Necessary for Gephyrin and $GABA_A$R Clustering but not GAD6 Clustering in Intact Brain.

(A) Confocal images of adult WT and *Lhfpl4*$^{–/–}$ hippocampal brain sections immunolabelled with antibodies to the inhibitory pre and postsynaptic markers GAD6 and gephyrin respectively and co-stained with DAPI to label cell bodies. Merged images are presented so GAD6/gephyrin positive puncta can be identified as orange.
(B) Quantification of normalized total GAD6 cluster area showing no change in clustering in *Lhfpl4*$^{–/–}$ tissue compared to WT (from 1.0 ± 0.04 to 0.9 ± 0.03, n = 16 WT and 12 KO hippocampi; p = 0.066). Bars indicate mean and error bars s.e.m.
(C) Quantification of normalized GAD6/gephyrin positive cluster number showing a significant decrease in colocalised puncta in *Lhfpl4*$^{–/–}$ tissue compared to WT, indicating fewer inhibitory synapses in *Lhfpl4*$^{–/–}$ tissue (from 1.0 ± 0.08 to 0.5 ± 0.05, n = 16 WT and 12 KO hippocampi; p = 0.00005). Bars indicate mean and error bars s.e.m. Background has been subtracted from images so clusters can be easily identified. Scale bar, 10 µm.
***p < 0.001 (Welch t-test).

Supplemental Experimental Procedures

Animals

The *Lhfpl4* (NM_177763) standard knockout mouse line was obtained from Lexicon pharmaceuticals. Animals were maintained under controlled conditions (temperature 20 ± 2 °C; 12 hour light-dark cycle). Food and water were provided *ad libitum*. Standard genotyping was carried out, briefly the DNA was extracted from ear biopsies and PCRs were performed with the following primers: WT forward (a^F): GAGCACTACATGCGGAACTCGC; WT reverse (a^R): CCCGAGCTTTCAACATGAGGG; mutant forward ($laczex^F$): CGATTTGGCTACATGACATCAACC. WT and *Lhfpl4*$^{-/-}$ animals were generated from *Lhfpl4*$^{+/-}$ x *Lhfpl4*$^{+/-}$ crosses. Both male and female mice were used. WT Sprague-Dawley rats were maintained under the same conditions. All procedures for the care and treatment of animals were in accordance with the Animals (Scientific Procedures) Act 1986.

Constructs

Human LHFPL4-GFP, tagged at the N-terminus, was generated by cloning the coding sequence into pDEST53GFP (Invitrogen) using the Gateway Cloning System (Invitrogen). Mouse LHFPL4-turboGFP, tagged at the C-terminus, was purchase from Origene (MG203120). Super-ecliptic pHlourin (SEP)-tagged $GABA_AR$ subunits α2, β3 and γ2 were a gift from Stephen Moss (Tufts University) (Jacob et al., 2005; Tretter et al., 2008). pEGFP-C1 was purchased from Clontech.

Antibodies

The following primary antibodies were used: rabbit anti-LHFPL4 (Sigma, HPA041421, ICC 1:200, WB 1:250), goat anti-LHFPL4 (Santa Cruz, C-17 sc-99499, IP 2 μg), mouse anti-gephyrin (Synaptic Systems, 147011, ICC 1:500), rabbit anti-gephyrin (Synaptic Systems, 147102, IHC 1:250), mouse anti-gephyrin (BD Biosciences, 610585, WB 1:500), rabbit anti-homer (Synaptic Systems, 160002, ICC 1:500), rabbit anti-VGAT (Synaptic Systems, 131003, ICC 1:1000), guinea pig anti-VGLUT (Synaptic Systems, 135304, ICC 1:1000), guinea pig anti-$GABA_AR$ γ2 (Synaptic Systems, 224004, ICC 1:500), rabbit anti-$GABA_AR$ α2 (Synaptic Systems, 224103, ICC 1:500), mouse anti-$GABA_AR$ α1 (Neuromab N95/35, 75-136, WB 1:200), rabbit anti-$GABA_AR$ β3 was raised against MBP-β3-(345-408) and purified on a GST-β3-(345-408) column generated in house, WB 1:100), guinea pig anti-MAP2 (Synaptic Systems, 188004, ICC 1:1000, IHC 1:500), mouse anti-GAD6 was obtained from GAD6 hybridoma cells (ICC 1:200), mouse anti-CAMKIIα (Merck Millipore, 05-532, ICC 1:500), rabbit anti-neuroligin2 (Synaptic Systems, 129202, WB 1:1000, IP 2 μg), mouse anti-β-tubulin (Sigma, T5293, WB 1:1000), rat anti-GFP (Nacalai-Tesque, GF090R, 1:2000), mouse anti-GFP (Neuromab, N86/8 73-131, WB 1:100, IP 2 μg), rabbit anti-turbo GFP (Evrogen, AB513, 1:1000), mouse anti-HA was obtained from 12CA5 hybridoma cells (ICC 1:100). Secondary antibodies were conjugated to Alexa Fluor 488, 568, 647 (1:1000, Molecular Probes) for immunochemistry and anti-mouse or anti-rabbit HRP conjugated for western blotting (Jackson Laboratories, 1:10 000).

Primary Hippocampal Culture

Cultures of cortical and hippocampal neurons were prepared from E16 mouse or E18 Sprague-Dawley rat embryos as described previously (Arancibia-Carcamo et al., 2009; Pathania et al., 2014). Briefly, rat or mouse hippocampi were dissected from embryonic brains in ice-cold HBSS (GIBCO) supplemented with 10mM HEPES and triturated in the presence of 0.25 % trypsin. The dissociated cells were plated on poly-L-lysine coated coverslips in MEM (GIBCO) containing 10 % horse serum, 10 mM sodium pyruvate and 0.6 % glucose at a density of $8.5x10^5$ or $3.5x10^5$ cells/6 cm dish for mouse and rat neurons respectively. The following day the serum-containing medium was replaced with Neurobasal medium (GIBCO) containing 2 % B-27 (GIBCO), 2 mM glutaMAX (GIBCO), 100 μg/mL penicillin and 100 μg/mL streptomycin.

Immunocytochemistry and Immunohistochemistry

Cultured neurons were fixed between DIV14 and DIV16 in 4 % PFA (PBS, 4 % paraformaldehdye, 4 % sucrose, pH 7) for 7 minutes then permeablized for 10 minutes in block solution (PBS, 10 % horse serum, 0.5 % BSA, 0.2 % Triton X-100). Coverslips were incubated with primary antibody diluted in block solution for 1 hour. They were washed in PBS then incubated for another hour with secondary antibody. Finally coverslips were washed and mounted onto glass slides using ProLong Gold antifade reagent (Invitrogen). For surface labeling, block solution was used without detergent.

For immunohistochemistry, adult mouse brains of either sex were fixed in 4 % PFA overnight and cryoprotected in 30 % sucrose/PBS solution overnight before freezing at -80 °C. The brain samples were

embedded in tissue freezing compound (OCT) and 30 µm brain sections were generated using a Cryostat (Bright Instruments). Free floating thin sections were permeablised for 4-5 hours in block solution (PBS, 10 % horse serum, 3 % BSA, 0.5 % Triton X-100, 0.2 M glycine) then incubated with primary antibody diluted in block solution overnight at 4 °C. For mouse primary antibodies, slices were first incubated overnight at 4 °C with mouse Fab fragment (1:25 with block solution; Jackson ImmunoResearch, 115-007-003) to reduce background staining on the mouse tissue. Slices were washed 4-5 times in PBS for 2 hours then incubated for 3-4 hours with secondary antibody at room temperature. The slices were then washed 4-5 times in PBS for 2 hours and mounted onto glass slides using ProLong Gold antifade reagent (Invitrogen). For GABA receptor labeling, mouse brains were snap frozen in isopentane cooled with dry ice and 30 µm brain sections were generated as above. The thin sections were mounted on cold frosted microscope slides at -16 °C. Slices were fixed in 4 % PFA for 90 seconds, then permeablised for 3-4 hours in block solution and incubated with primary and secondary antibodies as above for brain sections. A coverslip was mounted on top using ProLong Gold antifade reagent (Invitrogen). All solutions were prepared on the day of the experiment (Schneider Gasser et al., 2006).

Confocal Image Analysis
Line scans used for protein localization were performed in ImageJ using the PlotProfile function (NIH, Bethesda, MD, USA), pixel intensity was calculated as a function of distance along a manually drawn line and plotted on a graph. Cluster analysis was carried out using Metamorph (Molecular Devices, Sunnyvale, CA, USA). Analysis was carried out on the zoom images and then averaged to give a value per cell or per brain. For cultured cells, the length of dendrite was traced to generate a dendritic region of interest (ROI). This ROI was transferred to all cluster channels. A user-defined threshold was then applied to each cluster channel and regions were generated around the thresholded area within the dendrite ROI. Number of regions and total area of regions per 30 µm of dendrite were quantified as a readout for synaptic clusters. Clusters smaller than 0.1 μm^2 were excluded from the number of regions analysis. Thresholds were set individually for each cluster channel and kept constant across treatment conditions within an experiment. For brain sections labeled with antibody against $GABA_AR$-α2, the above analysis was performed on each whole zoomed region and averaged for each hippocampi. For brain sections labeled with antibodies against gephyrin and GAD6, where discrete clusters are clear in both WT and *Lhfpl4*$^{-/-}$ tissue, the Synapse Counter plugin for ImageJ (NIH, Bethesda, MD, USA) was used. Background subtraction and max filter parameters were set to 10 and 1 respectively. Clusters greater than 0.095 μm^2 and less than 1 μm^2 were considered for total cluster area analysis. For gephyrin aggregates clusters were manually counted.

Live Super-ecliptic pHlourin (SEP) Imaging
DIV14–16 hippocampal cells transfected with $GABA_AR$-$\alpha 2^{SEP}$ were live imaged under perfusion (4 mL/min) in imaging media (125 mM NaCl, 5 mM KCl, 1 mM $MgCl_2$, 2mM $CaCl_2$, 10 mM D-glucose, 10 mM HEPES, adjusted to pH 7.4 with 5 M NaOH) at 37 °C using an Olympus microscope (BX51WI) with a 60X Olympus objective coupled to an EM-CCD camera (Ixon; Andor). Excitation was provided from a metal-halide lamp (X-Cite120, EXFO). Appropriate filters were chosen for SEP-tagged constructs. Movies were recorded at 8.5 Hz. 10 minute movies were acquired where low pH imaging media (pH 5.3) was perfused onto the cells between 3 and 7 minutes. For live cell cluster analysis, single frame images of transfected WT and *Lhflp4*$^{-/-}$ cells were captured. Blind analysis was then carried out where images were manually scored as having clustered or diffuse $GABA_AR$-$\alpha 2^{SEP}$ surface fluorescence.

Co-culture assay
Dissociated rat hippocampal neurons were prepared as described above. At DIV8 COS-7 cells were transfected with the candidate synaptogenic protein. Following 24 hours of expression, COS-7 cells were trypsinised and added to the DIV9 neurons. Approximately 24 hours after co-culture, cells were fixed and stained for appropriate synaptic markers using the immunocytochemistry protocol above. Transfected non-neuronal cells and synaptic labeling were imaged using a Zeiss LSM700 upright confocal microscope. For quantification, both the cell fill and synaptic marker channels were individually thresholded to create two binary images. The images were overlaid and the proportion of the cell pixels that were also positive for a synapse pixel were considered hemi-synapses.

Preparation of Brain Lysates
Adult WT and *Lhflp4*$^{-/-}$ male or female whole brains or cortical regions were homogenized on ice in lysis buffer (50 mM HEPES pH 7.5, 0.5 % Triton-X-100, 150 mM NaCl, 1 mM EDTA, 1 mM PMSF in the presence of antipain, pepstatin and leupeptin) then left to rotate at 4 °C for 2 hours. Membranes were pelleted by ultracentrifugation at 38000 g for 40 minutes at 4 °C. Protein content of the supernatant was assayed by BioRad protein assay.

Co-immunoprecipitation Assays

4 mg of brain lysate or transfected COS-7 cell lysates were transferred to a 1.5 ml microcentrifuge tube and made up to 500 µl with lysis buffer (see above). For GFP/SEP co-immunoprecipitations, GFP/SEP-tagged protein complexes were precipitated from transfected cell lysates with 10 µl of 50 % GFP TRAP bead slurry (Chromtech) for one hour at 4 °C. For co-immunoprecipitations from brain lysate, 2 µg of a primary antibody was added and incubated with the lysate overnight at 4 °C with rotation. Complexes were precipitated with 15 µl of 50 % protein A (for rabbit antibodies) or protein G (for goat antibodies) sepharose bead slurry (Generon) for one hour at 4 °C. All beads were then washed 3 times by centrifugation and resuspension in 1 ml of lysis buffer. Samples were suspended in 3X protein sample buffer and analyzed by SDS-PAGE and western blotting. Briefly, protein samples were separated by SDS-PAGE on 10 % Tris-Glycine gels and blotted onto nitrocellulose membranes (GE Healthcare Bio-Sciences). Membranes were blocked for 1 hour in milk (PBS, 0.05 % Tween, 4 % milk), incubated in primary antibodies diluted with milk overnight at 4 °C before incubation in an appropriate HRP-conjugated secondary antibody for 1 hour at room temperature. The blots were developed with an ECL-Plus detection reagent (GE Healthcare Bio-Sciences). Densitometric analysis was performed in ImageJ (NIH).

Surface Biotinylation Assays

Surface biotinylation assays have been fully described previously (Smith et al., 2014; Twelvetrees et al., 2010). Briefly, cultured cortical cultures were kept at 4 °C and rinsed with PBS (all washing steps were performed in PBS supplemented with 1 mM $CaCl_2$ and 0.5 mM $MgCl_2$) before incubation with 0.5 mg/ml EZ-Link Sulfo-NHS-SS-Biotin (Pierce) for 12 minutes. The surface biotinylation reaction was quenched using three washes of 1 mg/ml BSA in Mg^{2+}/Ca^{2+} supplemented PBS. Cells were washed and solubilized in 360 µl of RIPA buffer (50 mM Tris pH 7.5, 1 mM EDTA, 2 mM EGTA, 150 mM NaCl, 1 % NP40, 0.5 % DOC, 0.1 % SDS, 1 mM PMSF with antipain, pepstatin and leupeptin) for 1 hour at 4 °C. Cell membranes were pelleted at 14 000 rpm for 10 minutes at 4 °C. 60 µl (20 %) was kept to analyze as an input. The remainder was incubated with 50 µl of UltraLink NeutrAvidin 50 % bead slurry (Pierce) for 2 hours at 4 °C. Beads were washed twice with high salt (0.5 M NaCl) RIPA buffer and once with normal (150 mM NaCl) RIPA buffer. Complexes were separated by SDS-PAGE and analyzed by western blotting as above. $GABA_A$Rs were detected with rabbit anti-$GABA_A$R-β3 subunit as primary antibody.

Electrophysiology in Dissociated Cultures

Cultured cells were viewed using an upright microscope (BX50WI; Olympus) and perfused with extracellular solution containing the following (in mM): 145 NaCl, 2.5 KCl, 2 $CaCl_2$, 1 $MgCl_2$, 10 glucose, and 10 HEPES, adjusted to pH 7.3 with NaOH. To block voltage-gated sodium channels, NMDA-, AMPA- and glycine receptors, 0.5 µM TTX, 20 µM D-AP5, 20 µM NBQX and 1 µM strychnine were included. Recording pipettes were pulled (Narishige PC10) from thick-walled borosilicate glass tubing (1.5-mm outer diameter, 0.86-mm inner diameter; Harvard Apparatus), coated with Sylgard (Dow Corning 184) and fire-polished before use. Pipettes were filled with the following internal solution (in mM): 128 CsCl, 10 HEPES, 10 EGTA, 10 tetraethylammonium chloride (TEA-Cl), 2 MgATP, 1 $CaCl_2$, 2 NaCl, 1 QX-314 (Tocris Bioscience), pH 7.4 with CsOH (final resistance ~4–8 MΩ). Currents were recorded at 23–26 °C using an Axopatch 200A amplifier, low-pass filtered at 5 kHz and digitized at 20 kHz using WinEDR (Strathclyde Electrophysiology Software) and ITC-18 interface (InstruTECH). Series resistance and input capacitance were read directly from the amplifier settings used to minimize the current responses to 5 mV hyperpolarizing voltage steps. Series resistance was typically compensated by 60–80% and data were discarded if the series resistance varied by >20%. The "phasic charge transfer" due to mIPSCs was calculated using an automated procedure (written in IGOR Pro 6.35; WaveMetrics) that avoided subjective decisions regarding detection or selection of individual synaptic currents. The record was split into segments (typically 1s) and for each segment an all-point amplitude histogram was generated and fit with a single-sided Gaussian to the most-positive current values. The position of the peak of the histogram was taken as the baseline current for that segment and subtracted from the record. The integral of the subtracted current provided the charge carried by the synaptic events. The total charge was divided by the recording time to give a measure of phasic charge transfer per second.

Acute Hippocampal Slice Electrophysiology

To prepare acute brain slices, male and female mice (P30–45) were deeply anesthetized with isoflurane and decapitated. The brain was removed and submerged in ice-cold slicing solution containing (in mM): 85 NaCl, 2.5 KCl, 7 $MgCl_2$, 1.25 NaH_2PO4, 0.5 $CaCl_2$, 25 $NaHCO_3$, 25 glucose, and 75 sucrose, saturated with 95% O_2/5% CO_2, pH 7.4. Coronal hippocampal slices (300 µm thick) were prepared using a vibratome (Campden 7000smz). Slices were maintained at 32.5 °C for 30 min and then at room temperature for at

least 30 min to recover. During this period the slicing solution in the holding chamber was replaced (~5 ml min^{-1}) with a recording solution containing (in mM): 125 NaCl, 2.5 KCl, 1 $MgCl_2$, 1.25 NaH_2PO_4, 2 $CaCl_2$, 38 glucose, and 26 $NaHCO_3$ saturated with 95% O_2/5% CO_2, pH 7.4. For recording, slices were placed in a submerged chamber on the stage of an upright microscope (Scientifica SliceScope). Phasic charge transfer was analyzed as described above. Spontaneous currents were detected using a scaled template algorithm based on rising and decaying exponentials (Clements and Bekkers, 1997) (NeuroMatic 2.9, http://www.neuromatic. thinkrandom.com/). When analyzing current frequency, all events were counted, irrespective of overlapping decays. For kinetic analysis, only events with a monotonic rise and uncontaminated decay were included; they were aligned on their rising phase before averaging. The decay of averaged fast mIPSCs was described by the sum of two exponentials, from which the weighted time constant of decay (τ_w, decay) was calculated as the sum of the time constants weighted by their fractional amplitudes. Slow mIPSCs were detected using a template with a rise of 10 ms and a decay of 50 ms. To reduce error when measuring the rise and decay time of these slow currents, individual events were fitted with an empirical equation:

$$f(\mathrm{x}) = \left(1 * \exp\left(-\frac{\mathrm{x}-\mathrm{X0}}{\tau_{\mathrm{rise}}}\right)\right)^N * \left(A_{\mathrm{f}} * \exp\left(-\frac{\mathrm{x}-\mathrm{X0}}{\tau_{\mathrm{f}}}\right) + A_{\mathrm{s}} * \exp\left(-\frac{\mathrm{x}-\mathrm{X0}}{\tau_{\mathrm{s}}}\right)\right)$$

and 10–90% rise time and 63% decay time measures were taken from the fit waveform (Bekkers and Clements, 1999; Bekkers and Stevens, 1996).

For mIPSCs and tonic current measurement, recording pipettes were filled with an internal solution containing (in mM): 128 CsCl, 10 EGTA-Cs, 10 HEPES, 2 MgATP, 1 $CaCl_2$, 2 NaCl, 1 QX-314 (Tocris Bioscience), and 5 TEA-Cl, adjusted to pH 7.3 with CsOH. In some cases this solution also contained 0.2% biocytin (Molecular Probes). D-AP5 (20 μM, Abcam) and NBQX (10 μM, Abcam) were added to the external solution to block NMDARs and AMPARs. All mIPSCs were blocked by bicuculline (20 μM, Tocris) or gabazine (20 μM, Abcam). To record mEPSCs, NBQX was omitted and bicuculline (20 μM) or gabazine (20 μM) were added to the external solution. As the mEPSC frequency appeared low under these conditions, all data were obtained under conditions designed to increase mEPSC frequency and thus maximize the number of recordings with an acceptable number of events. Thus, we increased extracellular K^+ (additional 5 mM KCl), made recordings at an elevated temperature (32.5 °C), and used an internal solution containing (in mM) 135 Cs-gluconate, 10 HEPES, 10 Na-phosphocreatine, 4 MgATP, 0.4 NaGTP, 2 QX-314, and 10 TEA-Cl, adjusted to pH 7.3 with CsOH.

After recording, slices were fixed overnight at 4 °C in a solution containing 4% PFA. Slices were then washed in PBS 1x and incubated for 3 hours at room temperature in a solution containing 1:1000 triton X-100 with1:1000 strepavidin-555 (Molecular Probes). Slices were then washed three times in PBS, incubated for 10 minutes at room temperature with DAPI (1:1000) and mounted using anti-Fade gold (Invitrogen P36930). Confocal images were acquired on a Leica TCS SPE upright confocal microscope using a 20X oil objective (NA: 0.6) and digitally captured using LAS software.

Supplemental References

Arancibia-Carcamo, I.L., Yuen, E.Y., Muir, J., Lumb, M.J., Michels, G., Saliba, R.S., Smart, T.G., Yan, Z., Kittler, J.T., and Moss, S.J. (2009). Ubiquitin-dependent lysosomal targeting of $GABA_A$ receptors regulates neuronal inhibition. Proc. Natl. Acad. Sci. *106*, 17552–17557.

Bekkers, J.M., and Clements, J.D. (1999). Quantal amplitude and quantal variance of strontium-induced asynchronous EPSCs in rat dentate granule neurons. J. Physiol. *516*, 227–248.

Bekkers, J.M., and Stevens, C.F. (1996). Cable properties of cultured hippocampal neurons determined from sucrose-evoked miniature EPSCs. J. Neurophysiol. *75*, 1250–1255.

Jacob, T.C., Bogdanov, Y.D., Magnus, C., Saliba, R.S., Kittler, J.T., Haydon, P.G., and Moss, S.J. (2005). Gephyrin regulates the cell surface dynamics of synaptic $GABA_A$ receptors. J. Neurosci. *25*, 10469–10478.

Pathania, M., Davenport, E.C., Muir, J., Sheehan, D.F., López-Doménech, G., and Kittler, J.T. (2014). The autism and schizophrenia associated gene CYFIP1 is critical for the maintenance of dendritic complexity and the stabilization of mature spines. Transl. Psychiatry *4*, e374.

Schneider Gasser, E.M., Straub, C.J., Panzanelli, P., Weinmann, O., Sassoè-Pognetto, M., and Fritschy, J.-M. (2006). Immunofluorescence in brain sections: simultaneous detection of presynaptic and postsynaptic proteins in identified neurons. Nat. Protoc. *1*, 1887–1897.

Smith, K.R., Davenport, E.C., Wei, J., Li, X., Pathania, M., Vaccaro, V., Yan, Z., and Kittler, J.T. (2014). GIT1 and βPIX are essential for $GABA_A$ receptor synaptic stability and inhibitory neurotransmission. Cell Rep. *9*, 298–310.

Tretter, V., Jacob, T.C., Mukherjee, J., Fritschy, J.-M., Pangalos, M.N., and Moss, S.J. (2008). The clustering of GABA(A) receptor subtypes at inhibitory synapses is facilitated via the direct binding of receptor alpha 2 subunits to gephyrin. J. Neurosci. *28*, 1356–1365.

Twelvetrees, A.E., Yuen, E.Y., Arancibia-Carcamo, I.L., MacAskill, A.F., Rostaing, P., Lumb, M.J., Humbert, S., Triller, A., Saudou, F., Yan, Z., et al. (2010). Delivery of $GABA_A$Rs to synapses is mediated by HAP1-KIF5 and disrupted by mutant huntingtin. Neuron *65*, 53–65.
