# Supplementary material for: An Essential Role for the Tetraspanin LHFPL4 in the Cell-Type-Specific Targeting and Clustering of Synaptic GABAA Receptors
Source: Cell Rep. 2017 Oct 3;21(1):70–83. doi: 10.1016/j.celrep.2017.09.025 (PMC5640807; doi:10.1016/j.celrep.2017.09.025)
Supplement: Document S2. Article plus Supplemental Information [file mmc2.pdf]

Cell Reports

Article

An Essential Role for the Tetraspanin LHFPL4 in the Cell-Type-Specific Targeting and Clustering of Synaptic $GABA_A$ Receptors

Graphical Abstract

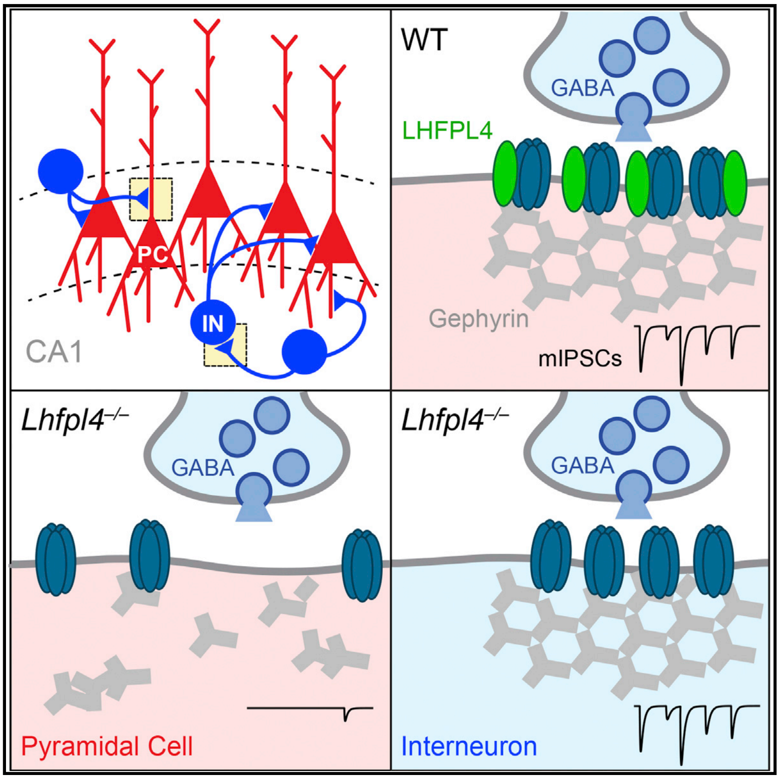

Highlights

- LHFPL4 is a tetraspanin enriched at inhibitory synapses that complexes with $GABA_A$Rs
- LHFPL4 is important for $GABA_A$R clustering both in vitro and in vivo
- LHFPL4 is required for the surface clustering but not the trafficking of $GABA_A$Rs
- GABAergic synaptic inputs on CA1 pyramidal neurons, but not interneurons, require LHFPL4

Authors

Elizabeth C. Davenport, Valentina Pendolino, Georgina Kontou, ..., Guillermo López-Doménech, Mark Farrant, Josef T. Kittler

Correspondence

m.farrant@ucl.ac.uk (M.F.), j.kittler@ucl.ac.uk (J.T.K.)

In Brief

Davenport et al. identify LHFPL4 as a transmembrane protein that interacts with $GABA_A$Rs and is essential for their synaptic clustering. Deletion of LHFPL4 results in dramatic cell-type-specific deficits in inhibitory synaptic transmission.

Davenport et al., 2017, Cell Reports *21*, 70–83
October 3, 2017 © 2017 The Authors.
http://dx.doi.org/10.1016/j.celrep.2017.09.025

OPEN ACCESS
CellPress

Cell Reports
Article

An Essential Role for the Tetraspanin LHFPL4 in the Cell-Type-Specific Targeting and Clustering of Synaptic $GABA_A$ Receptors

Elizabeth C. Davenport,[1,2] Valentina Pendolino,[1] Georgina Kontou,[1] Thomas P. McGee,[1] David F. Sheehan,[1] Guillermo López-Doménech,[1] Mark Farrant,[1,*] and Josef T. Kittler[1,3,*]
[1]Department of Neuroscience, Physiology and Pharmacology, University College London, Gower Street, London WC1E 6BT, UK
[2]Present address: Centre for Integrative Physiology, University of Edinburgh, Hugh Robson Building, George Square, Edinburgh EH8 9XD, Scotland, UK
[3]Lead Contact
*Correspondence: m.farrant@ucl.ac.uk (M.F.), j.kittler@ucl.ac.uk (J.T.K.)
http://dx.doi.org/10.1016/j.celrep.2017.09.025

SUMMARY

Inhibitory synaptic transmission requires the targeting and stabilization of $GABA_A$ receptors ($GABA_A$Rs) at synapses. The mechanisms responsible remain poorly understood, and roles for transmembrane accessory proteins have not been established. Using molecular, imaging, and electrophysiological approaches, we identify the tetraspanin LHFPL4 as a critical regulator of postsynaptic $GABA_A$R clustering in hippocampal pyramidal neurons. LHFPL4 interacts tightly with $GABA_A$R subunits and is selectively enriched at inhibitory synapses. In LHFPL4 knockout mice, there is a dramatic cell-type-specific reduction in $GABA_A$R and gephyrin clusters and an accumulation of large intracellular gephyrin aggregates in vivo. While $GABA_A$Rs are still trafficked to the neuronal surface in pyramidal neurons, they are no longer localized at synapses, resulting in a profound loss of fast inhibitory postsynaptic currents. Hippocampal interneuron currents remain unaffected. Our results establish LHFPL4 as a synapse-specific tetraspanin essential for inhibitory synapse function and provide fresh insights into the molecular make-up of inhibitory synapses.

INTRODUCTION

Synaptic inhibition mediated by $GABA_A$ receptors ($GABA_A$Rs) regulates the balance of excitation and inhibition in the brain and, thus, plays a critical role in information processing. The stabilization of synaptic $GABA_A$Rs opposite GABA-releasing presynaptic terminals is crucial for efficient synaptic inhibition, appropriate regulation of circuit excitability, and animal behavior (Charych et al., 2009; Crestani et al., 1999; Luscher et al., 2011a; Papadopoulos et al., 2007). Changing the number of postsynaptic $GABA_A$Rs can rapidly control the strength of inhibitory synapses. This is achieved by the trafficking of receptors to, and their removal from, the plasma membrane and by their surface lateral diffusion into and out of synaptically stabilized clusters (Bannai et al., 2009; Luscher et al., 2011b; Muir et al., 2010; Twelvetrees et al., 2010). At the synapse, $GABA_A$R clustering and anchoring are mediated by a complex inhibitory postsynaptic density, the major constituent of which is the hexameric scaffold gephyrin (Tyagarajan and Fritschy, 2014). However, in the absence of gephyrin, subsets of inhibitory synapses remain (Essrich et al., 1998; Kneussel et al., 1999; O'Sullivan et al., 2009), and genetic deletion of gephyrin in the CNS has an unexpectedly subtle effect on inhibitory synaptic transmission (Lévi et al., 2004). These observations suggest the existence of as-yet-unidentified molecules important for stabilizing $GABA_A$Rs at synapses.

Several membrane-spanning adhesion molecules, including neuroligin2, slitrk3, and calsyntenin3 (Pettem et al., 2013; Pouloupoulos et al., 2009; Takahashi et al., 2012), contribute to the formation and stabilization of GABAergic synapses. However, virtually nothing is known regarding roles for proteins that might function as transmembrane $GABA_A$R accessory proteins. In the case of ionotropic glutamate receptors, various membrane-spanning receptor-associated proteins have emerged as key regulators of receptor trafficking, synaptic targeting, and receptor function. These include transmembrane α-amino-3-hydroxy-5-methyl-4-isoxazolepropionic acid receptor (AMPAR) regulatory proteins (TARPs; γ-2, -3, -4, -5, -7, and -8), cornichons (CNIH1 and CNIH2), neuropilin and tolloid-like proteins (NETO1 and NETO2), and GSG1L (reviewed in Copits and Swanson, 2012; Haering et al., 2014; Jackson and Nicoll, 2011). Notably, TARPs and GSG1L are members of the tetraspanin superfamily of transmembrane proteins and have been shown to associate with and regulate AMPARs. Whether undiscovered tetraspanin-like molecules act to similarly coordinate $GABA_A$R trafficking, synaptic stability, and function remains unknown.

Here, using biochemical, imaging, mouse transgenic, and electrophysiological approaches, we demonstrate a critical role for the previously uncharacterized tetraspanin LHFPL4 (Lipoma HMGIC Fusion Partner-Like 4) in driving the surface clustering of $GABA_A$Rs at inhibitory synapses. LHFPL4 is exquisitely targeted to inhibitory synapses and forms high-affinity interactions with $GABA_A$R subunits. In the absence of LHFPL4, $GABA_A$Rs can still reach the cell surface but are no longer synaptically anchored, leading to a loss of inhibitory postsynaptic currents. We find that LHFPL4 acts in a cell-type-specific manner

 © 2017 The Authors.
This is an open access article under the CC BY license (http://creativecommons.org/licenses/by/4.0/).

within the hippocampus, with excitatory pyramidal cells but not inhibitory interneurons affected by its deletion. Our identification of a new machinery for synaptic targeting of $GABA_AR$s opens up new avenues for understanding the construction and regulation of inhibitory synapses in the brain.

RESULTS

LHFPL4 Is Targeted to Inhibitory Synapses and Interacts with $GABA_AR$s

Although recent mass spectrometry studies have identified a number of candidate $GABA_AR$-interacting proteins (Heller et al., 2012; Nakamura et al., 2016), the biochemical validation and functional role of the majority of these putative partners remain undetermined. We noted with interest the identification of LHFPL4, a predicted member of the tetraspanin superfamily of transmembrane proteins of unknown function. We initially tested the ability of LHFPL4 to interact with $GABA_AR$ subunits. Mouse turbo-GFP-tagged LHFPL4 ($mLHFPL4^{tGFP}$) could be readily co-immunoprecipitated with $GABA_AR$ subunits (α2, β3, and γ2) from lysates of co-transfected COS-7 cells, suggesting that other neuronally expressed synaptic proteins, such as gephyrin, are not essential for the interaction. In the reverse experiment, GFP-tagged human LHFPL4 ($hLHFPL4^{GFP}$) could also readily co-immunoprecipitate the $GABA_AR$-α1 subunit from COS-7 cell lysates (Figures 1A and 1B).

To determine whether LHFPL4 was present at synaptic sites, we examined the subcellular localization of $LHFPL4^{GFP}$ in cultured rat hippocampal neurons. Using laser scanning confocal microscopy (LSCM), $LHFPL4^{GFP}$ was observed to form discrete membrane clusters on the soma and throughout the dendrites. These robustly colocalized with the inhibitory postsynaptic marker gephyrin opposite vesicular GABA transporter (VGAT)-labeled inhibitory presynaptic terminals. Line scans through $LHFPL4^{GFP}$ clusters revealed peak fluorescence essentially overlapping with gephyrin and adjacent to the peak of VGAT fluorescence (Figures 1C, 1D, and S1A). Co-labeling of $LHFPL4^{GFP}$ with gephyrin and the excitatory synaptic postsynaptic density (PSD) protein homer revealed that $LHFPL4^{GFP}$ clusters were 6-fold more enriched at inhibitory compared to excitatory synapses (Figures 1E and S1B). We next explored the postsynaptic distribution of LHFPL4 at inhibitory synaptic sites, using structured illumination microscopy (SIM) to overcome the resolution limit of conventional fluorescence microscopy. SIM imaging of $LHFPL4^{GFP}$ co-labeled with antibodies to gephyrin and VGAT revealed $LHFPL4^{GFP}$ to form groups of nano-clusters overlaying gephyrin puncta (Figures 1F and S1C), further supporting an inhibitory postsynaptic localization for LHFPL4. Importantly, immunolabeling with an LHFPL4-specific antibody demonstrated that endogenous LHFPL4 is selectively enriched at gephyrin-labeled inhibitory synapses (Figure 1G). Together, these data indicate that LHFPL4 intimately associates with $GABA_AR$s and is specifically enriched at inhibitory postsynaptic domains.

LHFPL4 Is Essential for the Clustering of $GABA_AR$s but Not Their Surface Delivery

To investigate the consequences of LHFPL4 loss on the targeting of $GABA_AR$s to inhibitory synapses, we characterized neurons from a constitutive LHFPL4 knockout (KO) mouse (*Lhfpl4*$^{-/-}$) (Figures 2A–2C). These animals were viable until adulthood, were fertile, and showed no obvious behavioral differences from wild-type (WT) animals. A band at the expected molecular weight for LHFPL4 (27 kDa) was detected from WT, but not *Lhfpl4*$^{-/-}$, brain lysate by western blotting, and a further strong LHFPL4-specific band was detected at ~17 kDa, which may represent a second isoform or a cleaved product (Figure 2C). Hippocampal neurons from WT and *Lhfpl4*$^{-/-}$ mice were transfected with GFP to reveal cell morphology and were fixed and labeled with an antibody specific to a surface epitope on the $GABA_AR$-γ2 subunit before being permeabilized and labeled with antibodies against gephyrin and VGAT (Smith et al., 2014). Quantification of LSCM images revealed a dramatic loss of both gephyrin and $GABA_AR$-γ2 clustering and a marked decrease in VGAT-positive clusters co-labeled for gephyrin in *Lhfpl4*$^{-/-}$ neurons compared to WT (Figures 2D–2G). By contrast, clustering of VGAT alone and homer were unchanged in *Lhfpl4*$^{-/-}$ neurons, indicating that inhibitory presynaptic terminals and excitatory synapses were unaffected (Figures 2H–2J). Importantly, the loss of gephyrin clustering in *Lhfpl4*$^{-/-}$ neurons could be robustly rescued upon overexpression of $LHFPL4^{GFP}$, while $LHFPL4^{GFP}$ had no effect on synaptic number or area when overexpressed on the WT background (Figures 2K and 2L).

To determine whether the loss of synaptic $GABA_AR$ clusters in *Lhfpl4*$^{-/-}$ neurons was due to a disruption in $GABA_AR$ surface trafficking (Twelvetrees et al., 2010) or to altered synaptic targeting of surface-trafficked receptors (Muir et al., 2010), we next performed live imaging of a super-ecliptic pHluorin (SEP)-tagged $GABA_AR$ subunit (Muir and Kittler, 2014; Pathania et al., 2014). We used $GABA_AR$-$\alpha2^{SEP}$, as this construct is easily expressed, assembles with endogenous $GABA_AR$ subunits, and demonstrates the expected pattern of surface fluorescence (Muir et al., 2010). Thus, when transfected into WT neurons, $GABA_AR$-$\alpha2^{SEP}$ formed bright fluorescent clusters along the dendrites with lower levels of diffuse labeling, consistent with previous reports (Eckel et al., 2015; Muir et al., 2010; Tretter et al., 2008). By contrast, in the majority of transfected *Lhfpl4*$^{-/-}$ cells, $GABA_AR$-$\alpha2^{SEP}$ clustering was absent, with only diffuse fluorescence present throughout the soma and dendrites (Figures 3A–3C). Blind scoring of the clustered or diffuse nature of $GABA_AR$-$\alpha2^{SEP}$ fluorescence revealed significant loss of clustering in neurons cultured from *Lhfpl4*$^{-/-}$ mice (Figure 3D). Importantly, both in WT and *Lhfpl4*$^{-/-}$ neurons, the $GABA_AR$-$\alpha2^{SEP}$ fluorescence was rapidly and reversibly eclipsed by transient exposure to extracellular buffer of low pH (Figures 3A and 3B), confirming that the fluorescent signal originated from cell-surface receptors. Furthermore, biotinylation experiments showed that surface levels of endogenous $GABA_AR$s were unchanged in *Lhfpl4*$^{-/-}$ neurons compared to WT (Figures 3E and 3F). These data suggest that loss of LHFPL4 does not interfere with the trafficking of $GABA_AR$s to the cell surface but, in a majority of cells, disrupts their tethering in synaptic clusters.

LHFPL4 Is Not Synaptogenic

Many inhibitory postsynaptic transmembrane molecules, when overexpressed in non-neuronal cells maintained with dissociated neurons, can induce the formation of hemi-synapses by

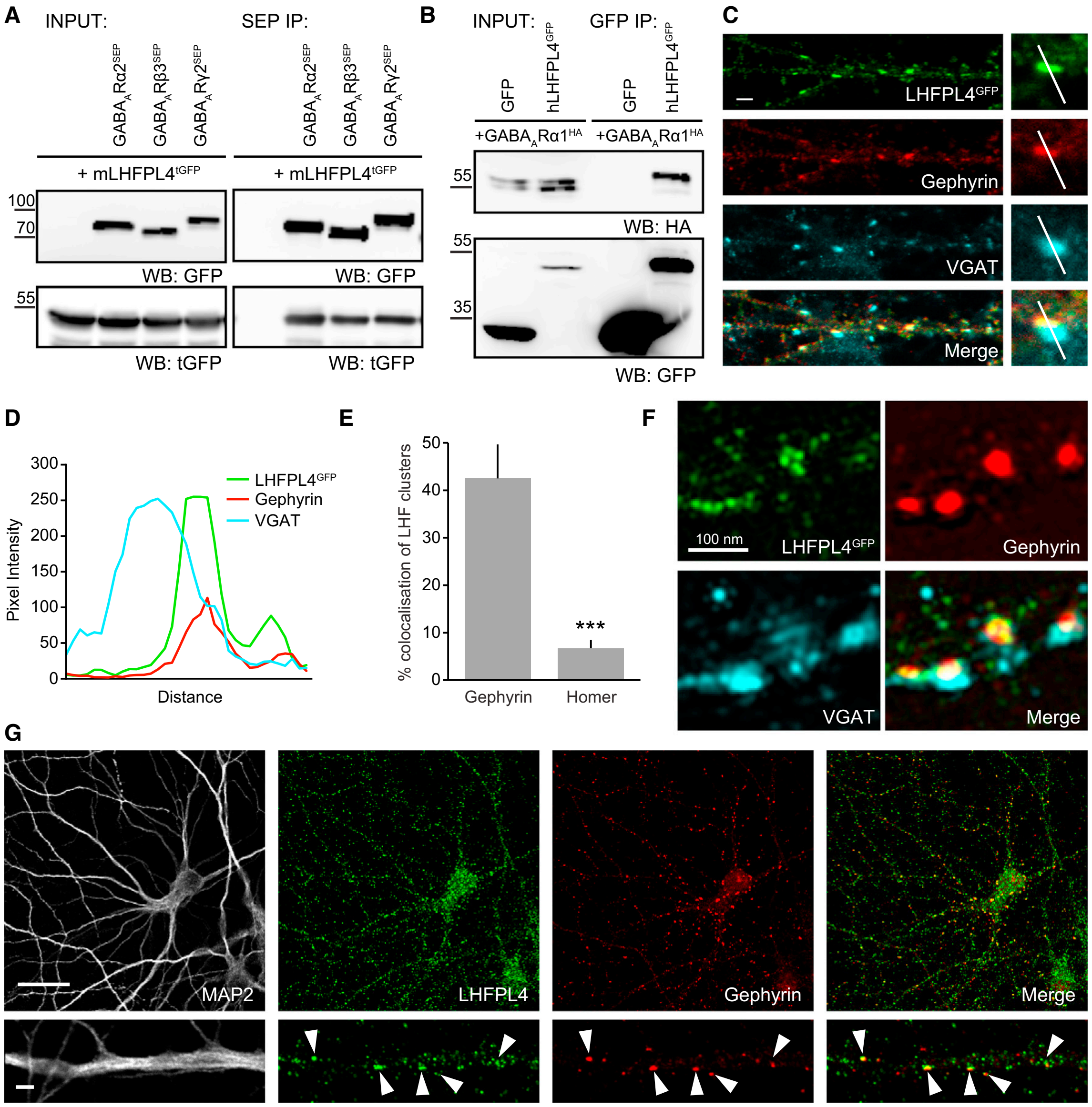

Figure 1. LHFPL4 Specifically Localizes to Inhibitory Synapses

(A) Co-immunoprecipitation (coIP) of mouse LHFPL4-turboGFP (tGFP) with super-ecliptic-pHlourin (SEP)-tagged α2, β3, and γ2 $GABA_AR$ subunits from transfected COS7 cells (WB, western blot; IP, immunoprecipitation).

(B) CoIP of human LHFPL4-GFP (hLHFPL4) with hemagglutinin (HA)-tagged $GABA_AR$-α1 from transfected COS7 cells (WB, western blot; IP, immunoprecipitation).

(C) Confocal images of dissociated rat hippocampal neurons transfected with $LHFPL4^{GFP}$ and labeled with antibodies against gephyrin and VGAT reveal a close association between $LHFPL4^{GFP}$ and gephyrin opposed to VGAT. Scale, 2 μm.

(D) Graph shows a fluorescence intensity line scan through a synaptic cluster (white lines in C) for each channel as a function of distance.

(E) Quantification of $LHFPL4^{GFP}$ clusters colocalized with gephyrin compared to homer puncta, generated from transfected hippocampal neurons and labeled with antibodies against gephyrin and homer (gephyrin: 42.5% ± 4.4%; homer: 6.7% ± 1.4%; n = 12 cells from 2 independent preparations; $p < 0.001$, Welch t test). Error bars indicate mean ± SEM. ***$p < 0.001$.

(F) Single-section SIM zoom images of hippocampal neurons transfected with $LHFPL4^{GFP}$ and labeled with antibodies against gephyrin and VGAT.

(G) Confocal images of hippocampal neurons labeled with antibodies to endogenous LHFPL4, MAP2, and gephyrin reveal LHFPL4 overlaps with inhibitory synapses (white arrowheads). Scale, 20 μm (top) and 2 μm (bottom).

See also Figure S1.

aggregating presynaptic proteins at the point of contact between the two cell types (Fuchs et al., 2013; Scheiffele et al., 2000; Takahashi et al., 2012). To test whether LHFPL4 shared these synaptogenic properties, we co-cultured COS-7 cells (overexpressing recombinant putative synaptogenic transmembrane proteins) with dissociated WT rat hippocampal neurons. After

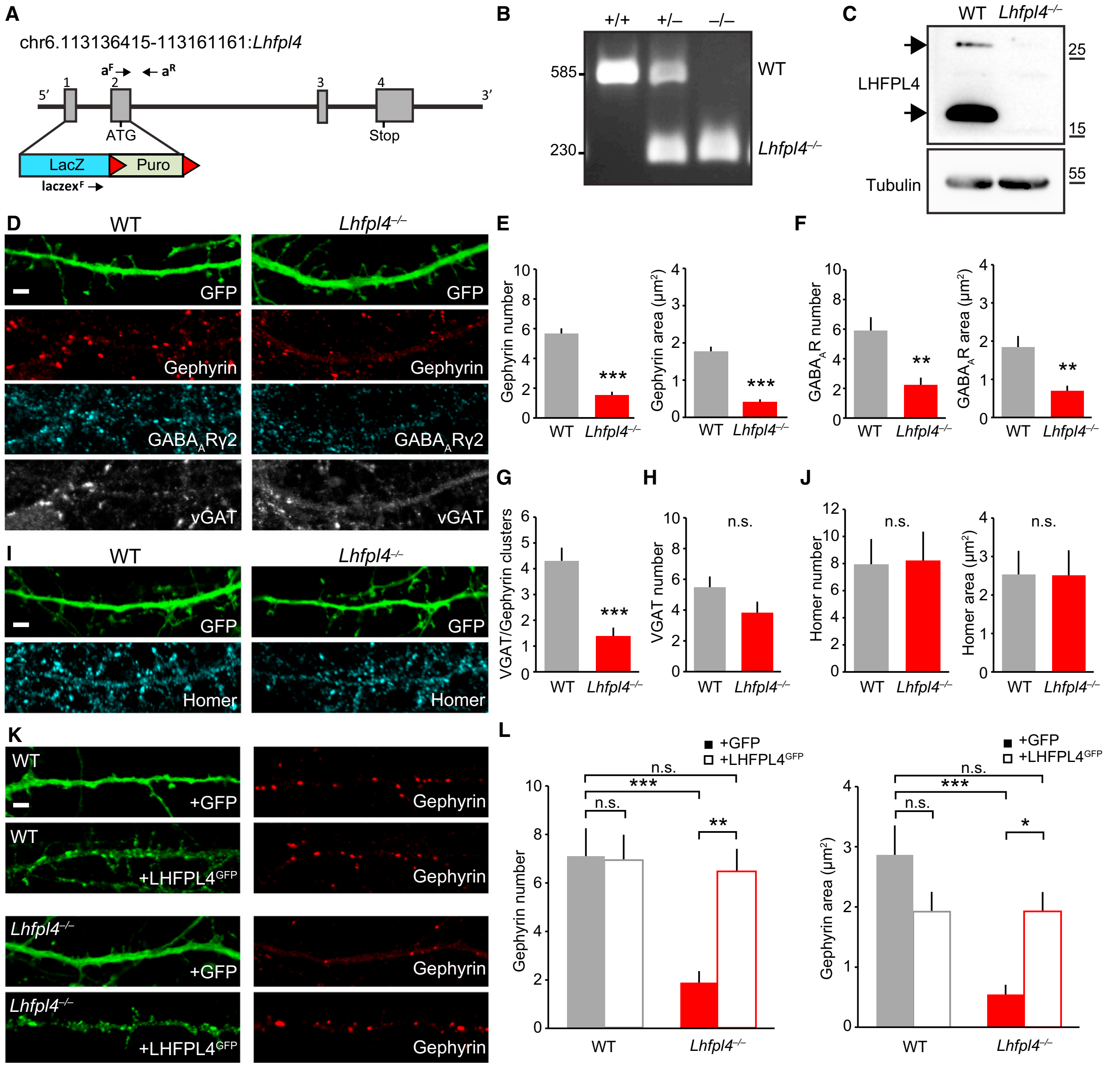

Figure 2. Loss of Inhibitory Synapse Stability and GABA$_A$R Clustering in *Lhfpl4*$^{-/-}$ Neurons

(A) Schematic of the LHFPL4 knockout genetic strategy showing the genotyping primer sites.

(B) Genotyping results from WT (+/+), heterozygous (+/−) and homozygous (−/−) animals. The product of primers a^F and a^R generates a 585-bp band from the WT allele, and the product of laczex^F and a^R generates a 230-bp band from the *Lhfpl4*$^{-/-}$ allele.

(C) Western blotting of LHFPL4 and tubulin from WT and *Lhfpl4*$^{-/-}$ mouse brain lysates. Arrowheads indicate the two LHFPL4-specific bands detected.

(D and I) Confocal images of dissociated DIV14 (14 days in vitro) WT or *Lhfpl4*$^{-/-}$ hippocampal neurons transfected with GFP and labeled with antibodies to (D) gephyrin, GABA$_A$R-γ2, and VGAT or (I) homer.

(E–H and J) Quantification of (E) gephryin, (F) GABA$_A$R-γ2, (G) VGAT/gephyrin, (H) VGAT and (J) homer synaptic clusters. Cluster number and total immunolabeled area were significantly reduced in *Lhfpl4*$^{-/-}$ neurons, compared to WT neurons, when labeled for gephyrin (cluster number: from 5.7 ± 0.4 to 1.5 ± 0.2; area: from 1.8 μm^2 ± 0.1 μm^2 to 0.4 μm^2 ± 0.1 μm^2; 484/166 WT/*Lhfpl4*$^{-/-}$ clusters; both p < 0.0001) and GABA$_A$R-γ2 (cluster number: from 5.9 ± 0.9 to 2.2 ± 0.5; area: from 1.8 μm^2 ± 0.3 μm^2 to 0.7 μm^2 ± 0.1 μm^2; 636/327 WT/*Lhfpl4*$^{-/-}$ clusters; p = 0.0011 and 0.0013, respectively). VGAT/gephyrin-positive clusters were significantly reduced in *Lhfpl4*$^{-/-}$ neurons (from 4.3 ± 0.5 to 1.4 ± 0.3; 294/95 WT/*Lhfpl4*$^{-/-}$ clusters; p < 0.0001). VGAT cluster number did not significantly change (from 5.5 ± 0.7 to 3.8 ± 0.7; 531/403 WT/*Lhfpl4*$^{-/-}$ clusters; p = 0.103). In each case, n = 23 WT and 23 *Lhfpl4*$^{-/-}$ cells from 3 independent preparations. For WT and *Lhfpl4*$^{-/-}$ clusters, there was no significant change in homer cluster number (8.0 ± 1.9 and 8.2 ± 2.1, respectively; 333/345 WT/*Lhfpl4*$^{-/-}$ clusters) or area (2.5 μm^2 ± 0.6 μm^2 and 2.5 μm^2 ± 0.7 μm^2, respectively; n = 14 WT and KO cells from 3 independent preparations; p = 0.92 and 0.98, respectively). All used Welch t tests.

(legend continued on next page)

OPEN
ACCESS
CellPress

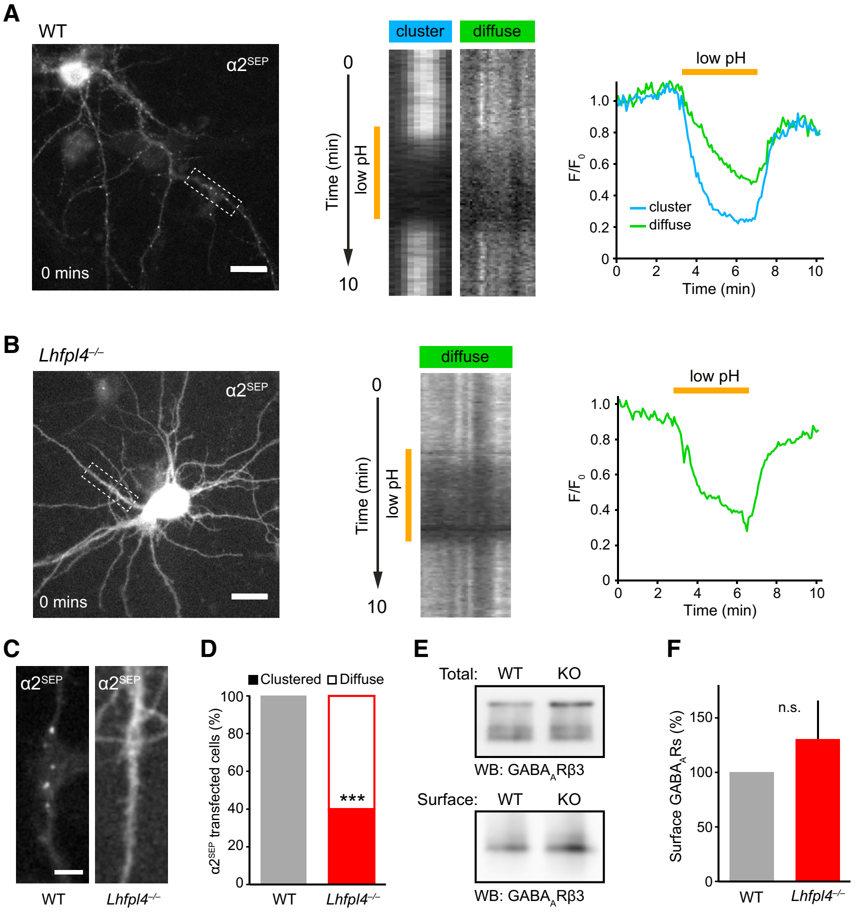

Figure 3. LHFPL4 Is Essential for GABA$_A$R Clustering but Not Their Surface Delivery

(A and B) Bright-field images of (A) WT and (B) *Lhfpl4*$^{-/-}$ hippocampal neurons transfected with super-ecliptic pHlourin-tagged GABA$_A$R-α2 ($\alpha 2^{SEP}$). Kymograph and graph representations of 10-min movies showing pH-dependent $\alpha 2^{SEP}$ clusters or diffuse fluorescence signal from dendrite boxed in image. Fluorescence from dendrites is eclipsed on a transient switch to low-pH imaging solution and returns on a switch back to pH 7.4, indicating that the $\alpha 2^{SEP}$ fluorescence signal is from surface receptors. Scale, 20 μm.

(C) Dendritic zooms of bright-field images of WT and *Lhfpl4*$^{-/-}$ hippocampal dendrites transfected with GABA$_A$R-$\alpha 2^{SEP}$. GABA$_A$R-$\alpha 2^{SEP}$-containing receptors do not cluster on the surface of *Lhfpl4*$^{-/-}$ neurons. Scale, 5 μm.

(D) Quantification of GABA$_A$R-$\alpha 2^{SEP}$ surface fluorescence as clustered or diffuse (n = 50–53 cells from 3 independent preparations; $p < 0.0001$, Fisher's exact test).

(E) Surface biotinylations of WT and *Lhfpl4*$^{-/-}$ neurons analyzed by western blotting with anti-GABA$_A$R-β3.

(F) Densiometric quantification showing no significant change in surface GABA$_A$R-β3 normalized to total levels (100% and 130.4% ± 35.4%; n = 11 experiments; p = 0.41, Welch t test).

Error bars indicate mean ± SEM. *p < 0.05; **p < 0.01; ***p < 0.001.

See also Figure S2.

24 hr, the cells were fixed and labeled with antibodies against VGAT and vesicular glutamate transporter (VGLUT) to identify inhibitory and excitatory hemi-synapses, respectively. Consistent with previous reports (Chih et al., 2006; Scheiffele et al., 2000), COS-7 cells overexpressing neuroligin2 induced the formation of both inhibitory and excitatory hemi-synapses (Figure S2). By contrast, in cells overexpressing GFP-tagged LHFPL4, the prevalence of inhibitory or excitatory hemi-synapses did not differ from that seen in control cells expressing GFP alone (Figure S2). Thus, LHFPL4 does not have synaptogenic properties.

Loss of LHFPL4 Leads to Reduced GABA$_A$R Clustering and Aggregates of Mis-localized Gephyrin in Intact Brain

We further explored the relationship between GABA$_A$Rs and LHFPL4 in whole brain samples. Importantly, an LHFPL4 antibody readily co-immunoprecipitated the GABA$_A$R-α1 subunit, gephyrin, and the inhibitory postsynaptic adhesion molecule neuroligin2 from WT but not *Lhfpl4*$^{-/-}$ brain lysate. In addition, LHFPL4 could be co-immunoprecipitated with a neuroligin2 antibody in the reciprocal experiment (Figures 4A and 4B). Thus, LHFPL4 can form native complexes in vivo with key components of the inhibitory scaffold in addition to GABA$_A$Rs.

To address how loss of LHFPL4 affected GABA$_A$R clustering and inhibitory synapse integrity in the intact brain, we carried out immunohistochemistry on fixed brain sections from adult WT and *Lhfpl4*$^{-/-}$ mice. Consistent with the loss of GABA$_A$R clustering seen in cultured neurons, labeling with a GABA$_A$R-α2 antibody revealed a dramatic decrease in total GABA$_A$R cluster area in the hippocampal CA1 region of *Lhfpl4*$^{-/-}$ mice (Figures 4C and 4D). Labeling with a gephyrin antibody revealed a significant loss of total gephyrin cluster area (Figures 4E and 4F), indicating a parallel disruption of the inhibitory postsynaptic domain. The inhibitory presynaptic domain, revealed by labeling

(K and L) Confocal images (K) and cluster quantification (L) of GFP or LHFPL4^{GFP} transfected WT or *Lhfpl4*$^{-/-}$ neurons labeled with a gephyrin antibody. LHFPL4^{GFP} overexpression completely rescues the reduction of gephyrin cluster number (+GFP: WT, 7.1 ± 1.2; *Lhfpl4*$^{-/-}$, 1.9 ± 0.5; 291/80 WT/*Lhfpl4*$^{-/-}$ clusters; +LHFPL4: WT, 6.9 ± 1.1; *Lhfpl4*$^{-/-}$, 6.5 ± 0.9; 291/266 WT/*Lhfpl4*$^{-/-}$ clusters) and area (+GFP: WT, 2.9 μm^2 ± 0.5 μm^2; *Lhfpl4*$^{-/-}$, 0.5 μm^2 ± 0.2 μm^2; +LHFPL4: WT, 1.9 μm^2 ± 0.3 μm^2; *Lhfpl4*$^{-/-}$, 1.9 μm^2 ± 0.3 μm^2) seen in *Lhfpl4*$^{-/-}$ neurons. n = 14 cells per condition from 3 independent preparations: p < 0.0001 for cluster number and cluster area. Kruskal-Wallis one-way ANOVA.

Asterisks indicate results of Dunn's multiple comparison tests. *p < 0.05; **p < 0.01; ***p < 0.001; n.s., not significant. In (E)–(H), (J), and (L), error bars indicate mean ± SEM. Scale, 2 μm.

OPEN ACCESS
CellPress

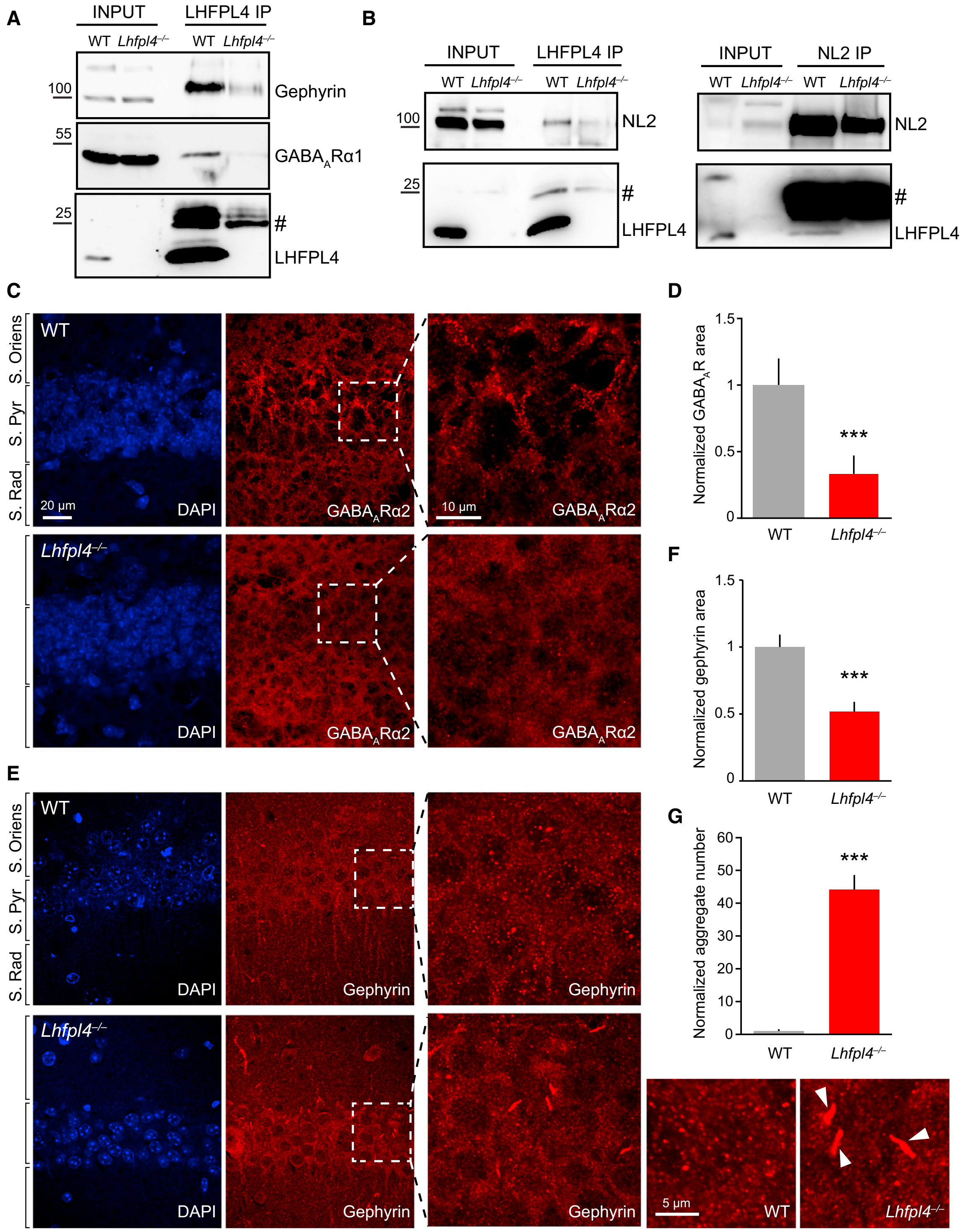

Figure 4. LHFPL4 Is Necessary for Gephyrin and GABA$_A$R Clustering in Intact Brain

(A) Co-immunoprecipitation (coIP) of gephyrin and GABA$_A$R-α1 using an anti-LHFPL4 antibody from WT and *Lhfpl4*$^{-/-}$ mouse brain lysates (IP, immunoprecipitation; #, immunoglobulin G [IgG] light chain). Note that the immunoprecipitated 27-kDa band is visible just above the IgG light chain in the WT IP lane.

(B) CoIP of neuroligin2 (NL2) using an anti-LHFPL4 antibody (left) and coIP of LHFPL4 using an anti-NL2 antibody (right) from WT and *Lhfpl4*$^{-/-}$ mouse brain lysates (IP, immunoprecipitation; #, IgG light chain).

(C–F) Confocal images of adult WT and *Lhfpl4*$^{-/-}$ hippocampal brain sections immunolabeled with antibodies to (C) GABA$_A$R-α2 and (E) gephyrin, co-stained with DAPI. Normalized total cluster area quantification of (D) GABA$_A$R-α2 and (F) gephyrin showing a loss of GABA$_A$R-α2 (from 1.0 ± 0.2 to 0.3 ± 0.1; n = 17 WT

(legend continued on next page)

with glutamate decarboxylase (GAD6) antibody, remained intact (Figures S3A and S3B). Remarkably, we also observed the presence of large aggregates of mis-localized gephyrin within the soma and dendrites of *Lhfpl4*$^{-/-}$ neurons (Figures 4E and 4G), associated with a significant decrease in GAD6/gephyrin-positive clusters (Figure S3C). The dramatic re-distribution of gephyrin, along with the loss of $GABA_AR$-α2 subunit clustering, is consistent with LHFPL4 playing a key role in maintaining $GABA_A$Rs and their associated scaffold at inhibitory synaptic sites.

Loss of LHFPL4 Disrupts Inhibitory Postsynaptic Currents in Cultured Neurons

To determine the functional effect of LHFPL4 deletion, we initially examined hippocampal neurons in dissociated cultures and measured charge transfer mediated by miniature inhibitory postsynaptic currents (mIPSCs). When compared to neurons from WT littermates, synaptic charge in *Lhfpl4*$^{-/-}$ neurons was reduced by ~60% (Figures 5A and 5B), indicating loss of receptor/synapse number or function. Of note, we observed considerable variability in the amplitude and frequency of mIPSCs in both WT and *Lhfpl4*$^{-/-}$ cultures. This could reflect heterogeneity in the mixed hippocampal preparations and a varied contribution of LHFPL4. Indeed, immunolabeling for VGAT and $GABA_AR$-γ2 revealed a small population of neurons cultured from *Lhfpl4*$^{-/-}$ mice that appeared to maintain their inhibitory synapses, while $GABA_AR$-α2^{SEP} fluorescence also remained clustered in a proportion of cells. These observations suggested that LHFPL4 effects may be cell type specific. To test this, we compared the effect of LHFPL4 deletion on $GABA_AR$-γ2 clustering in excitatory and inhibitory neurons, identified using antibodies against CAMKIIα and GAD6, respectively. Whereas CAMKIIα-positive cells showed a loss of $GABA_AR$-γ2 clustering, GAD6-positive cells did not (Figures 5C and 5D). Thus, LHFPL4 appears to be essential for $GABA_AR$ clustering only in excitatory hippocampal neurons.

Cell-Type- and Synapse-Specific Effects of LHFPL4 Deletion

To further explore LHFPL4 function and its putative cell-type specificity, we made recordings in acute hippocampal slices from WT and *Lhfpl4*$^{-/-}$ mice. LHFPL4 deletion resulted in a profound loss of mIPSCs in CA1 pyramidal neurons (Figure 6A), with the mIPSC-mediated charge transfer reduced by ~80% (Figure 6B). Fast mIPSCs (median, 10%–90%; rise time, 0.4 ms; and $\tau_{w,\ decay}$, 12 ms), likely originating from perisomatically projecting basket cells (Miles et al., 1996), were markedly reduced in both frequency and amplitude (Figure 6B), although their rise and decay were not changed (Figures 6C and 6D). By contrast, slow mIPSCs (Pearce, 1993) (median, 10%–90%; rise time, 9 ms; and $\tau_{w,\ decay}$, 21 ms), of the type thought to originate from neurogliaform/Ivy cells (Armstrong et al., 2012; Szabadics et al., 2007), were modestly increased in frequency and unaltered in amplitude (Figures 6E and 6F). Importantly, LHFPL4 deletion had no effect on AMPAR-mediated miniature excitatory postsynaptic currents (mEPSCs) (Figures 6G and 6H), confirming a selective effect on inhibitory synapses.

We next examined the effect of LHFPL4 deletion on bicuculline-sensitive tonic currents reflecting the persistent activation of extrasynaptic $GABA_A$Rs (Farrant and Nusser, 2005) (Figures 7A–7C). In CA1 pyramidal cells from *Lhfpl4*$^{-/-}$ mice, the magnitude of the tonic current was slightly increased (Figure 7C), suggesting that LHFPL4 is required for the targeting of synaptic, but not extrasynaptic, $GABA_A$Rs. Finally, to determine whether the functional effects of LHFPL4 were, indeed, cell type specific, we examined presumptive inhibitory interneurons. In marked contrast to the profound loss of mIPSC-mediated charge transfer seen in CA1 pyramidal cells, charge transfer in non-pyramidal cells in CA1 stratum oriens, radiatum, or lacunosum-moleculare from *Lhfpl4*$^{-/-}$ mice was unaffected (Figures 7D and 7E).

DISCUSSION

As clustering of $GABA_A$Rs at synapses is essential for correct inhibitory signaling in the brain (Luscher et al., 2011b; Smith and Kittler, 2010; Tyagarajan and Fritschy, 2014; Vithlani et al., 2011), it is vital to understand the machinery and regulatory pathways involved. Using molecular, imaging, electrophysiological, and mouse transgenic approaches, we show that the previously uncharacterized tetraspanin, LHFPL4, forms a complex with $GABA_A$Rs, localizes to the inhibitory postsynaptic domain, and is critical for postsynaptic $GABA_AR$ clustering and fast GABAergic transmission in excitatory principal cells.

At inhibitory synapses, the canonical scaffold gephyrin, in complex with collybistin and the *trans*-synaptic adhesion molecule neuroligin2, plays a key role in $GABA_AR$ clustering and anchoring (reviewed in Tyagarajan and Fritschy, 2014; Varoqueaux et al., 2004). Nevertheless, in the absence of gephyrin, subsets of inhibitory synapses remain (Kneussel et al., 1999; O'Sullivan et al., 2009; Panzanelli et al., 2011). Moreover, the role for collybistin in inhibitory synapse formation appears to be region specific (Papadopoulos et al., 2007). Thus, there has been great interest in identifying other molecules that drive $GABA_AR$ clustering in a cell-type- or synapse-specific manner.

We found LHFPL4 to be enriched at inhibitory postsynaptic sites, and SIM imaging revealed clusters of LHFPL4 to overlay gephyrin clusters, placing it in the exact location required to scaffold $GABA_A$Rs at the synapse. Importantly, we did not find LHFPL4 at excitatory synapses, suggesting that it is not a general synaptic organizer but rather an exclusive regulator of the inhibitory synaptic domain. This is supported by our finding that LHFPL4 and $GABA_A$Rs interact with high affinity and that deletion of LHFPL4 leads to a dramatic loss of $GABA_AR$ and gephyrin clustering, while excitatory synapses are unaffected. It is

and 19 KO hippocampi from 4 animals per genotype; $p = 0.0083$) and gephyrin (from 1.0 ± 0.1 to 0.5 ± 0.1; n = 16 WT and 12 KO hippocampi from 3 animals per genotype; $p = 0.0004$) clustering in *Lhfpl4*$^{-/-}$ tissue.
(G) Bar graph showing increased large gephyrin aggregates in *Lhfpl4*$^{-/-}$ compared to WT brain slices (from 1.0 ± 0.6 to 44.2 ± 4.4; n = 15 WT and 14 KO regions from 3 animals per genotype; $p < 0.0001$). White arrowheads indicate gephyrin aggregates in the zoom image. In (D), (F), and (G), error bars indicate mean ± SEM. *$p < 0.05$; ***$p < 0.001$ (Welch t tests).

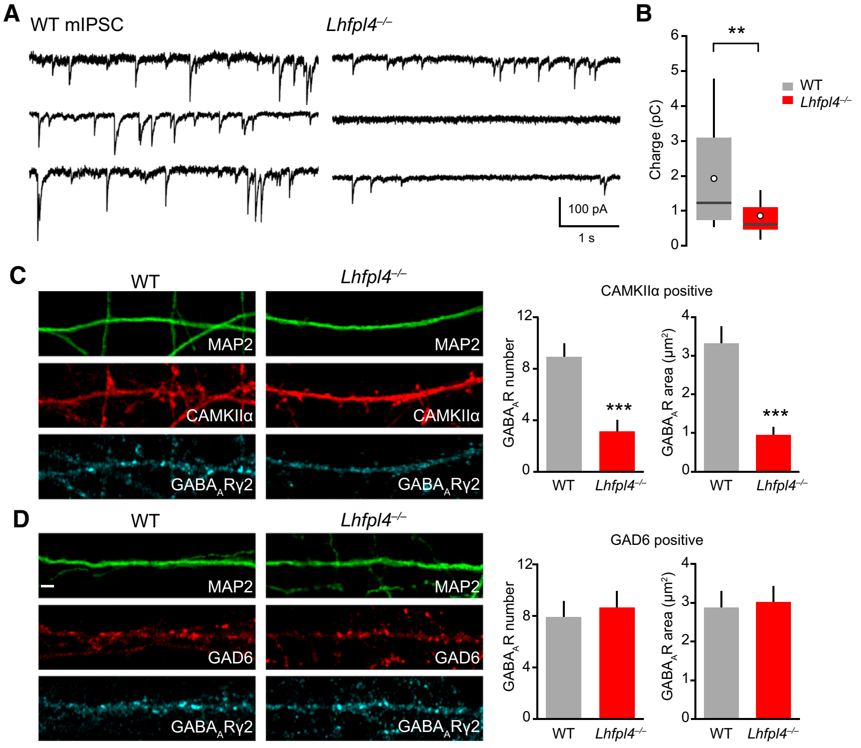

Figure 5. Selective Loss of mIPSCs in Cultured *Lhfpl4*$^{-/-}$ Neurons

(A) Representative recordings of mIPSCs (−70 mV) in cultured hippocampal neurons prepared from WT mice (left) and *Lhfpl4*$^{-/-}$ mice (right). In each case, recordings from three different neurons are shown. Records digitally filtered at 2 kHz for illustration purposes.

(B) Pooled data showing reduction in mean mIPSC charge transfer (from 1.93 pC ± 0.37 pC to 0.86 pC ± 0.12 pC; n = 18 WT and 24 *Lhfpl4*$^{-/-}$ cells; p = 0.0058, Wilcoxon rank-sum test). Box-and-whisker plots indicate median (line), 25th–75th percentiles (box), the range of data within 1.5 × interquartile range (IQR) of box (whiskers), and mean (open circles).

(C and D) Confocal images and cluster quantification of dissociated DIV14 WT or *Lhfpl4*$^{-/-}$ hippocampal neurons transfected with GFP and labeled with antibodies to $GABA_AR$-γ2 and either (C) CAMKIIα or (D) GAD6 to label excitatory neurons and inhibitory neurons, respectively. $GABA_AR$ cluster number and area were significantly reduced in *Lhfpl4*$^{-/-}$ CAMKIIα-positive cells (cluster number: from 8.9 ± 1.0 to 3.1 ± 0.9; area: from 3.3 μm^2 ± 0.4 μm^2 to 1.0 μm^2 ± 0.2 μm^2; 362/131 WT/*Lhfpl4*$^{-/-}$ clusters; p = 0.00032 and 0.00013, respectively), but not GAD6-positive cells, compared to WT (cluster number: from 7.9 ± 1.2 to 8.6 ± 1.3; area: from 2.9 μm^2 ± 0.4 μm^2 to 3.0 μm^2 ± 0.4 μm^2; 376/362 WT/*Lhfpl4*$^{-/-}$ clusters; p = 0.69 and 0.81, respectively). Error bars indicate mean ± SEM. For all conditions, n = 14 cells from 3 independent preparations. All used the Welch t test. **p < 0.01, ***p < 0.001. Scale, 2 μm.

currently thought that $GABA_A$Rs are initially trafficked extrasynaptically and subsequently diffuse to, and are trapped at, synaptic sites (Bannai et al., 2009; Bogdanov et al., 2006; Luscher et al., 2011b). In *Lhfpl4*$^{-/-}$ neurons, $GABA_A$Rs were still found at the neuronal surface. This suggests that, in the absence of LHFPL4, the cell-surface $GABA_A$R trafficking pathways are preserved but that, once at the cell surface, receptors can no longer be trapped at synapses.

It is well established that synaptic $GABA_A$Rs are reciprocally required for the clustering of gephyrin (Tyagarajan and Fritschy, 2014). Thus, it is difficult to determine whether the loss of gephyrin clustering we observed upon LHFPL4 deletion is a cause or a consequence of the LHFPL4-dependent disruption of $GABA_A$R clustering. In this regard, it has previously been described that, in hippocampal and thalamic relay neurons, subunit-specific knockout of $GABA_A$Rs leads to the accumulation of large intracellular gephyrin aggregates (Studer et al., 2006). This phenomenon was attributed to the need for synaptic $GABA_A$Rs to maintain gephyrin clustering (Essrich et al., 1998). Interestingly, we found that loss of LHFPL4 in vivo leads to a similar accumulation of intracellular gephyrin aggregates in the cell-body layer of the hippocampus.

Accompanying the loss of $GABA_A$R clusters, in CA1 pyramidal neurons from *Lhfpl4*$^{-/-}$ mice, we observed a dramatic decrease in both the amplitude and frequency of fast mIPSCs. The reduced amplitude of mIPSCs would, most straightforwardly, reflect loss of postsynaptic receptors. The primarily postsynaptic action of LHFPL4 is supported by the fact that, in our rescue experiments, sparse re-expression of LHFPL4 cDNA in *Lhfpl4*$^{-/-}$ neurons readily rescued postsynaptic $GABA_A$R clustering. The reduced frequency of mIPSCs could reflect both direct and indirect effects of such receptor loss; namely, a reduction of mIPSC amplitude below the threshold for detection and a possible loss of inhibitory synapses. Indeed, $GABA_A$Rs have been shown to have synaptogenic properties (Fuchs et al., 2013). Surprisingly, however, we detected no significant loss of VGAT clusters in the absence of LHFPL4, suggesting that the marked reduction in mIPSC frequency is primarily due to the postsynaptic loss of receptors and not due to a loss of inhibitory input. Similarly, deletion of InSyn1, a newly identified gephyrin-interacting protein (the loss of which results in reduced postsynaptic $GABA_A$R clustering), was also reported to lead to a marked reduction in mIPSC frequency (Uezu et al., 2016).

While fast mIPSCs were greatly reduced in *Lhfpl4*$^{-/-}$ pyramidal cells, slow mIPSCs were not. The slow mIPSCs had kinetics similar to those of currents generated by inputs from neurogliaform/Ivy cells (Armstrong et al., 2012). Interestingly, tonic inhibition was also preserved in *Lhfpl4*$^{-/-}$ neurons; indeed, it was slightly increased, possibly in response to the loss of fast phasic currents. In CA1 pyramidal neurons, both the slow IPSCs arising from neurogliaform cells and the tonic GABA-mediated currents are thought to be mediated by α5-subunit-containing $GABA_A$Rs (Caraiscos et al., 2004; Karayannis et al., 2010). Thus, our results suggest that LHFPL4 may drive $GABA_A$R clustering that is both synapse and subunit specific. The action of LHFPL4 could be seen as complimentary to that of radixin, which mediates

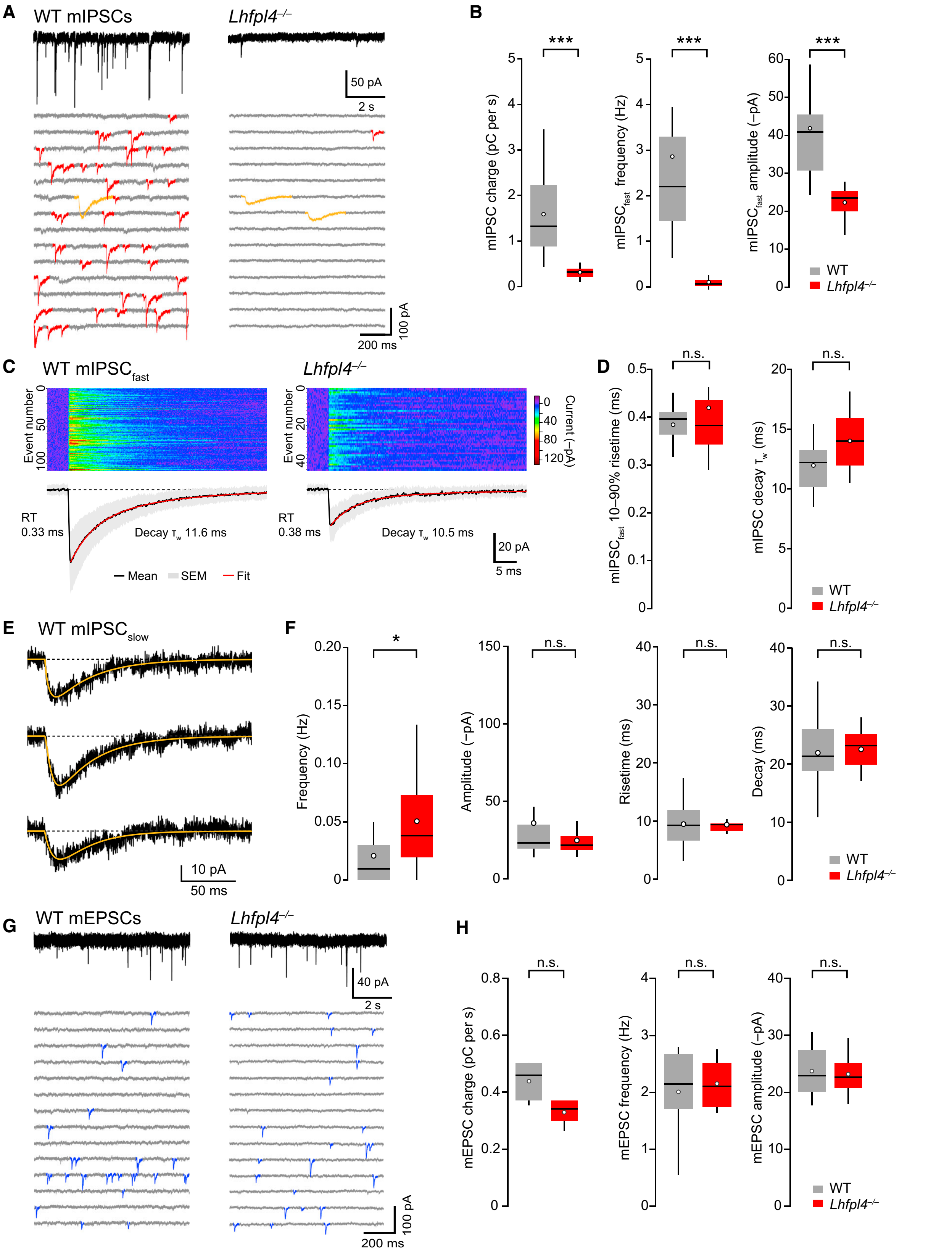

(legend on next page)

extrasynaptic trapping of α5-subunit-containing $GABA_A$Rs (Loebrich et al., 2006).

The postsynaptic single-pass membrane-spanning molecules neuroligin2, calsyntenin3, and slitrk3 promote the development and stabilization of mammalian GABAergic synapses through transynaptic interactions with presynaptic proteins—neurexins and PTPδ, respectively. Calsyntenin3 and slitrk3 loss of function leads primarily to a reduction in mIPSC frequency, while disruption of neuroligin2 causes both frequency and amplitude reductions in several cell types (Pettem et al., 2013; Poulopoulos et al., 2009; Takahashi et al., 2012). Although we identified LHFPL4 as a membrane-spanning inhibitory synaptic protein, we found no evidence that it could drive the formation of synapses onto transfected non-neuronal cells. Moreover, overexpression of LHFPL4 did not increase synapse formation in neurons. This suggests that LHFPL4 does not mediate $GABA_A$R clustering through synapse specification and *trans*-synaptic interaction with presynaptic terminals but, rather, acts to stabilize postsynaptic $GABA_A$R clusters, possibly through enhancing interactions between $GABA_A$Rs and synaptogenic molecules such as neuroligin2.

Understanding the processes that mediate cell- and synapse-specific $GABA_A$R clustering is key to a better understanding of the underlying logic of inhibitory control in the brain. The homophilic adhesion molecule IgSF9b was recently demonstrated to promote inhibitory synapse development in interneurons by coupling to neuroligin2 and S-SCAM (Woo et al., 2013). By contrast, we show here that the impact of LHFPL4 deletion on $GABA_A$R clustering and synaptic inhibition in the hippocampus is specific to CamKIIα-positive excitatory principal cells. As we found LHFPL4 to interact tightly with neuroligin2, it is likely that neuroligins work in concert with an array of clustering molecules to drive cell-specific regulation of postsynaptic receptor clustering. In this regard, further studies of identified interneuron types will be necessary to determine whether resistance to LHFPL4 deletion is a property of all interneurons or specific classes.

As the strength of inhibitory synaptic transmission directly correlates with the number of surface $GABA_A$Rs at synaptic sites, modulation of $GABA_A$R synaptic accumulation is a key mechanism underlying inhibitory synaptic plasticity. It will be interesting to determine whether LHFPL4 plays a role in previously reported mechanisms of activity-dependent tuning of synaptic $GABA_A$R number (Bannai et al., 2009; Muir et al., 2010; Petrini et al., 2014). Deficits in GABAergic neurotransmission can result in alterations in information processing at the network level and have been implicated in multiple neuropsychiatric disorders (Blundell et al., 2009; Charych et al., 2009; Crestani et al., 1999; Luscher et al., 2011a; Yizhar et al., 2011). Thus, identification of the molecular mechanisms by which LHFPL4 regulates inhibitory transmission may be critical to understanding both normal and disordered states. In line with other knockout mouse models that result in disrupted $GABA_A$R and gephyrin clustering, such as the $GABA_A$R-α1 or β2 subunit knockouts, we saw no obvious home cage behavioral deficits in *Lhfpl4*$^{-/-}$ animals (Kralic et al., 2006; Sur et al., 2001; Vicini et al., 2001). Further detailed characterization will be necessary to identify any disease-associated behavioral deficits, such as the anxiety- and schizophrenia-related sensorimotor deficits observed in neuroligin2 and $GABA_A$R-α3 knockout mice, respectively (Blundell et al., 2009; Yee et al., 2005).

Very recently, a paper describing LHFPL4 as a $GABA_A$R regulatory Lhfpl (GARLH) protein was published (Yamasaki et al., 2017). Although not the first to establish LHFPL4 as a $GABA_A$R-interacting protein (Heller et al., 2012; Nakamura et al., 2016), these authors identified a tripartite interaction between $GABA_A$Rs, neuroligin2, and LHFPL4. Using the complimentary approaches of short hairpin RNA (shRNA)-mediated LHFPL4 knockdown in culture and virally mediated CRISPR knockout in Cre-dependent Cas9 knockin mice, they also reported a marked reduction in mIPSC frequency in hippocampal CA1 neurons. Of note, here we further demonstrate that LHFPL4 is essential for inhibitory synapse stability in CA1 pyramidal cells

Figure 6. Loss of Fast mIPSCs in CA1 Pyramidal Neurons from *Lhfpl4*$^{-/-}$ Mice

(A) Representative recordings of mIPSCs (−70 mV) in CA1 pyramidal cells from a WT mouse (left) and an *Lhfpl4*$^{-/-}$ mouse (right). Lower panels are representative sections of recordings (contiguous 1-s segments) showing a loss of fast mIPSCs (red) but a maintained presence of slow mIPSCs (orange). Records are digitally filtered at 2 kHz for illustration purposes.

(B) Pooled data showing reduction in mean mIPSC charge transfer (from 1.59 pC ± 0.21 pC to 0.31 pC ± 0.11 pC; n = 18 WT and 18 *Lhfpl4*$^{-/-}$ cells), frequency (from 2.86 Hz ± 0.54 Hz to 0.10 Hz ± 0.03 Hz; n = 18 WT and 18 *Lhfpl4*$^{-/-}$ cells), and amplitude (from 141.9 pA ± 3.8 pA to 22.3 pA ± 1.1 pA; n = 18 WT and 14 *Lhfpl4*$^{-/-}$ cells). All $p < 0.0001$, Wilcoxon rank-sum test.

(C) Top: images illustrating the alignment and amplitudes of selected mIPSCs with uncontaminated rise and decay from representative WT and *Lhfpl4*$^{-/-}$ recordings. Bottom: average mIPSC waveforms (black), SEM (gray), and fitted sum of exponentials (red). The 10%–90% rise times and weighted time constant of decay (τ_w) are shown for each representative record.

(D) Pooled data showing the lack of effect on mIPSC rise time (0.38 ms ± 0.01 ms and 0.42 ms ± 0.04 ms; n = 18 WT and 11 *Lhfpl4*$^{-/-}$ cells; p = 0.86, Wilcoxon rank-sum test) and decay (12.0 ms ± 0.5 ms and 14.0 ms ± 1.4 ms; n = 18 WT and 11 *Lhfpl4*$^{-/-}$ cells; p = 0.20, Welch t test).

(E) Representative slow mIPSCs (−70 mV) from CA1 pyramidal neurons (three different WT mice). Orange lines indicate fits of an empirical equation (see the Supplemental Experimental Procedures) from which 10%–90% rise time and 63% decay time measures were taken.

(F) Pooled data showing the modest increase in frequency of slow mIPSCs in *Lhfpl4*$^{-/-}$ mice (from 0.021 Hz ± 0.007 Hz to 0.051 Hz ± 0.01 Hz; n = 18 WT and 18 *Lhfpl4*$^{-/-}$ cells; p = 0.014, Wilcoxon rank-sum test) but a lack of change in amplitude (36.0 pA ± 8.4 pA and 24.8 pA ± 2.4 pA; n = 12 WT and 16 *Lhfpl4*$^{-/-}$ cells; p = 0.37 Wilcoxon rank-sum test), rise time (9.5 ms ± 1.1 ms and 9.4 ms ± 0.4 ms; n = 12 WT and 16 *Lhfpl4*$^{-/-}$ cells; p = 0.77, Welch t test), and decay (21.9 ms ± 1.7 ms and 22.5 ms ± 0.9 ms; n = 12 WT and 16 *Lhfpl4*$^{-/-}$ cells; p = 0.93, Welch t test). Box-and-whisker plots indicate median (line), 25th–75th percentiles (box), the range of data within 1.5 × IQR of the box (whiskers), and mean (open circles).

(G) Representative recordings of mEPSCs (−70 mV) in CA1 pyramidal cells from a WT mouse (left) and a *Lhfpl4*$^{-/-}$ mouse (right). Lower panels are representative sections of recordings (as in A), with mEPSCs in blue.

(H) Pooled data showing no change in mean mEPSC charge transfer (0.44 pC ± 0.07 pC and 0.33 pC ± 0.03 pC), frequency (2.01 Hz ± 0.35 Hz and 2.15 Hz ± 0.27 Hz), and amplitude (23.7 pA ± 2.1 pA and 23.2 pA ± 2.4 pA). In each case, n = 6 WT and 4 *Lhfpl4*$^{-/-}$ cells (p = 0.21, 0.75, and 0.87, respectively; Welch t test). See also the Supplemental Experimental Procedures.

OPEN
ACCESS
CellPress

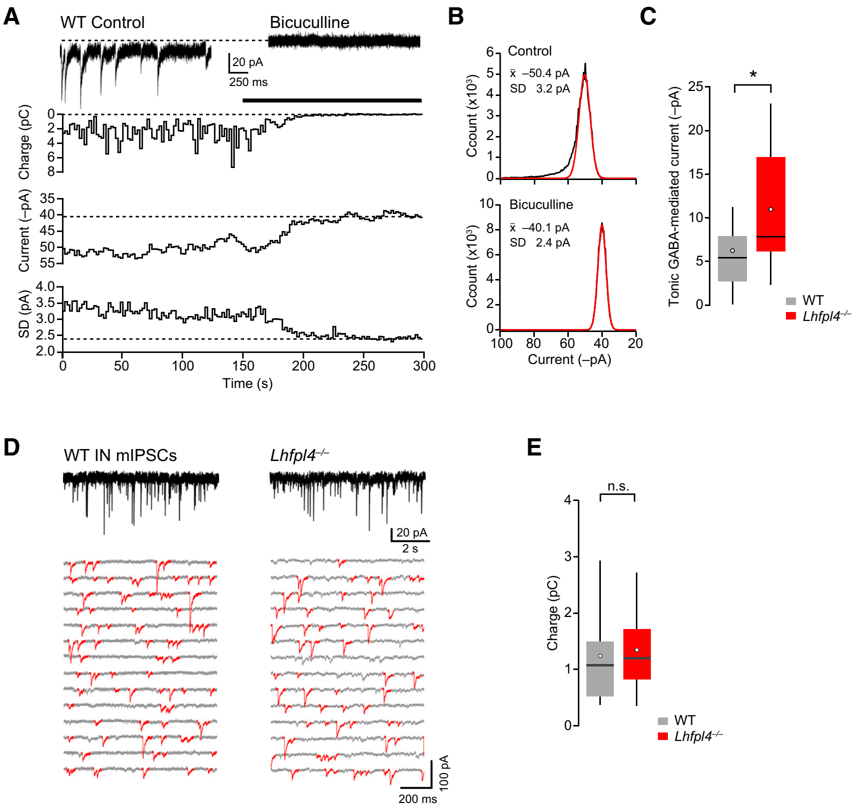

Figure 7. GABA-Mediated Tonic Current in Pyramidal Cells and mIPSCs in Interneurons Are Not Disrupted by LHFPL4 Deletion

(A) Representative record (−70 mV) from a WT CA1 pyramidal neuron showing the block of mIPSCs and the shift in holding current produced by bath application of bicuculline. Lower panels show the time course of the synaptic charge transfer, mean holding current, and its SD (see Supplemental Experimental Procedures).

(B) Representative all-point amplitude histograms from 1-s segments in the control period (upper) and in the presence of bicuculline (lower). Red lines indicate fits of a single-sided Gaussians to the most positive current values yielding the mean and the SD. The mean was taken as the baseline current for that segment and subtracted from the record. The integral of the subtracted current provided the charge carried by the synaptic events.

(C) Pooled data showing increased tonic GABA-mediated current in CA1 pyramidal neurons (from 6.2 pA ± 1.3 pA to 11.0 pA ± 1.7 pA; n = 22 WT and 15 *Lhfpl4*$^{-/-}$ cells; $p = 0.037$, Welch t test).

(D) Representative recordings of mIPSCs (−70 mV) in non-pyramidal cells (INs, presumptive interneurons) from a WT mouse (left) and an *Lhfpl4*$^{-/-}$ mouse (right). Lower panels are representative sections of recordings (contiguous 1-s segments) showing no change in fast mIPSCs (red). Records digitally filtered at 2 kHz for illustration purposes.

(E) Pooled data showing the lack of effect of LHFPL4 deletion on mIPSC-mediated charge transfer in presumptive interneurons (1.24 pC ± 0.39 pC and 1.34 pC ± 0.34 pC; n = 6 WT and 6 *Lhfpl4*$^{-/-}$ cells; $p = 0.84$, Welch t test).

Box-and-whisker plots indicate median (line), 25th–75th percentiles (box), the range of data within 1.5 × IQR of the box (whiskers), and mean (open circles). *$p < 0.05$.

See also the Supplemental Experimental Procedures.

but not in hippocampal interneurons. Moreover, we additionally observed that fast, but not slow, mIPSCs are reduced in *Lhfpl4*$^{-/-}$ mice, suggesting that LHFPL4 effects may be not only cell type specific but also synapse specific. It will be interesting to determine whether the modestly increased frequency of slow mIPSCs and increased tonic inhibition we identified in *Lhfpl4*$^{-/-}$ mice are adaptive changes due to prolonged disruption of synaptic inhibition (Brickley et al., 2001).

Members of the tetraspanin superfamily of integral membrane proteins have emerged as key regulators of excitatory synaptic function. Notably, TARPs and GSG1L have been shown to associate with AMPARs to regulate their trafficking and functional properties (Jackson and Nicoll, 2011; McGee et al., 2015). Our findings provide new insights into the molecular make-up of the inhibitory PSD and reveal a key role for an inhibitory synapse-specific tetraspanin. LHFPL4 is one of a subfamily of five tetraspanins that includes the homologous LHFPL3 and LHFPL5 and the more distantly related LHFPL1 and LHFPL2. Interestingly, LHFPL5 has been implicated in the mechanotransduction pathway of the inner hair cell, and its dysfunction contributes to hearing loss in humans and mice (György et al., 2017; Kalay et al., 2006; Longo-Guess et al., 2005; Xiong et al., 2012). Whether the other family members also have roles in synaptic function remains to be determined.

EXPERIMENTAL PROCEDURES

Details regarding animals, antibodies, immunocytochemistry and immunohistochemistry, co-immunoprecipitations, biotinylations, cDNA cloning, and data analysis are included in the Supplemental Experimental Procedures.

Cell Culture and Transfections

Hippocampal cultures were obtained from E16 mouse or E18 rat embryos of either sex, as previously described (López-Doménech et al., 2016; Vaccaro et al., 2017). All procedures for the care and treatment of animals were in accordance with the Animals (Scientific Procedures) Act 1986. Neurons were transfected using Lipofectamine 2000 (Invitrogen). COS-7 cells were maintained in DMEM supplemented with fetal calf serum and antibiotics and were transfected using the Amaxa Nucleofector device (Lonza) following the manufacturer's protocol.

Microscopy

Confocal images were acquired on a Zeiss LSM700 upright confocal microscope using a 63× oil objective (NA: 1.4) and digitally captured using LSM software. For cultured neurons, a whole-cell single-plane image was captured using a 0.5× zoom. From this, 3 sections of dendrite, ~100 μm from the soma, were imaged with a 3× zoom (equating to a 30-μm length of dendrite). For brain sections from adult male and female fixed brains, a low-magnification region of the hippocampus was captured using a 63× objective and 0.5× zoom. From this, 2–3 zoom regions were imaged within each hippocampal strata with a 2× zoom for analysis. Acquisition settings and laser power were kept constant within experiments. For details of

antibody labeling and image analysis, see Supplemental Experimental Procedures.

SIM was performed on a commercial Zeiss ELYRA PS.1 inverted microscope. Images were acquired with a 63× oil objective lens (NA: 1.4) using a pco.edge sCMOS camera and ZEN Black (v.11.0.2.190) software (2,430 × 2,430 pixels, 78.32-μm^2 image size, 16 bit). Typically, images were acquired with 34-μm grating and three rotations by exciting fluorophores with 1%–3% laser intensity and 120-ms to 150-ms exposure time. Images were processed with ZEN Black using the SIM reconstruction module with default settings; drift corrections between the channels were performed with respect to 100-nm Tetraspec fluorescent microspheres (Molecular Probes).

Electrophysiology

Standard whole-cell voltage-clamp techniques were used to record mIPSCs in cultured neurons. For details, see the Supplemental Experimental Procedures. For slice electrophysiology, hippocampal slices from male and female mice (P30–P45) were perfused at room temperature with external solution containing (in millimolar): 125 NaCl, 2.5 KCl, 1 $MgCl_2$, 1.25 NaH_2PO_4, 2 $CaCl_2$, 38 glucose, and 26 $NaHCO_3$ saturated with 95% O_2/5% CO_2, (pH 7.4). Cells were visualized using oblique illumination. Currents were recorded using a MultiClamp 700B amplifier (Molecular Devices), filtered at 4 kHz, and digitized at 50 kHz using WinWCP and WinEDR (Strathclyde Electrophysiology Software) and an InstruTECH ITC-18 interface (HEKA Elektronik). Series resistance was typically compensated by 50%–80%, and data were discarded if the series resistance varied by >20%.

For mIPSCs and tonic current measurement, recording pipettes were filled with an internal solution containing (in millimolar): 128 CsCl, 10 EGTA-Cs, 10 HEPES, 2 MgATP, 1 $CaCl_2$, 2 NaCl, 1 QX-314 (Tocris Bioscience), and 5 TEA-Cl (adjusted to pH 7.3 with CsOH). In some cases, this solution also contained 0.2% biocytin (Molecular Probes). D-AP5 (20 μM, Abcam) and NBQX (10 μM, Abcam) were added to the external solution to block NMDA receptors (NMDARs) and AMPARs. All mIPSCs were blocked by bicuculline (20 μM, Tocris) or gabazine (20 μM, Abcam). mEPSCs were recorded at 32.5°C using an internal solution containing (in millimolar) 135 Cs-gluconate, 10 HEPES, 10 Na-phosphocreatine, 4 MgATP, 0.4 NaGTP, 2 QX-314, and 10 TEA-Cl (adjusted to pH 7.3 with CsOH). The external solution contained an additional 5 mM KCl and bicuculline (20 μM) or gabazine (20 μM). All mEPSCs were blocked with NBQX (10 μM; Abcam or Tocris). Interneurons were identified under infrared video microscopy by their relatively small and rounded or ovoid cell bodies, compared to the large triangular somata of pyramidal cells, and by the absence of conspicuous apical dendrites. Their cell bodies were located in CA1 stratum oriens, radiatum, or lacunosum-moleculare. Although reconstruction was not performed, post hoc examination of each biocytin-filled putative interneuron confirmed its location outside the pyramidal cell layer and its non-pyramidal cell morphology.

Statistics

All data were obtained from at least three different cell preparations or animals. Data are reported as mean ± SEM. Repeats for experiments are given in the figure legends as n numbers. No statistical test was used to pre-determine sample sizes; these were based on standards of the field. Statistical analyses were carried out using GraphPad Prism (GraphPad Software, La Jolla CA, USA), Microsoft Excel or R (v.3.2.3; the R Foundation for Statistical Computing; http://www.r-project.org/), and R Studio (v.0.99.893; RStudio). Data were tested for normality (D'Agostino-Pearson test or Shapiro-Wilk test) and compared using either parametric (unpaired Welch t test) or non-parametric tests (Wilcoxon rank-sum test or Kruskal-Wallis one-way ANOVA). Exact p values are presented to two significant figures, except when $p < 0.0001$. Differences were considered significant at $p < 0.05$. No blinding or randomization was used.

SUPPLEMENTAL INFORMATION

Supplemental Information includes Supplemental Experimental Procedures and three figures and can be found with this article online at http://dx.doi.org/10.1016/j.celrep.2017.09.025.

AUTHOR CONTRIBUTIONS

This study was conceived by J.T.K. Experiments were designed by J.T.K., E.C.D., and M.F. Experiments were performed by E.C.D., V.P., G.K., T.P.M., D.F.S., and G.L.-D. Data were analyzed by E.C.D., V.P., G.K., M.F., and J.T.K., and the manuscript was written by E.C.D., M.F., and J.T.K.

ACKNOWLEDGMENTS

This work was funded by the UK Medical Research Council (G0802377 and MR/N025644/1 to J.T.K. and J002976/1 and J012998/1 to M.F.), the Lister Institute of Preventive Medicine, and the ERC (282430 to J.T.K.). E.C.D. and G.K. were in the MRC LMCB PhD program, and D.F.S. was in the UCL-CoMPLEX PhD program. We thank the UCL Super-resolution Facility (funded by the MRC Next Generation Optical Microscopy Initiative) and the MRC LMCB Light Microscopy staff for their contributions.

Received: March 7, 2017
Revised: July 17, 2017
Accepted: September 6, 2017
Published: October 3, 2017

REFERENCES

Armstrong, C., Krook-Magnuson, E., and Soltesz, I. (2012). Neurogliaform and ivy cells: a major family of nNOS expressing GABAergic neurons. Front. Neural Circuits *6*, 23.

Bannai, H., Lévi, S., Schweizer, C., Inoue, T., Launey, T., Racine, V., Sibarita, J.-B., Mikoshiba, K., and Triller, A. (2009). Activity-dependent tuning of inhibitory neurotransmission based on $GABA_AR$ diffusion dynamics. Neuron *62*, 670–682.

Blundell, J., Tabuchi, K., Bolliger, M.F., Blaiss, C.A., Brose, N., Liu, X., Südhof, T.C., and Powell, C.M. (2009). Increased anxiety-like behavior in mice lacking the inhibitory synapse cell adhesion molecule neuroligin 2. Genes Brain Behav. *8*, 114–126.

Bogdanov, Y., Michels, G., Armstrong-Gold, C., Haydon, P.G., Lindstrom, J., Pangalos, M., and Moss, S.J. (2006). Synaptic $GABA_A$ receptors are directly recruited from their extrasynaptic counterparts. EMBO J. *25*, 4381–4389.

Brickley, S.G., Revilla, V., Cull-Candy, S.G., Wisden, W., and Farrant, M. (2001). Adaptive regulation of neuronal excitability by a voltage-independent potassium conductance. Nature *409*, 88–92.

Caraiscos, V.B., Elliott, E.M., You-Ten, K.E., Cheng, V.Y., Belelli, D., Newell, J.G., Jackson, M.F., Lambert, J.J., Rosahl, T.W., Wafford, K.A., et al. (2004). Tonic inhibition in mouse hippocampal CA1 pyramidal neurons is mediated by alpha5 subunit-containing gamma-aminobutyric acid type A receptors. Proc. Natl. Acad. Sci. USA *101*, 3662–3667.

Charych, E.I., Liu, F., Moss, S.J., and Brandon, N.J. (2009). GABA(A) receptors and their associated proteins: implications in the etiology and treatment of schizophrenia and related disorders. Neuropharmacology *57*, 481–495.

Chih, B., Gollan, L., and Scheiffele, P. (2006). Alternative splicing controls selective trans-synaptic interactions of the neuroligin-neurexin complex. Neuron *51*, 171–178.

Copits, B.A., and Swanson, G.T. (2012). Dancing partners at the synapse: auxiliary subunits that shape kainate receptor function. Nat. Rev. Neurosci. *13*, 675–686.

Crestani, F., Lorez, M., Baer, K., Essrich, C., Benke, D., Laurent, J.P., Belzung, C., Fritschy, J.M., Lüscher, B., and Mohler, H. (1999). Decreased $GABA_A$-receptor clustering results in enhanced anxiety and a bias for threat cues. Nat. Neurosci. *2*, 833–839.

Eckel, R., Szulc, B., Walker, M.C., and Kittler, J.T. (2015). Activation of calcineurin underlies altered trafficking of α2 subunit containing $GABA_A$ receptors during prolonged epileptiform activity. Neuropharmacology *88*, 82–90.

Essrich, C., Lorez, M., Benson, J.A., Fritschy, J.M., and Lüscher, B. (1998). Postsynaptic clustering of major $GABA_A$ receptor subtypes requires the gamma 2 subunit and gephyrin. Nat. Neurosci. *1*, 563–571.

Farrant, M., and Nusser, Z. (2005). Variations on an inhibitory theme: phasic and tonic activation of GABA(A) receptors. Nat. Rev. Neurosci. *6*, 215–229.

Fuchs, C., Abitbol, K., Burden, J.J., Mercer, A., Brown, L., Iball, J., Stephenson, F.A., Thomson, A.M., and Jovanovic, J.N. (2013). GABA(A) receptors can initiate the formation of functional inhibitory GABAergic synapses. Eur. J. Neurosci. *38*, 3146–3158.

György, B., Sage, C., Indzhykulian, A.A., Scheffer, D.I., Brisson, A.R., Tan, S., Wu, X., Volak, A., Mu, D., Tamvakologos, P.I., et al. (2017). Rescue of hearing by gene delivery to inner-ear hair cells using exosome-associated AAV. Mol. Ther. *25*, 379–391.

Haering, S.C., Tapken, D., Pahl, S., and Hollmann, M. (2014). Auxiliary subunits: shepherding AMPA receptors to the plasma membrane. Membranes (Basel) *4*, 469–490.

Heller, E.A., Zhang, W., Selimi, F., Earnheart, J.C., Ślimak, M.A., Santos-Torres, J., Ibañez-Tallon, I., Aoki, C., Chait, B.T., and Heintz, N. (2012). The biochemical anatomy of cortical inhibitory synapses. PLoS ONE *7*, e39572.

Jackson, A.C., and Nicoll, R.A. (2011). The expanding social network of ionotropic glutamate receptors: TARPs and other transmembrane auxiliary subunits. Neuron *70*, 178–199.

Kalay, E., Li, Y., Uzumcu, A., Uyguner, O., Collin, R.W., Caylan, R., Ulubil-Emiroglu, M., Kersten, F.F.J., Hafiz, G., van Wijk, E., et al. (2006). Mutations in the *lipoma HMGIC fusion partner-like 5 (LHFPL5)* gene cause autosomal recessive nonsyndromic hearing loss. Hum. Mutat. *27*, 633–639.

Karayannis, T., Elfant, D., Huerta-Ocampo, I., Teki, S., Scott, R.S., Rusakov, D.A., Jones, M.V., and Capogna, M. (2010). Slow GABA transient and receptor desensitization shape synaptic responses evoked by hippocampal neurogliaform cells. J. Neurosci. *30*, 9898–9909.

Kneussel, M., Brandstätter, J.H., Laube, B., Stahl, S., Müller, U., and Betz, H. (1999). Loss of postsynaptic GABA(A) receptor clustering in gephyrin-deficient mice. J. Neurosci. *19*, 9289–9297.

Kralic, J.E., Sidler, C., Parpan, F., Homanics, G.E., Morrow, A.L., and Fritschy, J.-M. (2006). Compensatory alteration of inhibitory synaptic circuits in cerebellum and thalamus of gamma-aminobutyric acid type A receptor alpha1 subunit knockout mice. J. Comp. Neurol. *495*, 408–421.

Lévi, S., Logan, S.M., Tovar, K.R., and Craig, A.M. (2004). Gephyrin is critical for glycine receptor clustering but not for the formation of functional GABAergic synapses in hippocampal neurons. J. Neurosci. *24*, 207–217.

Loebrich, S., Bähring, R., Katsuno, T., Tsukita, S., and Kneussel, M. (2006). Activated radixin is essential for $GABA_A$ receptor α5 subunit anchoring at the actin cytoskeleton. EMBO J. *25*, 987–999.

Longo-Guess, C.M., Gagnon, L.H., Cook, S.A., Wu, J., Zheng, Q.Y., and Johnson, K.R. (2005). A missense mutation in the previously undescribed gene Tmhs underlies deafness in hurry-scurry (hscy) mice. Proc. Natl. Acad. Sci. USA *102*, 7894–7899.

López-Doménech, G., Higgs, N.F., Vaccaro, V., Roš, H., Arancibia-Cárcamo, I.L., MacAskill, A.F., and Kittler, J.T. (2016). Loss of dendritic complexity precedes neurodegeneration in a mouse model with disrupted mitochondrial distribution in mature dendrites. Cell Rep. *17*, 317–327.

Luscher, B., Shen, Q., and Sahir, N. (2011a). The GABAergic deficit hypothesis of major depressive disorder. Mol. Psychiatry *16*, 383–406.

Luscher, B., Fuchs, T., and Kilpatrick, C.L. (2011b). $GABA_A$ receptor trafficking-mediated plasticity of inhibitory synapses. Neuron *70*, 385–409.

McGee, T.P., Bats, C., Farrant, M., and Cull-Candy, S.G. (2015). Auxiliary subunit GSG1L acts to suppress calcium-permeable AMPA receptor function. J. Neurosci. *35*, 16171–16179.

Miles, R., Tóth, K., Gulyás, A.I., Hájos, N., and Freund, T.F. (1996). Differences between somatic and dendritic inhibition in the hippocampus. Neuron *16*, 815–823.

Muir, J., and Kittler, J.T. (2014). Plasticity of $GABA_A$ receptor diffusion dynamics at the axon initial segment. Front. Cell. Neurosci. *8*, 151.

Muir, J., Arancibia-Carcamo, I.L., MacAskill, A.F., Smith, K.R., Griffin, L.D., and Kittler, J.T. (2010). NMDA receptors regulate $GABA_A$ receptor lateral mobility and clustering at inhibitory synapses through serine 327 on the γ2 subunit. Proc. Natl. Acad. Sci. USA *107*, 16679–16684.

Nakamura, Y., Morrow, D.H., Modgil, A., Huyghe, D., Deeb, T.Z., Lumb, M.J., Davies, P.A., and Moss, S.J. (2016). Proteomic characterization of inhibitory synapses using a novel pHluorin-tagged $GABA_AR$ α2 subunit knock-in mouse. J. Biol. Chem. *291*, 12394–12407.

O'Sullivan, G.A., Hofer, W., and Betz, H. (2009). Inhibitory postsynaptic membrane specializations are formed in gephyrin-deficient mice. Neurosci. Lett. *458*, 106–110.

Panzanelli, P., Gunn, B.G., Schlatter, M.C., Benke, D., Tyagarajan, S.K., Scheiffele, P., Belelli, D., Lambert, J.J., Rudolph, U., and Fritschy, J.-M. (2011). Distinct mechanisms regulate $GABA_A$ receptor and gephyrin clustering at perisomatic and axo-axonic synapses on CA1 pyramidal cells. J. Physiol. *589*, 4959–4980.

Papadopoulos, T., Korte, M., Eulenburg, V., Kubota, H., Retiounskaia, M., Harvey, R.J., Harvey, K., O'Sullivan, G.A., Laube, B., Hülsmann, S., et al. (2007). Impaired GABAergic transmission and altered hippocampal synaptic plasticity in collybistin-deficient mice. EMBO J. *26*, 3888–3899.

Pathania, M., Davenport, E.C., Muir, J., Sheehan, D.F., López-Doménech, G., and Kittler, J.T. (2014). The autism and schizophrenia associated gene CYFIP1 is critical for the maintenance of dendritic complexity and the stabilization of mature spines. Transl. Psychiatry *4*, e374.

Pearce, R.A. (1993). Physiological evidence for two distinct $GABA_A$ responses in rat hippocampus. Neuron *10*, 189–200.

Petrini, E.M., Ravasenga, T., Hausrat, T.J., Iurilli, G., Olcese, U., Racine, V., Sibarita, J.-B., Jacob, T.C., Moss, S.J., Benfenati, F., et al. (2014). Synaptic recruitment of gephyrin regulates surface $GABA_A$ receptor dynamics for the expression of inhibitory LTP. Nat. Commun. *5*, 3921.

Pettem, K.L., Yokomaku, D., Luo, L., Linhoff, M.W., Prasad, T., Connor, S.A., Siddiqui, T.J., Kawabe, H., Chen, F., Zhang, L., et al. (2013). The specific α-neurexin interactor calsyntenin-3 promotes excitatory and inhibitory synapse development. Neuron *80*, 113–128.

Poulopoulos, A., Aramuni, G., Meyer, G., Soykan, T., Hoon, M., Papadopoulos, T., Zhang, M., Paarmann, I., Fuchs, C., Harvey, K., et al. (2009). Neuroligin 2 drives postsynaptic assembly at perisomatic inhibitory synapses through gephyrin and collybistin. Neuron *63*, 628–642.

Scheiffele, P., Fan, J., Choih, J., Fetter, R., and Serafini, T. (2000). Neuroligin expressed in nonneuronal cells triggers presynaptic development in contacting axons. Cell *101*, 657–669.

Smith, K.R., Davenport, E.C., Wei, J., Li, X., Pathania, M., Vaccaro, V., Yan, Z., and Kittler, J.T. (2014). GIT1 and βPIX are essential for GABA(A) receptor synaptic stability and inhibitory neurotransmission. Cell Rep. *9*, 298–310.

Smith, K.R., and Kittler, J.T. (2010). The cell biology of synaptic inhibition in health and disease. Curr. Opin. Neurobiol. *20*, 550–556.

Studer, R., von Boehmer, L., Haenggi, T., Schweizer, C., Benke, D., Rudolph, U., and Fritschy, J.-M. (2006). Alteration of GABAergic synapses and gephyrin clusters in the thalamic reticular nucleus of $GABA_A$ receptor alpha3 subunit-null mice. Eur. J. Neurosci. *24*, 1307–1315.

Sur, C., Wafford, K.A., Reynolds, D.S., Hadingham, K.L., Bromidge, F., Macaulay, A., Collinson, N., O'Meara, G., Howell, O., Newman, R., et al. (2001). Loss of the major GABA(A) receptor subtype in the brain is not lethal in mice. J. Neurosci. *21*, 3409–3418.

Szabadics, J., Tamás, G., and Soltesz, I. (2007). Different transmitter transients underlie presynaptic cell type specificity of GABAA,slow and GABAA,fast. Proc. Natl. Acad. Sci. USA *104*, 14831–14836.

Takahashi, H., Katayama, K., Sohya, K., Miyamoto, H., Prasad, T., Matsumoto, Y., Ota, M., Yasuda, H., Tsumoto, T., Aruga, J., and Craig, A.M. (2012). Selective control of inhibitory synapse development by Slitrk3-PTPδ trans-synaptic interaction. Nat. Neurosci. *15*, 389–S2.

Tretter, V., Jacob, T.C., Mukherjee, J., Fritschy, J.-M., Pangalos, M.N., and Moss, S.J. (2008). The clustering of GABA(A) receptor subtypes at inhibitory synapses is facilitated via the direct binding of receptor alpha 2 subunits to gephyrin. J. Neurosci. *28*, 1356–1365.

Twelvetrees, A.E., Yuen, E.Y., Arancibia-Carcamo, I.L., MacAskill, A.F., Rostaing, P., Lumb, M.J., Humbert, S., Triller, A., Saudou, F., Yan, Z., and Kittler, J.T. (2010). Delivery of $GABA_A$Rs to synapses is mediated by HAP1-KIF5 and disrupted by mutant huntingtin. Neuron *65*, 53–65.

Tyagarajan, S.K., and Fritschy, J.-M. (2014). Gephyrin: a master regulator of neuronal function? Nat. Rev. Neurosci. *15*, 141–156.

Uezu, A., Kanak, D.J., Bradshaw, T.W.A., Soderblom, E.J., Catavero, C.M., Burette, A.C., Weinberg, R.J., and Soderling, S.H. (2016). Identification of an elaborate complex mediating postsynaptic inhibition. Science *353*, 1123–1129.

Vaccaro, V., Devine, M.J., Higgs, N.F., and Kittler, J.T. (2017). Miro1-dependent mitochondrial positioning drives the rescaling of presynaptic Ca2+ signals during homeostatic plasticity. EMBO Rep. *18*, 231–240.

Varoqueaux, F., Jamain, S., and Brose, N. (2004). Neuroligin 2 is exclusively localized to inhibitory synapses. Eur. J. Cell Biol. *83*, 449–456.

Vicini, S., Ferguson, C., Prybylowski, K., Kralic, J., Morrow, A.L., and Homanics, G.E. (2001). GABA(A) receptor alpha1 subunit deletion prevents developmental changes of inhibitory synaptic currents in cerebellar neurons. J. Neurosci. *21*, 3009–3016.

Vithlani, M., Terunuma, M., and Moss, S.J. (2011). The dynamic modulation of GABA(A) receptor trafficking and its role in regulating the plasticity of inhibitory synapses. Physiol. Rev. *91*, 1009–1022.

Woo, J., Kwon, S.-K., Nam, J., Choi, S., Takahashi, H., Krueger, D., Park, J., Lee, Y., Bae, J.Y., Lee, D., et al. (2013). The adhesion protein IgSF9b is coupled to neuroligin 2 via S-SCAM to promote inhibitory synapse development. J. Cell Biol. *201*, 929–944.

Xiong, W., Grillet, N., Elledge, H.M., Wagner, T.F.J., Zhao, B., Johnson, K.R., Kazmierczak, P., and Müller, U. (2012). TMHS is an integral component of the mechanotransduction machinery of cochlear hair cells. Cell *151*, 1283–1295.

Yamasaki, T., Hoyos-Ramirez, E., Martenson, J.S., Morimoto-Tomita, M., and Tomita, S. (2017). GARLH family proteins stabilize $GABA_A$ receptors at synapses. Neuron *93*, 1138–1152.e6.

Yee, B.K., Keist, R., von Boehmer, L., Studer, R., Benke, D., Hagenbuch, N., Dong, Y., Malenka, R.C., Fritschy, J.-M., Bluethmann, H., et al. (2005). A schizophrenia-related sensorimotor deficit links alpha 3-containing $GABA_A$ receptors to a dopamine hyperfunction. Proc. Natl. Acad. Sci. USA *102*, 17154–17159.

Yizhar, O., Fenno, L.E., Prigge, M., Schneider, F., Davidson, T.J., O'Shea, D.J., Sohal, V.S., Goshen, I., Finkelstein, J., Paz, J.T., et al. (2011). Neocortical excitation/inhibition balance in information processing and social dysfunction. Nature *477*, 171–178.

Cell Reports, Volume *21*

Supplemental Information

An Essential Role for the Tetraspanin LHFPL4 in the Cell-Type-Specific Targeting and Clustering of Synaptic $GABA_A$ Receptors

Elizabeth C. Davenport, Valentina Pendolino, Georgina Kontou, Thomas P. McGee, David F. Sheehan, Guillermo López-Doménech, Mark Farrant, and Josef T. Kittler

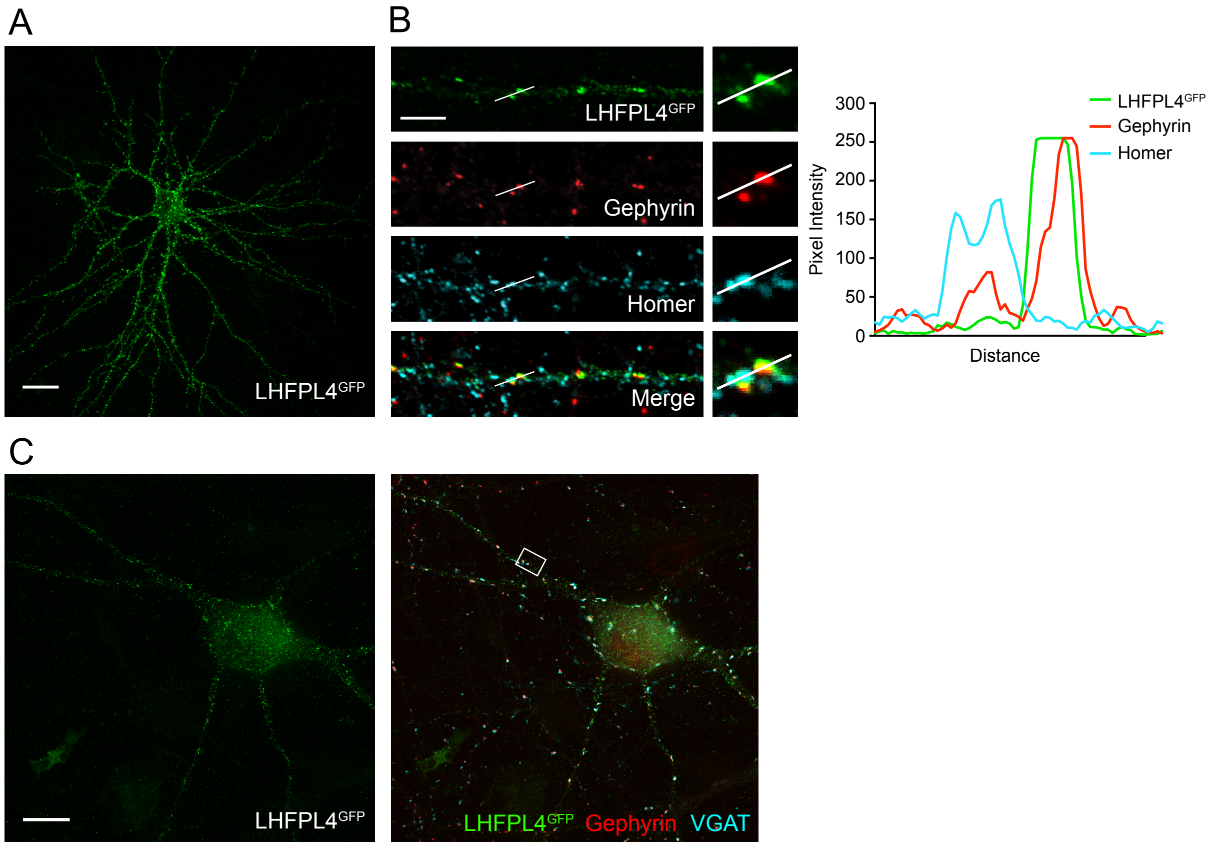

Figure S1, related to Figure 1.
LHFPL4 Specifically Localizes to Inhibitory Synapses.

(A) A maximum intensity projection confocal stack of a DIV16 rat hippocampal neuron transfected with LHFPL4^{GFP}. LHFPL4^{GFP} forms distinct clusters along the dendrites and soma. Scale, 20 µm.
(B) Single section confocal zoom images of dissociated hippocampal dendrites transfected with LHFPL4^{GFP} and labeled with antibodies against gephyrin and homer. Graph shows a fluorescence intensity line scan through both gephyrin and homer clusters for each channel as a function of distance. Scale, 5 µm.
(C) SIM images of dissociated hippocampal neurons transfected with LHFPL4^{GFP} and labeled with antibodies against gephyrin and VGAT. Zoom images in Figure 1 are generated from this cell. Scale, 10 µm.

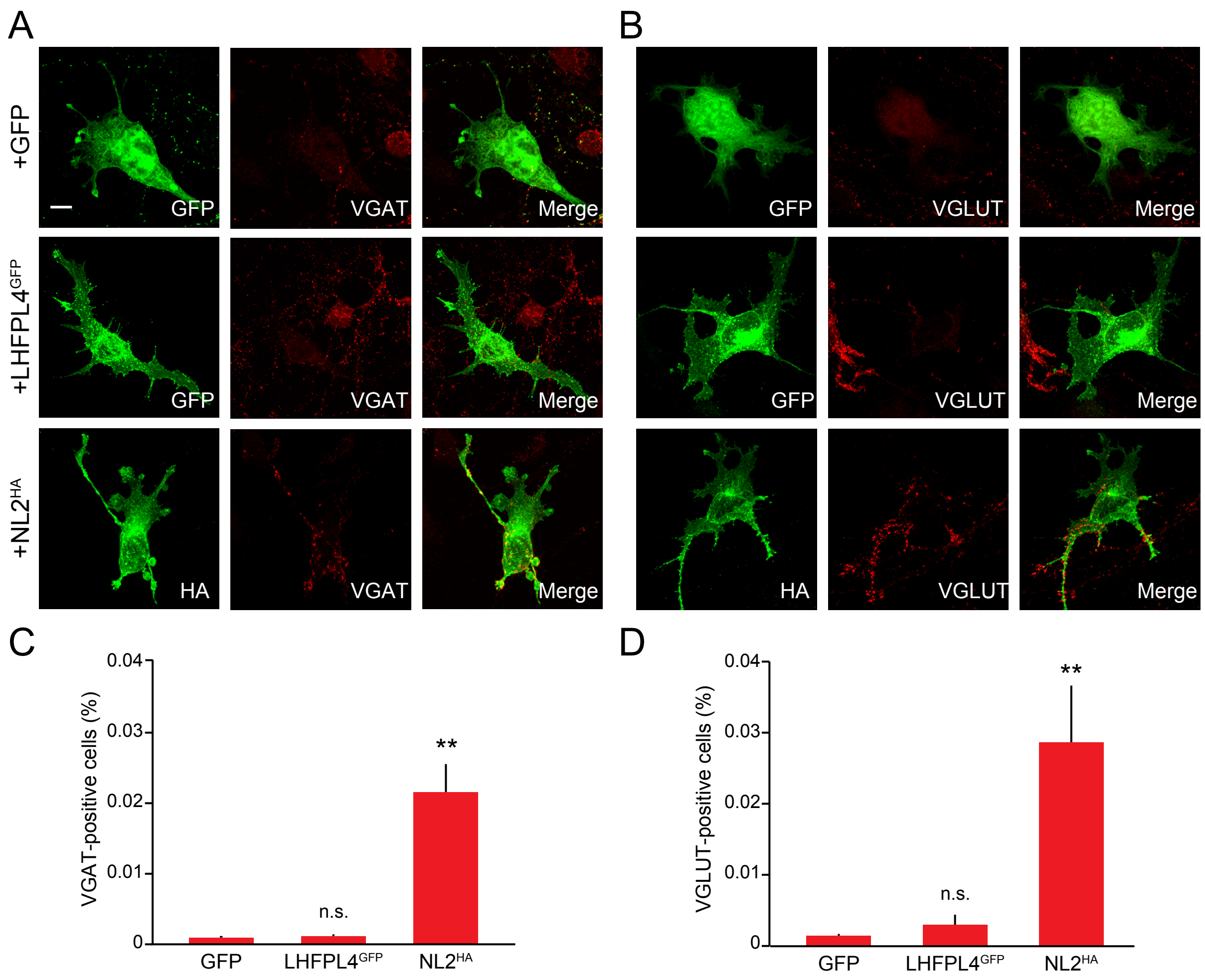

Figure S2, related to Figure 3.
LHFPL4 Does Not Drive the Formation of New Synapses.

(A, B) Representative images of COS-7 cells transfected with GFP, LHFPL4^{GFP} or neuroligin2^{HA} (NL2), co-cultured with dissociated rat hippocampal neurons and labelled with antibodies against (A) VGAT or (B) VGLUT to label inhibitory and excitatory hemi-synapses respectively.
(C, D) Quantification of the proportion of transfected COS-7 cells positive for (C) VGAT or (D) VGLUT. Bars indicate mean and error bars s.e.m. The known synaptogenic molecule NL2 induces the formation of both inhibitory and excitatory hemisynapses whereas LHFPL4 is not different from control. (VGAT hemi-synapses: GFP, 0.10 ± 0.02; LHFPL4^{GFP}: 0.12 ± 0.02; NL2^{HA}, 2.16 ± 0.4; VGLUT hemi-synapses: GFP, 0.14 ± 0.03; LHFPL4^{GFP}: 0.30 ± 0.14; NL2^{HA}, 2.86 ± 0.80). n = 6 coverslips per condition. Kruskal-Wallis one way ANOVA. Asterisks on figure indicate results of Dunn's multiple comparison tests. Scale, 2 μm. **$p < 0.01$.

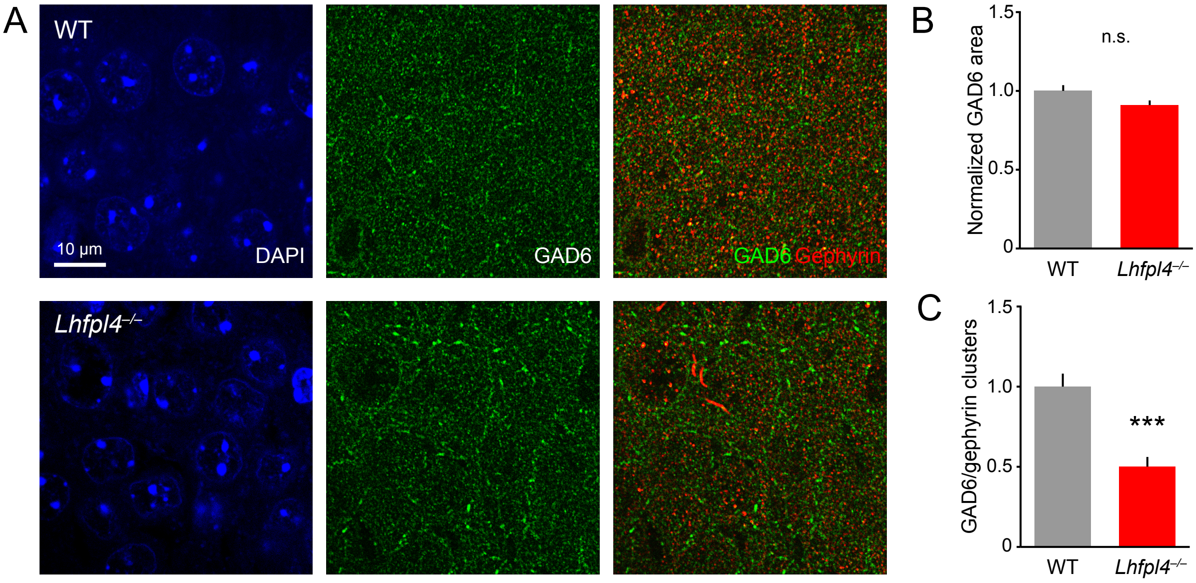

Figure S3, related to Figure 4.
LHFPL4 is Necessary for Gephyrin and $GABA_AR$ Clustering but not GAD6 Clustering in Intact Brain.

(A) Confocal images of adult WT and *Lhfpl4*$^{-/-}$ hippocampal brain sections immunolabelled with antibodies to the inhibitory pre and postsynaptic markers GAD6 and gephyrin respectively and co-stained with DAPI to label cell bodies. Merged images are presented so GAD6/gephyrin positive puncta can be identified as orange.
(B) Quantification of normalized total GAD6 cluster area showing no change in clustering in *Lhfpl4*$^{-/-}$ tissue compared to WT (from 1.0 ± 0.04 to 0.9 ± 0.03, n = 16 WT and 12 KO hippocampi; p = 0.066). Bars indicate mean and error bars s.e.m.
(C) Quantification of normalized GAD6/gephyrin positive cluster number showing a significant decrease in colocalised puncta in *Lhfpl4*$^{-/-}$ tissue compared to WT, indicating fewer inhibitory synapses in *Lhfpl4*$^{-/-}$ tissue (from 1.0 ± 0.08 to 0.5 ± 0.05, n = 16 WT and 12 KO hippocampi; p = 0.00005). Bars indicate mean and error bars s.e.m. Background has been subtracted from images so clusters can be easily identified. Scale bar, 10 µm.
***p < 0.001 (Welch t-test).

Supplemental Experimental Procedures

Animals

The *Lhfpl4* (NM_177763) standard knockout mouse line was obtained from Lexicon pharmaceuticals. Animals were maintained under controlled conditions (temperature 20 ± 2 °C; 12 hour light-dark cycle). Food and water were provided *ad libitum*. Standard genotyping was carried out, briefly the DNA was extracted from ear biopsies and PCRs were performed with the following primers: WT forward (a^F): GAGCACTACATGCGGAACTCGC; WT reverse (a^R): CCCGAGCTTTCAACATGAGGG; mutant forward ($laczex^F$): CGATTTGGCTACATGACATCAACC. WT and *Lhfpl4*$^{-/-}$ animals were generated from *Lhfpl4*$^{+/-}$ x *Lhfpl4*$^{+/-}$ crosses. Both male and female mice were used. WT Sprague-Dawley rats were maintained under the same conditions. All procedures for the care and treatment of animals were in accordance with the Animals (Scientific Procedures) Act 1986.

Constructs

Human LHFPL4-GFP, tagged at the N-terminus, was generated by cloning the coding sequence into pDEST53GFP (Invitrogen) using the Gateway Cloning System (Invitrogen). Mouse LHFPL4-turboGFP, tagged at the C-terminus, was purchase from Origene (MG203120). Super-ecliptic pHlourin (SEP)-tagged $GABA_AR$ subunits α2, β3 and γ2 were a gift from Stephen Moss (Tufts University) (Jacob et al., 2005; Tretter et al., 2008). pEGFP-C1 was purchased from Clontech.

Antibodies

The following primary antibodies were used: rabbit anti-LHFPL4 (Sigma, HPA041421, ICC 1:200, WB 1:250), goat anti-LHFPL4 (Santa Cruz, C-17 sc-99499, IP 2 μg), mouse anti-gephyrin (Synaptic Systems, 147011, ICC 1:500), rabbit anti-gephyrin (Synaptic Systems, 147102, IHC 1:250), mouse anti-gephyrin (BD Biosciences, 610585, WB 1:500), rabbit anti-homer (Synaptic Systems, 160002, ICC 1:500), rabbit anti-VGAT (Synaptic Systems, 131003, ICC 1:1000), guinea pig anti-VGLUT (Synaptic Systems, 135304, ICC 1:1000), guinea pig anti-$GABA_AR$ γ2 (Synaptic Systems, 224004, ICC 1:500), rabbit anti-$GABA_AR$ α2 (Synaptic Systems, 224103, ICC 1:500), mouse anti-$GABA_AR$ α1 (Neuromab N95/35, 75-136, WB 1:200), rabbit anti-$GABA_AR$ β3 was raised against MBP-β3-(345-408) and purified on a GST-β3-(345-408) column generated in house, WB 1:100), guinea pig anti-MAP2 (Synaptic Systems, 188004, ICC 1:1000, IHC 1:500), mouse anti-GAD6 was obtained from GAD6 hybridoma cells (ICC 1:200), mouse anti-CAMKIIα (Merck Millipore, 05-532, ICC 1:500), rabbit anti-neuroligin2 (Synaptic Systems, 129202, WB 1:1000, IP 2 μg), mouse anti-β-tubulin (Sigma, T5293, WB 1:1000), rat anti-GFP (Nacalai-Tesque, GF090R, 1:2000), mouse anti-GFP (Neuromab, N86/8 73-131, WB 1:100, IP 2 μg), rabbit anti-turbo GFP (Evrogen, AB513, 1:1000), mouse anti-HA was obtained from 12CA5 hybridoma cells (ICC 1:100). Secondary antibodies were conjugated to Alexa Fluor 488, 568, 647 (1:1000, Molecular Probes) for immunochemistry and anti-mouse or anti-rabbit HRP conjugated for western blotting (Jackson Laboratories, 1:10 000).

Primary Hippocampal Culture

Cultures of cortical and hippocampal neurons were prepared from E16 mouse or E18 Sprague-Dawley rat embryos as described previously (Arancibia-Carcamo et al., 2009; Pathania et al., 2014). Briefly, rat or mouse hippocampi were dissected from embryonic brains in ice-cold HBSS (GIBCO) supplemented with 10mM HEPES and triturated in the presence of 0.25 % trypsin. The dissociated cells were plated on poly-L-lysine coated coverslips in MEM (GIBCO) containing 10 % horse serum, 10 mM sodium pyruvate and 0.6 % glucose at a density of $8.5x10^5$ or $3.5x10^5$ cells/6 cm dish for mouse and rat neurons respectively. The following day the serum-containing medium was replaced with Neurobasal medium (GIBCO) containing 2 % B-27 (GIBCO), 2 mM glutaMAX (GIBCO), 100 μg/mL penicillin and 100 μg/mL streptomycin.

Immunocytochemistry and Immunohistochemistry

Cultured neurons were fixed between DIV14 and DIV16 in 4 % PFA (PBS, 4 % paraformaldehdye, 4 % sucrose, pH 7) for 7 minutes then permeablized for 10 minutes in block solution (PBS, 10 % horse serum, 0.5 % BSA, 0.2 % Triton X-100). Coverslips were incubated with primary antibody diluted in block solution for 1 hour. They were washed in PBS then incubated for another hour with secondary antibody. Finally coverslips were washed and mounted onto glass slides using ProLong Gold antifade reagent (Invitrogen). For surface labeling, block solution was used without detergent.

For immunohistochemistry, adult mouse brains of either sex were fixed in 4 % PFA overnight and cryoprotected in 30 % sucrose/PBS solution overnight before freezing at -80 °C. The brain samples were

embedded in tissue freezing compound (OCT) and 30 µm brain sections were generated using a Cryostat (Bright Instruments). Free floating thin sections were permeablised for 4-5 hours in block solution (PBS, 10 % horse serum, 3 % BSA, 0.5 % Triton X-100, 0.2 M glycine) then incubated with primary antibody diluted in block solution overnight at 4 °C. For mouse primary antibodies, slices were first incubated overnight at 4 °C with mouse Fab fragment (1:25 with block solution; Jackson ImmunoResearch, 115-007-003) to reduce background staining on the mouse tissue. Slices were washed 4-5 times in PBS for 2 hours then incubated for 3-4 hours with secondary antibody at room temperature. The slices were then washed 4-5 times in PBS for 2 hours and mounted onto glass slides using ProLong Gold antifade reagent (Invitrogen). For GABA receptor labeling, mouse brains were snap frozen in isopentane cooled with dry ice and 30 µm brain sections were generated as above. The thin sections were mounted on cold frosted microscope slides at -16 °C. Slices were fixed in 4 % PFA for 90 seconds, then permeablised for 3-4 hours in block solution and incubated with primary and secondary antibodies as above for brain sections. A coverslip was mounted on top using ProLong Gold antifade reagent (Invitrogen). All solutions were prepared on the day of the experiment (Schneider Gasser et al., 2006).

Confocal Image Analysis

Line scans used for protein localization were performed in ImageJ using the PlotProfile function (NIH, Bethesda, MD, USA), pixel intensity was calculated as a function of distance along a manually drawn line and plotted on a graph. Cluster analysis was carried out using Metamorph (Molecular Devices, Sunnyvale, CA, USA). Analysis was carried out on the zoom images and then averaged to give a value per cell or per brain. For cultured cells, the length of dendrite was traced to generate a dendritic region of interest (ROI). This ROI was transferred to all cluster channels. A user-defined threshold was then applied to each cluster channel and regions were generated around the thresholded area within the dendrite ROI. Number of regions and total area of regions per 30 µm of dendrite were quantified as a readout for synaptic clusters. Clusters smaller than 0.1 μm^2 were excluded from the number of regions analysis. Thresholds were set individually for each cluster channel and kept constant across treatment conditions within an experiment. For brain sections labeled with antibody against $GABA_AR$-α2, the above analysis was performed on each whole zoomed region and averaged for each hippocampi. For brain sections labeled with antibodies against gephyrin and GAD6, where discrete clusters are clear in both WT and *Lhfpl4*$^{-/-}$ tissue, the Synapse Counter plugin for ImageJ (NIH, Bethesda, MD, USA) was used. Background subtraction and max filter parameters were set to 10 and 1 respectively. Clusters greater than 0.095 μm^2 and less than 1 μm^2 were considered for total cluster area analysis. For gephyrin aggregates clusters were manually counted.

Live Super-ecliptic pHlourin (SEP) Imaging

DIV14–16 hippocampal cells transfected with $GABA_AR$-$\alpha2^{SEP}$ were live imaged under perfusion (4 mL/min) in imaging media (125 mM NaCl, 5 mM KCl, 1 mM $MgCl_2$, 2mM $CaCl_2$, 10 mM D-glucose, 10 mM HEPES, adjusted to pH 7.4 with 5 M NaOH) at 37 °C using an Olympus microscope (BX51WI) with a 60X Olympus objective coupled to an EM-CCD camera (Ixon; Andor). Excitation was provided from a metal-halide lamp (X-Cite120, EXFO). Appropriate filters were chosen for SEP-tagged constructs. Movies were recorded at 8.5 Hz. 10 minute movies were acquired where low pH imaging media (pH 5.3) was perfused onto the cells between 3 and 7 minutes. For live cell cluster analysis, single frame images of transfected WT and *Lhflp4*$^{-/-}$ cells were captured. Blind analysis was then carried out where images were manually scored as having clustered or diffuse $GABA_AR$-$\alpha2^{SEP}$ surface fluorescence.

Co-culture assay

Dissociated rat hippocampal neurons were prepared as described above. At DIV8 COS-7 cells were transfected with the candidate synaptogenic protein. Following 24 hours of expression, COS-7 cells were trypsinised and added to the DIV9 neurons. Approximately 24 hours after co-culture, cells were fixed and stained for appropriate synaptic markers using the immunocytochemistry protocol above. Transfected non-neuronal cells and synaptic labeling were imaged using a Zeiss LSM700 upright confocal microscope. For quantification, both the cell fill and synaptic marker channels were individually thresholded to create two binary images. The images were overlaid and the proportion of the cell pixels that were also positive for a synapse pixel were considered hemi-synapses.

Preparation of Brain Lysates

Adult WT and *Lhflp4*$^{-/-}$ male or female whole brains or cortical regions were homogenized on ice in lysis buffer (50 mM HEPES pH 7.5, 0.5 % Triton-X-100, 150 mM NaCl, 1 mM EDTA, 1 mM PMSF in the presence of antipain, pepstatin and leupeptin) then left to rotate at 4 °C for 2 hours. Membranes were pelleted by ultracentrifugation at 38000 g for 40 minutes at 4 °C. Protein content of the supernatant was assayed by BioRad protein assay.

Co-immunoprecipitation Assays

4 mg of brain lysate or transfected COS-7 cell lysates were transferred to a 1.5 ml microcentrifuge tube and made up to 500 µl with lysis buffer (see above). For GFP/SEP co-immunoprecipitations, GFP/SEP-tagged protein complexes were precipitated from transfected cell lysates with 10 µl of 50 % GFP TRAP bead slurry (Chromtech) for one hour at 4 °C. For co-immunoprecipitations from brain lysate, 2 µg of a primary antibody was added and incubated with the lysate overnight at 4 °C with rotation. Complexes were precipitated with 15 µl of 50 % protein A (for rabbit antibodies) or protein G (for goat antibodies) sepharose bead slurry (Generon) for one hour at 4 °C. All beads were then washed 3 times by centrifugation and resuspension in 1 ml of lysis buffer. Samples were suspended in 3X protein sample buffer and analyzed by SDS-PAGE and western blotting. Briefly, protein samples were separated by SDS-PAGE on 10 % Tris-Glycine gels and blotted onto nitrocellulose membranes (GE Healthcare Bio-Sciences). Membranes were blocked for 1 hour in milk (PBS, 0.05 % Tween, 4 % milk), incubated in primary antibodies diluted with milk overnight at 4 °C before incubation in an appropriate HRP-conjugated secondary antibody for 1 hour at room temperature. The blots were developed with an ECL-Plus detection reagent (GE Healthcare Bio-Sciences). Densitometric analysis was performed in ImageJ (NIH).

Surface Biotinylation Assays

Surface biotinylation assays have been fully described previously (Smith et al., 2014; Twelvetrees et al., 2010). Briefly, cultured cortical cultures were kept at 4 °C and rinsed with PBS (all washing steps were performed in PBS supplemented with 1 mM $CaCl_2$ and 0.5 mM $MgCl_2$) before incubation with 0.5 mg/ml EZ-Link Sulfo-NHS-SS-Biotin (Pierce) for 12 minutes. The surface biotinylation reaction was quenched using three washes of 1 mg/ml BSA in Mg^{2+}/Ca^{2+} supplemented PBS. Cells were washed and solubilized in 360 µl of RIPA buffer (50 mM Tris pH 7.5, 1 mM EDTA, 2 mM EGTA, 150 mM NaCl, 1 % NP40, 0.5 % DOC, 0.1 % SDS, 1 mM PMSF with antipain, pepstatin and leupeptin) for 1 hour at 4 °C. Cell membranes were pelleted at 14 000 rpm for 10 minutes at 4 °C. 60 µl (20 %) was kept to analyze as an input. The remainder was incubated with 50 µl of UltraLink NeutrAvidin 50 % bead slurry (Pierce) for 2 hours at 4 °C. Beads were washed twice with high salt (0.5 M NaCl) RIPA buffer and once with normal (150 mM NaCl) RIPA buffer. Complexes were separated by SDS-PAGE and analyzed by western blotting as above. $GABA_ARs$ were detected with rabbit anti-$GABA_AR$-β3 subunit as primary antibody.

Electrophysiology in Dissociated Cultures

Cultured cells were viewed using an upright microscope (BX50WI; Olympus) and perfused with extracellular solution containing the following (in mM): 145 NaCl, 2.5 KCl, 2 $CaCl_2$, 1 $MgCl_2$, 10 glucose, and 10 HEPES, adjusted to pH 7.3 with NaOH. To block voltage-gated sodium channels, NMDA-, AMPA- and glycine receptors, 0.5 µM TTX, 20 µM D-AP5, 20 µM NBQX and 1 µM strychnine were included. Recording pipettes were pulled (Narishige PC10) from thick-walled borosilicate glass tubing (1.5-mm outer diameter, 0.86-mm inner diameter; Harvard Apparatus), coated with Sylgard (Dow Corning 184) and fire-polished before use. Pipettes were filled with the following internal solution (in mM): 128 CsCl, 10 HEPES, 10 EGTA, 10 tetraethylammonium chloride (TEA-Cl), 2 MgATP, 1 $CaCl_2$, 2 NaCl, 1 QX-314 (Tocris Bioscience), pH 7.4 with CsOH (final resistance ∼4–8 MΩ). Currents were recorded at 23–26 °C using an Axopatch 200A amplifier, low-pass filtered at 5 kHz and digitized at 20 kHz using WinEDR (Strathclyde Electrophysiology Software) and ITC-18 interface (InstruTECH). Series resistance and input capacitance were read directly from the amplifier settings used to minimize the current responses to 5 mV hyperpolarizing voltage steps. Series resistance was typically compensated by 60–80% and data were discarded if the series resistance varied by >20%. The "phasic charge transfer" due to mIPSCs was calculated using an automated procedure (written in IGOR Pro 6.35; WaveMetrics) that avoided subjective decisions regarding detection or selection of individual synaptic currents. The record was split into segments (typically 1s) and for each segment an all-point amplitude histogram was generated and fit with a single-sided Gaussian to the most-positive current values. The position of the peak of the histogram was taken as the baseline current for that segment and subtracted from the record. The integral of the subtracted current provided the charge carried by the synaptic events. The total charge was divided by the recording time to give a measure of phasic charge transfer per second.

Acute Hippocampal Slice Electrophysiology

To prepare acute brain slices, male and female mice (P30–45) were deeply anesthetized with isoflurane and decapitated. The brain was removed and submerged in ice-cold slicing solution containing (in mM): 85 NaCl, 2.5 KCl, 7 $MgCl_2$, 1.25 NaH_2PO4, 0.5 $CaCl_2$, 25 $NaHCO_3$, 25 glucose, and 75 sucrose, saturated with 95% O_2/5% CO_2, pH 7.4. Coronal hippocampal slices (300 µm thick) were prepared using a vibratome (Campden 7000smz). Slices were maintained at 32.5 °C for 30 min and then at room temperature for at

least 30 min to recover. During this period the slicing solution in the holding chamber was replaced (~5 ml min^{-1}) with a recording solution containing (in mM): 125 NaCl, 2.5 KCl, 1 $MgCl_2$, 1.25 NaH_2PO_4, 2 $CaCl_2$, 38 glucose, and 26 $NaHCO_3$ saturated with 95% O_2/5% CO_2, pH 7.4. For recording, slices were placed in a submerged chamber on the stage of an upright microscope (Scientifica SliceScope). Phasic charge transfer was analyzed as described above. Spontaneous currents were detected using a scaled template algorithm based on rising and decaying exponentials (Clements and Bekkers, 1997) (NeuroMatic 2.9, http://www.neuromatic. thinkrandom.com/). When analyzing current frequency, all events were counted, irrespective of overlapping decays. For kinetic analysis, only events with a monotonic rise and uncontaminated decay were included; they were aligned on their rising phase before averaging. The decay of averaged fast mIPSCs was described by the sum of two exponentials, from which the weighted time constant of decay (τ_w, decay) was calculated as the sum of the time constants weighted by their fractional amplitudes. Slow mIPSCs were detected using a template with a rise of 10 ms and a decay of 50 ms. To reduce error when measuring the rise and decay time of these slow currents, individual events were fitted with an empirical equation:

$$f(\mathrm{x}) = \left(1 * \exp\left(-\frac{\mathrm{x} - \mathrm{X0}}{\tau_{\mathrm{rise}}}\right)\right)^{N} * \left(A_{\mathrm{f}} * \exp\left(-\frac{\mathrm{x} - \mathrm{X0}}{\tau_{\mathrm{f}}}\right) + A_{\mathrm{s}} * \exp\left(-\frac{\mathrm{x} - \mathrm{X0}}{\tau_{\mathrm{s}}}\right)\right)$$

and 10–90% rise time and 63% decay time measures were taken from the fit waveform (Bekkers and Clements, 1999; Bekkers and Stevens, 1996).

For mIPSCs and tonic current measurement, recording pipettes were filled with an internal solution containing (in mM): 128 CsCl, 10 EGTA-Cs, 10 HEPES, 2 MgATP, 1 $CaCl_2$, 2 NaCl, 1 QX-314 (Tocris Bioscience), and 5 TEA-Cl, adjusted to pH 7.3 with CsOH. In some cases this solution also contained 0.2% biocytin (Molecular Probes). D-AP5 (20 μM, Abcam) and NBQX (10 μM, Abcam) were added to the external solution to block NMDARs and AMPARs. All mIPSCs were blocked by bicuculline (20 μM, Tocris) or gabazine (20 μM, Abcam). To record mEPSCs, NBQX was omitted and bicuculline (20 μM) or gabazine (20 μM) were added to the external solution. As the mEPSC frequency appeared low under these conditions, all data were obtained under conditions designed to increase mEPSC frequency and thus maximize the number of recordings with an acceptable number of events. Thus, we increased extracellular K^+ (additional 5 mM KCl), made recordings at an elevated temperature (32.5 °C), and used an internal solution containing (in mM) 135 Cs-gluconate, 10 HEPES, 10 Na-phosphocreatine, 4 MgATP, 0.4 NaGTP, 2 QX-314, and 10 TEA-Cl, adjusted to pH 7.3 with CsOH.

After recording, slices were fixed overnight at 4 °C in a solution containing 4% PFA. Slices were then washed in PBS 1x and incubated for 3 hours at room temperature in a solution containing 1:1000 triton X-100 with1:1000 strepavidin-555 (Molecular Probes). Slices were then washed three times in PBS, incubated for 10 minutes at room temperature with DAPI (1:1000) and mounted using anti-Fade gold (Invitrogen P36930). Confocal images were acquired on a Leica TCS SPE upright confocal microscope using a 20X oil objective (NA: 0.6) and digitally captured using LAS software.

Supplemental References

Arancibia-Carcamo, I.L., Yuen, E.Y., Muir, J., Lumb, M.J., Michels, G., Saliba, R.S., Smart, T.G., Yan, Z., Kittler, J.T., and Moss, S.J. (2009). Ubiquitin-dependent lysosomal targeting of $GABA_A$ receptors regulates neuronal inhibition. Proc. Natl. Acad. Sci. *106*, 17552–17557.

Bekkers, J.M., and Clements, J.D. (1999). Quantal amplitude and quantal variance of strontium-induced asynchronous EPSCs in rat dentate granule neurons. J. Physiol. *516*, 227–248.

Bekkers, J.M., and Stevens, C.F. (1996). Cable properties of cultured hippocampal neurons determined from sucrose-evoked miniature EPSCs. J. Neurophysiol. *75*, 1250–1255.

Jacob, T.C., Bogdanov, Y.D., Magnus, C., Saliba, R.S., Kittler, J.T., Haydon, P.G., and Moss, S.J. (2005). Gephyrin regulates the cell surface dynamics of synaptic $GABA_A$ receptors. J. Neurosci. *25*, 10469–10478.

Pathania, M., Davenport, E.C., Muir, J., Sheehan, D.F., López-Doménech, G., and Kittler, J.T. (2014). The autism and schizophrenia associated gene CYFIP1 is critical for the maintenance of dendritic complexity and the stabilization of mature spines. Transl. Psychiatry *4*, e374.

Schneider Gasser, E.M., Straub, C.J., Panzanelli, P., Weinmann, O., Sassoè-Pognetto, M., and Fritschy, J.-M. (2006). Immunofluorescence in brain sections: simultaneous detection of presynaptic and postsynaptic proteins in identified neurons. Nat. Protoc. *1*, 1887–1897.

Smith, K.R., Davenport, E.C., Wei, J., Li, X., Pathania, M., Vaccaro, V., Yan, Z., and Kittler, J.T. (2014). GIT1 and βPIX are essential for $GABA_A$ receptor synaptic stability and inhibitory neurotransmission. Cell Rep. *9*, 298–310.

Tretter, V., Jacob, T.C., Mukherjee, J., Fritschy, J.-M., Pangalos, M.N., and Moss, S.J. (2008). The clustering of GABA(A) receptor subtypes at inhibitory synapses is facilitated via the direct binding of receptor alpha 2 subunits to gephyrin. J. Neurosci. *28*, 1356–1365.

Twelvetrees, A.E., Yuen, E.Y., Arancibia-Carcamo, I.L., MacAskill, A.F., Rostaing, P., Lumb, M.J., Humbert, S., Triller, A., Saudou, F., Yan, Z., et al. (2010). Delivery of $GABA_A$Rs to synapses is mediated by HAP1-KIF5 and disrupted by mutant huntingtin. Neuron *65*, 53–65.
